# Gender-related variables for health research

**DOI:** 10.1186/s13293-021-00366-3

**Published:** 2021-02-22

**Authors:** Mathias W. Nielsen, Marcia L. Stefanick, Diana Peragine, Torsten B. Neilands, John P. A. Ioannidis, Louise Pilote, Judith J. Prochaska, Mark R. Cullen, Gillian Einstein, Ineke Klinge, Hannah LeBlanc, Hee Young Paik, Londa Schiebinger

**Affiliations:** 1 Department of Sociology, University of Copenhagen, Øster Farimagsgade 5, Bld. 16, DK-1014 Copenhagen K, Denmark; 2 Stanford Prevention Research Center, Department of Medicine, Stanford University, 1265 Welch Rd, Stanford, CA 94305-5411 USA; 3 Department of Psychology, University of Toronto, 100 St George St, Toronto, ON M5S 3G3 Canada; 4 Department of Medicine, University of California, San Francisco, 550 16th Street, 3rd Floor, San Francisco, CA 94143 USA; 5 Department of Medicine, McGill University Health Center, McGill University, 5252 De Maisonneuve Blvd, Office 2B.39, Montréal, QC H4A 3S5 Canada; 6 Stanford Center for Population Health Sciences, Stanford University, 1701 Page Mill Road, Palo Alto, 94304 USA; 7 Horizon 2020 Advisory Group for Gender, European Commission, Brussels, Belgium; 8 Department of Science & Technology Studies, Cornell University, 303 Morrill Hall, Ithaca, NY 14853 USA; 9 Center for Gendered Innovations in Science and Technology Research, 405 KSTC, 22 Teheranro-7gil, Gangnam-gu, Seoul, 06130 Republic of Korea; 10 History of Science, Stanford University, Building 200, 450 Jane Stanford Way, Stanford, CA 94305 USA

**Keywords:** Gender measures, Biomedical outcomes, Sex differences

## Abstract

**Background:**

In this paper, we argue for Gender as a Sociocultural Variable (GASV) as a complement to Sex as a Biological Variable (SABV). Sex (biology) and gender (sociocultural behaviors and attitudes) interact to influence health and disease processes across the lifespan—which is currently playing out in the COVID-19 pandemic. This study develops a gender assessment tool—the Stanford Gender-Related Variables for Health Research—for use in clinical and population research, including large-scale health surveys involving diverse Western populations. While analyzing sex as a biological variable is widely mandated, gender as a sociocultural variable is not, largely because the field lacks quantitative tools for analyzing the influence of gender on health outcomes.

**Methods:**

We conducted a comprehensive review of English-language measures of gender from 1975 to 2015 to identify variables across three domains: gender norms, gender-related traits, and gender relations. This yielded 11 variables tested with 44 items in three US cross-sectional survey populations: two internet-based (*N* = 2051; *N* = 2135) and a patient-research registry (*N* = 489), conducted between May 2017 and January 2018.

**Results:**

Exploratory and confirmatory factor analyses reduced 11 constructs to 7 gender-related variables: caregiver strain, work strain, independence, risk-taking, emotional intelligence, social support, and discrimination. Regression analyses, adjusted for age, ethnicity, income, education, sex assigned at birth, and self-reported gender identity, identified associations between these gender-related variables and self-rated general health, physical and mental health, and health-risk behaviors.

**Conclusion:**

Our new instrument represents an important step toward developing more comprehensive and precise survey-based measures of gender in relation to health. Our questionnaire is designed to shed light on how specific gender-related behaviors and attitudes contribute to health and disease processes, irrespective of—or in addition to—biological sex and self-reported gender identity. Use of these gender-related variables in experimental studies, such as clinical trials, may also help us understand if gender factors play an important role as treatment-effect modifiers and would thus need to be further considered in treatment decision-making.

**Supplementary Information:**

The online version contains supplementary material available at 10.1186/s13293-021-00366-3.

## Main text

### Background

Nearly 20 years ago, the U.S. Institute of Medicine (IOM, now the Academies of Science) recognized that sex (biology) matters in determining health outcomes but also that gender (sociocultural behaviors and attitudes) interacts with sex to influence health and disease processes across the lifespan [1, 2]. In the past decade, both the Canadian Institutes of Health Research (2010) [3] and the European Commission (2014) [4] have endorsed integrating sex and gender (usually as male/female binaries) into health research, and the US National Institutes of Health (NIH) has mandated the inclusion of sex as a biological variable (SABV—2016) [5]. Yet still today, sex and gender are often inappropriately conflated in the biomedical literature [6], and gender is rarely considered largely because the field lacks quantitative tools for analyzing the influence of gender on health outcomes.

In this paper, we argue for Gender as a Sociocultural Variable (GASV) as a complement to SABV. Despite several efforts to examine gender and health [7, 8], the field lacks adequate tools to assess gender. To address this problem, we set out to develop a new instrument—the Stanford Gender-Related Variables for Health Research (GVHR).

Our interest was piqued by a 2007 study that reported that men with higher “femininity” scores had lower risk of coronary heart disease. No such relationship was observed among women [9]. Similarly, a 2015 study found that, independent of biological sex, young adults with a gender score more strongly associated with “feminine gender-related characteristics” were more likely to experience a recurrence of acute coronary syndrome (ACS)—regardless of whether they identified as a man or a woman (a non-binary option was not offered) [10, 11].

These innovative studies highlighted how challenging it is to account for both sex and gender in clinical research. Although these tools demonstrated that gender plays a key role as a determinant of health outcomes, its operational definition deserves further consideration. We were concerned that the conclusions were based on outdated gender identity constructs, such as the Bem Sex Role Inventory (BSRI, 1974) [12], developed using predominantly white, higher socioeconomic U.S. undergraduate student participants and based on outdated notions of masculinity and femininity or their cognates. We also saw that the GENESIS-PRAXY gender questionnaire used in the 2015 study was tested in a sample of 909 heart-disease patients and had not been cross-validated in broader patient, or non-patient, populations. We judged that despite its focus on gender, the GENESIS-PRAXY study used logistic regression with biological sex as the outcome to derive its final score of masculine and feminine characteristics. We also found that the internal consistency of the measure had not been explicitly tested.

More importantly, moving forward, it is important to recognize that it is no longer sufficient to reduce gender to a two-dimensional spectrum stretching from “masculinity” to “femininity,” i.e., concepts which were historically construed as complementary oppositions [13], with a man being one thing and a woman being the opposite (for example, rational/emotional; public/private; mind/body). These concepts are too broad and imprecise to be useful in health research. Given that the goal is to provide physicians and policy makers with gender-related health interventions, measures of gender-related behaviors, such as caregiving or risk-taking, should be labeled as such.

To address these limitations, we develop a new gender-variables instrument. This step toward developing more comprehensive and precise survey-based measures of gender, in relation to health, builds on insights from gender theory to capture key aspects of three dimensions of gender that can be deployed quantitatively in diverse clinical research or large health surveys: (1) Gender Norms [14], (2) Gender-Related Traits [15, 16], and (3) Gender Relations [17] (see Tables S1 and S2), each of which may correlate with sex assigned at birth or self-reported gender identity, without a predetermined coding of that behavior as masculine or feminine (see Table 1). Thus, we adopt a multidimensional understanding that seeks to capture how intrapersonal, interpersonal, and institutional aspects of gender intersect to shape people’s health and illness [17].

**Table 1:** Three interrelated dimensions of gender

Gender refers to sociocultural factors that shape the identities, attitudes, behaviors, bodily appearances, and habits of women, men, and gender-diverse individuals. Gender is multidimensional [17] and complex, changing as social norms and values change. Gender also intersects with other sociocultural categories. The gender variables developed here appear alongside self-reported variables collected in our survey, including: sex assigned at birth, self-reported gender identity, sexual orientation, ethnicity, age, individual and household income, and education, among others (see SI text). • Gender Norms consist of legislated and both spoken and unspoken cultural rules produced through social institutions (such as governments, families, schools, workplaces, laboratories, universities, or boardrooms), cultural products (such as technologies, science, literature, and social media), and broader local and global cultures [18, 19]. These norms build upon and reinforce gender stereotypes and perpetuate gendered power relations in workplaces, families and other institutions. They operate as rules and expectations of what behaviors and activities are appropriate for women, men, and gender-diverse individuals in a given social setting [14, 18, 20]. Here, we measure individuals’ adherence to gender norms through self-reported behaviors (not attitudes or stereotypes) • Gender-Related Traits refer to aspects of a person’s gender identity not captured by self-reported categories (such as man, woman, non-binary, and gender-queer) and concern how individuals or groups perceive and present themselves in relation to gender norms [15]. Our key interest is how individuals think and act vis-à-vis cultural meanings ascribed to gender [15, 16]. We measure gender-related traits through self-reported assessments of personality attributes. • Gender Relations refer to power relations, economic relations, affective relations, and symbolic relations (including speech and writing) between individuals of different gender identities as these relate to gender norms [17, 21]. They concern how we interact with people and institutions in the world around us, based on our sex and our gender identity [19, 21, 22]. Gender relations encompass how gender shapes social interactions in romantic relationships, friendships, families, schools, workplaces and public settings, for instance, the power relation between a man patient and woman physician.	

These three meta-categories are not mutually exclusive. Gender is multidimensional: any given individual may experience different configurations of gender norms, traits, and relations that cannot be subsumed into a “masculine” or “feminine” score or considered “fixed” [23].

Our new instrument represents a step toward developing more comprehensive and precise survey-based measures of gender in relation to health. Our questionnaire is designed to shed light on how specific gender-related behaviors and attitudes contribute to health and disease processes, irrespective of—or in addition to—biological sex and self-reported gender identity.

## Methods

In a systematic review of the English-language literature from 1975 to 2015, we identified 74 eligible scales used in gender-related measures. From the 74 scales, we distilled 11 composite gender constructs and developed 44 items to measure gender, which we subjected to exploratory and confirmatory factor analysis (EFA and CFA) in three independent U.S. survey samples. This reduced the original 11 gender-related variables to 7 factors: caregiver strain, work strain, independence, risk-taking, emotional intelligence, social support, and discrimination and the 44 survey items to 25. We then examined the relevance of the derived subscales in allowing for more precise analysis of variations in health-related quality of life, obesity, and risky health behaviors.

### Literature search

We searched PsycINFO, PsycTESTS, and PubMed for all English-language studies using gender-related tests and scales, from 1975 through 2015, to identify existing questionnaires or scales and construct a comprehensive list of typical traits and/or characteristics used in gender-related measures in both psychology and medicine. We screened an initial sample of 2981 articles from PubMed, PsycTESTS, and PsycINFO from which 405 articles were deemed relevant for further interrogation, within which 127 unique gender-related tests and scales were identified. We also screened existing literature reviews published in books and found four additional scales. Altogether, 131 gender-related questionnaires were sorted into three overarching categories for analyzing gender norms, gender-related traits, and gender relations. Further, we checked citation frequencies for each scale to determine how often it has been used in the literature. All gender scales with at least 20 Google Scholar citations within the last 10 years were selected for further investigation, of which 74 scales met the criteria. All articles published from 2006 to 2015 were retained for further investigation irrespective of whether they were cited or not (the search methods, selection criteria, and review procedures are specified in SI text, Figure S1, and Tables S3-S4).

Several limitations made it impossible to simply plug these 74 scales into a new questionnaire. Very few existing scales (*N* = 8) focus on associations between gender and health [24–28]. The eligible gender-related scales are generally restricted to either men or women and assess either masculinity [29] or femininity [30, 31] or both as unipolar or bipolar constructs [12, 32–38] (e.g., hyper-masculinity or hyper-femininity) [26, 28, 39–49]. Further, most scales rely on “agree-disagree” ratings, making them susceptible to acquiescence bias [50].

### Questionnaire development

In developing the questionnaire, we recognized the need to minimize the time burden of completing the questionnaire; therefore, we limited the initial item pool to 3–6 items per construct. To avoid acquiescence bias, we presented our items as construct-specific questions. The 74 scales guided our selection of core characteristics to be included among our gender-related measures.

1. Gender Norms. Guided by three of the 74 eligible scales, respondents’ adherence to gender norms was measured by three composite constructs (caregiver strain, time use, and work strain) consisting of 16 items.
  *Caregiver strain* captures perceived consequences of responsibility for unpaid, long-term caregiving to children, partners, friends, and elderly (excluding housework and caregiving occupations) [51, 52] and consists of three items, adapted from Graessel and colleagues [53], recorded on a five-point scale: emotional exhaustion, physical exhaustion, and worries about the future caused by caregiving for someone in need, such as a child, elder, partner, or disabled family member. Higher scores on these items indicate higher levels of caregiver strain.
  *Time use* measures individual hours spent per day, recorded as open-ended numerical estimates of daily time-spent [54], in the following categories: paid work, household activities, eating and drinking, leisure and sport, caring for others, sleeping, and commuting. The items were adapted from the American Time Use Survey.
  *Work strain* measures job strain and emotional job demands as six items, recorded on a five-point scale: work speed, work repetition, emotional job demands, physical job demands, perceived risk, and physical hazards at work. The first four items were adapted from Karasek and Theorell [55]; the last two were developed by the authors. Higher scores on each item indicate higher levels of work strain.
2. Gender-Related Traits. Guided by 31 of the original 74 eligible scales, gender-related traits were measured as five composite constructs: competitive, risk-taking, independence, communal, and expressive, consisting of 16 items.
  *Competitive* consists of two items, recorded on a five-point scale, asking respondents how often they find themselves competing with others in situations that do not call for competition and how competitive they are in general, compared to others. The first item is modified from Ryckman et al. [56], the second is developed by the authors. Higher scores on each item indicate higher levels of competitiveness.
  *Risk-taking* focuses on physical and behavioral risks, measured by three items adapted from Dohmen et al. [57], recorded on a five-point scale: general risk-taking behavior, risk-taking when making financial decisions, and risk-taking with respect to recreational activities. Higher scores on each item indicate higher levels of risk-taking.
  *Independence* is a personality trait characterized by a focus on the person as an individual, not as part of a community or group, which includes agency, self-confidence, self-determination, and decision-making ability, but not self-control or self-esteem [58]. Independence was based on three items, recorded on a five-point scale, adapted from Bakker et al. [59], Clark et al. [60], and Triandis et al. [61], asking respondents how important it is for them to be independent, how often they turn to others for help when in need, and how important it is for them to solve their problems independently. A higher score indicates a higher level of independence for all three items.
  *Communal* is a trait characterized by a focus on the individual as part of a group or community, an orientation toward relationships and a concern for others’ needs and well-being [62]. We used four items, scored on a five-point scale, asking respondents how often they worry about what other people think about them, how often they take other people’s needs into account when making important decisions, how often friends talk to them about their problems, and how easy it is for them to spot when someone in a group is feeling uncomfortable. Item one was developed by the authors, item two was adapted from Clark et al. [60], and items three and four were adapted from Baron-Cohen and Wheelwright [63]. Higher scores indicate higher levels of communal orientation.
  *Expressive* captures abilities to recognize and express emotions, such as sadness, anger, frustration, compassion, joy, or affection and includes aspects of emotional intelligence, i.e., individuals’ ability to recognize what they feel, manage those emotions, and use emotions in problem solving [64]. We used four items, recorded on a five-point scale, asking respondents how often they talk to friends about their problems, how easy it is for them to understand their own feelings, how easy it is for them to express what they are feeling, and how easy it is for them to ask other people for help when in need. Item one was developed by the authors, item two was adapted from Salovey and colleagues [65], item three was adapted from Gross and John [66], and item four was adapted from Clark and colleagues [60]. Higher scores indicate higher levels of expressivity.
3. Gender Relations. Guided by 40 eligible scales identified in the literature search, gender relations were measured by three composite constructs: social support, discrimination, and quality of family relationships, consisting of 13 items and a single-item measure of personal income.
  *Social support* captures perceived satisfaction with the type (physical, emotional, informational, and financial), the availability, and level of support a person might receive. Support may come from partners, relatives, friends, coworkers, health-care systems, the larger community, etc. Social support consists of four items recorded on a five-point-scale measuring the availability and level of social support a person might receive. We asked respondents how often, within the past year, they had someone they could ask for advice, someone to show them love and affection, someone to help them with daily chores, and how often they felt lonely. Item one, two, and three were adapted from the ENRICHD Social Support Inventory [67] and item four was developed by the authors. Higher scores indicate higher levels of social support.
  *Discrimination* refers to “systemic unfair treatment” and can occur at multiple levels. We measured micro or interpersonal discrimination. Specifically, we asked the respondents how often they had felt discriminated against because of their gender, in general, when getting hired, when at school, when receiving medical care, in other public settings, and in their family. The items were recorded on a five-point-scale, with higher scores indicating more frequent experiences of gender discrimination.
  *Quality of family relationships* measures experiences of harmony and conflict in familial settings, based on two self-developed items recorded on a five-point-scale asking respondents about how they would describe the quality of their relationship with close relatives (in the past year) and how often they had argued with close relatives (in the past year). Higher scores indicate higher levels of perceived quality in family relationships.

### Initial testing of the questionnaire

Content validity of the first draft of the questionnaire was assessed by nine members of the author-group who had not taken part in the construction of the item-list, some with technical expertise in the construction of survey-questionnaires and some with expertise in gender-related aspects of health. Each coauthor was asked to rate each item with respect to its relevance in measuring a given variable construct. Ratings were made on a four-point scale (4 = very relevant, 3 = relevant but needs minor alteration, 2 = unable to assess relevance without major revision, 1 = not relevant). These coauthors were also invited to suggest additional items, if they considered the proposed item-list inadequate in capturing a given construct. To improve item quality, we conducted seven cognitive interviews with people recruited by posters in the local area who varied on demographic markers such as education-level, job, age, ethnicity, and gender. In the interviews, we used verbal probing techniques to identify questions that the interviewees found vague and unclear and to elicit how they arrived at answers to the questions. We did this by asking them to reflect on what the items meant to them, how they would rephrase the question in their own words, how they came up with their answers to the questions, and whether the questions were easy or hard to answer and why.

### Survey participants

Participants were recruited from the USA through two online services and a health research registry: Prolific, Amazon Mechanical Turk, and the Stanford Research Registry, which consists of ~ 4000 former adult patients at the Stanford University Medical Center who have agreed to be contacted for participation in research studies. We used the web-based Qualtrics software to collect data from Prolific (sample 1) in August and September 2017, Mechanical Turk (sample 2) in December 2017 and January 2018, and the Stanford Research Registry in May and June 2017 (sample 3). Sample 1 consisted of 2051 respondents; 1992 completed the survey. Sample 2 consisted of 2135 respondents; 2043 completed the survey. Sample 3 consisted of 489 respondents; 452 completed the survey. Sample characteristics are presented in Table 2.

**Table 2:** Sample characteristics

	Sample 1	*N*	Sample 2	*N*	Sample 3	*N*
	%		%		%	
Sex		2017		2058		454
Male	46.5	937	49.2	1013	32.8	149
Female	53.1	1071	50.6	1041	67.0	304
Intersex	0.1	2	0.0	1	–	–
Other	0	0	0.1	2	0	0
Prefer not to state	0.3	7	0	1	0	1
Gender		2017		2058		454
Man	47.0	948	49.0	1008	33.0	150
Woman	51.9	1047	50.2	1034	66.0	301
Gender fluid/non-binary	1.3	26	0.4	8	0	2
Prefer not to state/other	0.4	8	0.2	5	0	1
Sexual orientation		2017		2058		454
Heterosexual or straight	83.9	1693	88.7	1826	89.4	406
Gay or lesbian	3.7	74	4.1	84	4.2	19
Bisexual	9.9	199	6.0	124	5.3	24
Asexual	1.7	34	0.70	14	0.7	3
Other	0.8	17	0.49	10	0.4	2
Ethnicity/Race		2014		2059		454
White	79.9	1610	83.2	1709	74.4	338
Hispanic, Latinx or Spanish	5.8	117	5.6	116	15.6	71
Black or African American	8.4	169	8.3	171	5.3	24
Asian	10.3	207	5.7	117	11.5	52
Native American or Alaska Native	2.4	49	2.4	49	2.2	10
Middle Eastern or North African	0.1	13	0.0	10	2.2	11
Native Hawaiian or Pacific Islander	0.0	2	0.0	3	1.1	5
Other	0.1	17	0.1	15	2.2	11
Birth year, Sample 1 (Mean = 1982; Standard deviation = 12.16; Median = 1984 ; range = 1937–1999; interquartile range = 15) Birth year, Sample 2 (Mean = 1973; Standard deviation = 13.72; Median = 1971; range = 1936–1999; interquartile range = 23) Birth year, Sample 3 (Mean = 1969; Standard deviation = 17.01; Median = 1969; range = 1921–1998; interquartile range = 27)						
Relationship status		2017		2059		452
Living with a romantic partner	49.2	993	58.1	1196	55.1	249
Not living with a romantic partner	50.8	1024	41.9	863	44.9	203
Education		1995		2052		452
High school degree or lower	12.7	253	9.7	200	2.8	13
Some college/associate degree/technical degree	33.0	659	38.5	792	28.3	128
Bachelor’s degree	37.0	734	34.7	713	29.4	133
Master’s degree	13.4	267	13.2	270	25.4	115
Professional degree	1.2	23	1.7	34	6.4	29
Doctorate degree	3.0	59	2.1	43	7.5	34
Yearly income		1995		2053		451
< $10,000	20.2	403	13.0	266	13.1	59
$10,000–$29,999	24.0	479	27.7	568	14.0	63
$30,000–$49,999	19.6	391	23.2	476	14.2	64
$50,000–$69,999	15.3	306	15.2	312	14.4	65
> $69,999	16.3	326	19.5	401	63.4	286
Prefer not to say	4.1	90	1.5	30	7.8	35
Family status		1992		2053		447
Respondents with children	38.3	763	55.7	1144	53.0	237
Respondents with children (age 0–5)	14.9	296	13.2	270	9.4	42
Respondents with children (age 6–12)	14.6	290	15.7	322	10.5	47
Respondents with children (age 13–17)	9.2	183	13.6	280	9.8	44
Respondents with children (age > 17)	13.0	259	32.5	668	33.6	150

The percentage shares for ethnicity/race and gender do not add up to 100%, because respondents were able to tick multiple response options

### Procedures

Self-rated health was assessed using the Health-Related Quality of Life Core Module (CDC HRQoL-4). This module consists of four items about perceived general health, recent physical health, recent mental health, and recent activity limitations. The ordinal question about perceived general health did not meet the assumption of proportionality of odds required for ordered logistic regressions. Hence, we dichotomized the item into (1) fair or poor and (2) good, very good, or excellent. We measured current smoking and current vaping by number of cigarettes smoked per day and number of times vaping per day. These variables were dichotomized in the analysis (not smoking = 0, smoking = 1; not vaping = 0, vaping = 1) due to a high frequency of zero values (> 75%). Binge drinking was measured by the frequency of consuming five or more drinks on one occasion for males and four or more drinks on one occasion for females (within the last 3 months) [68]. We followed standard procedure and recoded these items into a unisex dichotomous variable (binge drinking less than monthly = 0, binge drinking monthly, weekly, or daily/almost daily = 1). BMI was calculated based on self-reported height and weight and dichotomized for analysis to reflect under or normal weight (BMI < 25 = 0) and overweight or obese (BMI ≥ 25 = 1) (item phrasing and response options for the health questions are reported in Table S5). Specifications on the nine demographic covariates (including item phrasing and response options) used in the regressions are presented in Table S6.

### Exploratory factor analysis

We opted to start our analysis with EFA, rather than CFA, because we considered EFA to be the most appropriate first step for a survey measure of this novelty. While the systematic review allowed us to distill core gender-related attitudes and behaviors, we were uncertain how many latent factors would emerge in the subsequent testing. Moreover, we were uncertain how several of the items would distribute across factors (e.g., the items for time-use and quality in family relationships) and we wanted to leave open the possibility that various items would cross-load onto factors other than their parent factor, potentially leading to fewer latent factors than initially expected. As described in the results section, this happened to be the case. In addition, since we had the opportunity and resources to collect three survey samples, a complementary approach combining EFA and CFA allowed us to benefit from advantages of each method.

The EFA was based on iterated principal axis factoring as the extraction method and Promax (oblique) rotation to allow for correlated factors. Exploratory factor analysis and statistical analyses were done in SPSS. In the EFA, we examined questions from all three gender categories in a common factor model with multiple factors. All respondents with missing data for relevant items were removed from sample 1 prior to the analysis. To allow for analysis of the largest possible sample, 10 items targeting caregivers and employees were recoded so that people not currently caring for someone in need or not currently employed (or employed in the past) were ascribed the value 1, which represents no strain due to caregiving or work (see Table S7).

We subjected the 44 questionnaire items to EFA in sample 1. Velicer’s minimum average partial test suggested a 7-factor structure (Table S8, screeplot, communalities, and unique variances are presented in Figure S2 and Table S9) [69]. The conceptual clarity of this solution also best resembled the thematic gender dimensions identified in the literature review. This solution retained 35 of the 44 items subjected to EFA and explained 51% of the variance in item scores. For purposes of interpretability, we excluded all items with loadings below 0.40.

Factor one in this solution includes six items addressing perceived discrimination. Factor two consists of seven items capturing daily time spent on work and work strain-related characteristics. Factor three encompasses four items concerning perceived strain and time-use related to caregiving. Factor four includes five items capturing competitive and risk-taking behavior. Factor five encompasses three items concerning perceived social support. Factor six includes six items capturing empathy and expressive behavior. Finally, factor seven includes four questions about independence. The distribution of items on factors was consistent across alternative factor rotation methods (Tables S10-S13).

### Confirmatory factor analysis

The CFA was carried out in SPSS AMOS Graphics 26 and based on maximum likelihood estimations. We allowed the factors to be correlated. The likelihood ratio test (also known as the *χ*^2^ test) is highly sensitive to even small departures of the data from exact fit, especially in large-N samples. Moreover, *χ*^2^ values increase with sample size and the number of variables in the model [70]. Therefore, we followed Cheung and Rensvold [71] and Yuan and Bentler [72] and determined global model fit and invariance based on the approximate-fit statistics. Specifically, we used the Tuckler Lewis Index (TLI), the root mean square error of approximation (RMSEA), and the standardized root mean square residual (SRMR), relying on conventional fit criteria [73]. The 35 items and 7 factors retained in the EFA were submitted to CFA in samples 1, 2, and 3. Following Gerbing and Hamilton [74], we used CFA to refine the EFA solution identified in sample 1. Next, we cross-validated the outcomes of this CFA in samples 2 and 3. Configural invariance was examined separately in samples 2 and 3. We used multiple-groups CFA to assess metric and scalar invariance across samples 2 and 3. We removed all observations with missing data for one or several of the 35 items subjected to CFA in sample 1, and for the 25 items subjected to CFA in samples 2 and 3. This slightly reduced sample 1 from *n* = 2051 to *n* = 2009, sample 2 from *n* = 2135 to *n* = 2054, and sample 3 from *n* = 489 to *n* = 449. One item concerning perceived gender discrimination in education had high rates of missing data in sample 3 (*N* > 100). Hence, we restricted the cross-validation in sample 3, and the multiple-groups CFA in samples 2 and 3, to the remaining 24 variables. To avoid a Heywood estimate on the Social support factor, we followed Chen et al. [75] and constrained the error variance of one item (socsupchores) to 0.001 in samples 2 and 3.

The initial 35-item solution based on the EFA did not perform satisfactorily with respect to global model fit in sample 1 (*χ*^2^ = 5897.38, df = 539, *p* = 0.00, TLI = 0.81, RMSEA = 0.07, SRMR = 0.07). To obtain acceptable model fit, we examined the factor loadings, removing all items with loadings ≤ 0.5. The “trimmed” solution, consisting of 25 items, exhibited good fit to the data (*χ*^2^ = 1362.53, df = 254, *p* = 0.00, TLI = 0.95, RMSEA = 0.05, SRMR = 0.04) (Table S14). The cross-validation of this final model in samples 2 and 3, with no equality constraints, also exhibited reasonable fit to the data, indicating configural invariance (sample 2: *χ*^2^ = 1440.7, df = 255, *p* = 0.00, TLI = 0.94, RMSEA = 0.05, SRMR = 0.04; sample 3: *χ*^2^ = 496.5, df = 232, *p* = 0.00, TLI = 0.93, RMSEA = 0.05, 3: SRMR = 0.05) (Table S15).

A more restricted multiple-groups CFA with factor loadings assumed to be equal across samples 2 and 3 also supported metric invariance (*χ*^2^ = 1899.849, df = 481, *p* = 0.00, TLI = 0.94, RMSEA = 0.03, SRMR = 0.04) (Table S16). Further restrictions with both factor loadings and intercepts assumed to be equal across samples 2 and 3 also indicated reasonable fit compared to prior models, supporting scalar invariance (*χ*^2^ = 2167.051, df = 505, *p* = 0.00, TLI = 0.93, RMSEA = 0.04, SRMR = 0.04) (Table S17). As a sensitivity check, we also ran the multiple groups CFA in samples 2 and 3 with the item on perceived gender discrimination in education included (sample 2 = 2054; sample 3 = 348) and obtained comparable model fit (metric invariance: *χ*^2^ = 2149.723, df = 528, *p* = 0.00, TLI = 0.93, RMSEA = 0.04, SRMR = 0.04; scalar invariance: *χ*^2^ = 2380.150, df = 553, *p* = 0.00, TLI = 0.93, RMSEA = 0.04, SRMR = 0.04) (Tables S18-S19).

### Reliability

The reliabilities of the factors implied by the final CFA solution were assessed in all samples using Raykov’s ρ. ρ was computed using James Gaskin’s “Validity master tool” [76], and following conventional criteria [77], we considered values > 0.60 desirable.

## Results

Table 3 reports the factor loadings, Raykov’s ρ, and item scoring for the trimmed CFA model in samples 1, 2, and 3, with seven variables. The 7 factors listed above represent our final gender-related variables. Our analyses yielded low to moderate inter-factor correlations (Table S20), which is not unusual in multidimensional gender measures [26, 78, 79]. We calculated mean-item subscale scores for each factor and used these as predictors in the regressions presented below. For subscales including continuous variables, mean-item scores were calculated based on standardized variables (z-scores). All variables are scored from lower to higher levels of the given constructs.

**Table 3:** Factor loadings for the trimmed CFA models in Samples 1, 2 and 3 and Raykov’s ρ for each factor

	Item scoring	Sample 1 (*n* = 2009)	Sample 2 (*n* = 2054)	Sample 3 (*n* = 449)
Caregiver strain		*ρ* = 0.92	*ρ* = 0.92	*ρ* = 0.91
In the past year, how often did you feel physically exhausted because of your caretaking responsibilities?	5-point scale (Never = 1, 5 = Always)	0.941	0.947	0.933
In the past year, how often did you feel emotionally exhausted because of your caretaking responsibilities?	5-point scale (Never = 1, 5 = Always)	0.953	0.971	0.942
In the past year, how often have your caretaking responsibilities caused you to worry about the future?	5-point scale (Never = 1, 5 = Always)	0.910	0.892	0.905
On average, how many hours per weekday do you spend on taking care of someone in need (caring for children, elders, partners in need, etc.)?	Open-ended numerical estimate (1-24)	0.643	0.620	0.526
Work strain		*ρ* = 0.87	*ρ* = 0.86	*ρ* = 0.91
How often does your job require working fast?	5-point scale (Never = 1, 5 = Always)	0.864	0.850	0.905
How often does your job involve repetitive tasks?	5-point scale (Never = 1, 5 = Always)	0.821	0.789	0.865
How often do you feel emotionally exhausted from your work activities?	5-point scale (Never = 1, 5 = Always)	0.792	0.791	0.872
How often do you feel physically exhausted from your work activities?	5-point scale (Never = 1, 5 = Always)	0.775	0.760	0.825
On average, how many hours per weekday do you spend on the following: Work (paid work, studying, internships, etc.)?	Open-ended numerical estimate (1-24)	0.520	0.492	0.619
Independence		*ρ* = 0.75	*ρ* = 0.75	*ρ* = 0.67
How important is it for you to solve your problems on your own?	5-point scale (Not at all important = 1, Extremely important = 5)	0.793	0.837	0.768
How important is it for you to be independent?	5-point scale (Not at all important = 1, Extremely important = 5)	0.758	0.76	0.656
Risk-taking		*ρ* = 0.77	*ρ* = 0.76	*ρ* = 0.67
In general, how prepared are you to take risks?	5-point scale (Not at all prepared = 1, Completely prepared = 5)	0.837	0.842	0.820
How prepared are you to take risks when making financial decisions?	5-point scale (Not at all prepared = 1, Completely prepared = 5)	0.672	0.679	0.575
How prepared are you to take risks when it comes to recreational activities?	5-point scale (Not at all prepared = 1, Completely prepared = 5)	0.649	0.620	0.501
Emotional intelligence		*ρ* = 0.65	*ρ* = 0.65	*ρ* = 0.65
How often do friends talk to you about their problems?	5-point scale (Never = 1, Always = 5)	0.513	0.526	0.521
How often do you talk to your friends about your problems?	5-point scale (Never = 1, Always = 5)	0.745	0.760	0.775
How easy is it for you to express what you are feeling to others?	5-point scale (Not at all easy = 1, Extremely easy = 5)	0.593	0.566	0.541
Social support		*ρ* = 0.71	*ρ* = 0.77	*ρ* = 0.74
In the past year, how often did you have someone to show you love and affection?	5-point scale (Never = 1, 5 = Always)	0.896	0.973	0.909
In the past year, how often did you have someone to help you with daily chores?	5-point scale (Never = 1, 5 = Always)	0.610	0.582	0.607
Discrimination		*ρ* = 0.86	*ρ* = 0.82	*ρ* = 0.74
Because of your gender, how often have you felt discriminated against?	5-point scale (Never = 1, 5 = Always)	0.850	0.840	0.860
Because of your gender, how often have you felt discriminated against when getting hired?	5-point scale (Never = 1, 5 = Always)	0.707	0.726	0.537
Because of your gender, how often have you felt discriminated against when at school?	5-point scale (Never = 1, 5 = Always)	0.776	0.746	–
Because of your gender, how often have you felt discriminated against when receiving medical care?	5-point scale (Never = 1, 5 = Always)	0.727	0.693	0.608
Because of your gender, how often have you felt discriminated against in public settings?	5-point scale (Never = 1, 5 = Always)	0.846	0.860	0.845
Because of your gender, how often have you felt discriminated against in your family?	5-point scale (Never = 1, 5 = Always)	0.648	0.625	0.577

**Fig. 1:**
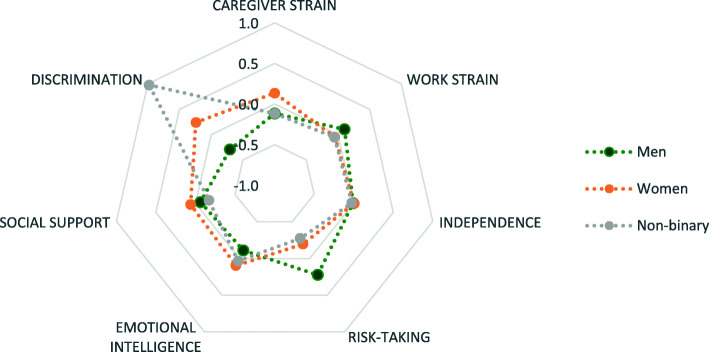
Gender-related variables capturing specific behaviors and attitudes. The figure displays the z-scores for the seven gender-related variables for respondents seeing themselves as men (green), women (orange) and gender fluid/Non-binary (grey) in sample 1 (*N* = 1893)

Figure 1 displays the z-scores (averaged by group) for the 7 gender-related variables for respondents seeing themselves as men, women, and gender fluid/non-binary in sample one. The figure demonstrates the advantage of capturing specific gender-related behaviors and attitudes through multiple variables.

### Associations with self-rated health and health-risk behaviors

Existing research shows notable sex differences in health-related quality of life, obesity, and risky health behaviors, such as smoking and heavy drinking [80–82]. Here, we examine the relevance of our gender-related variables in predicting self-related health and health-risk behaviors.

Associations between our 7 gender-related variables, sex assigned at birth, self-reported gender identity and physical health, mental health, and activity limitations due to poor physical or mental health were analyzed in samples 1 and 2 using negative binomial regressions, with birth year, personal income, education level, race, and ethnicity as covariates. Only associations that are consistent across samples 1 and 2 are reported here. Measures of reported birth sex and gender identity were highly correlated. Therefore, we ran all models twice: once including birth sex and once including gender identity. Here, we report the outcomes of the models including birth sex as a key predictor (see Tables S21-S23 for specifications on the regression models including gender identity).

As displayed in Table 4, caregiver strain and discrimination were associated with lower physical health, mental health, and activity levels in both samples, whereas social support was associated with higher mental health and activity levels in both samples. The results for the remaining associations were inconclusive in one or both samples.

**Table 4:** Adjusted incidence rate ratios and 95% confidence intervals of associations with health-related measures in negative binomial regressions

	Physical health^a^		Mental health^b^		Activity limitations^c^	
	Sample 1 (*n* = 1874)	Sample 2 (*n* = 2005)	Sample 1 (*n* = 1876)	Sample 2 (*n* = 2004)	Sample 1 (*n* = 1872)	Sample 2 (*n* = 2004)
Caregiver strain	1.20 (1.12:1.29)	1.11 (1.04:1.18)	1.21 (1.14:1.27)	1.20 (1.14:1.27)	1.13 (1.04:1.21)	1.11 (1.03:1.19)
Work strain	0.96 ( 0.87: 1.05)	1.02 (0.94:1.11)	1.03 (0.96:1.11)	1.12 (1.04:1.21)	0.88 (0.80:.96)	0.99 (0.90:1.08)
Independence	0.93 (0.85:1.01)	0.84 (0.75:.95)	0.97 (0.91:.1.04)	0.83 (0.74:.94)	1.02 (0.94:1.12)	0.87 (0.77:1.00)
Risk-taking	0.95 (0.87:1.04)	1.00 (0.92:1.08)	0.86 (0.80:.92)	0.99 (0.91:1.08)	0.93 (0.85:1.01)	1.02 (0.94:1.12)
Emotional intelligence	0.93 (0.84:1.03)	0.93 (0.85:1.01)	0.97 (0.89:1.05)	0.86 (0.78:.93)	0.98 (0.88:1.10)	0.92 (0.84:1.02)
Social support	0.95 (0.89:1.02)	0.94 (0.89:1.00)	0.86 (0.81:.90)	0.81 (0.76:.86)	0.92 (0.86:.99)	0.87 (0.81:.94)
Discrimination	1.46 (1.34:1.59)	1.43 (1.30:1.57)	1.44 (1.34:1.53)	1.35 (1.24:1.48)	1.76 (1.61:1.93)	1.56 (1.40:1.73)
Sex (male=0)	0.94 (0.81:1.10)	0.93 (0.80:1.08)	0.94 (0.83:1.06)	1.21 (1.05:1.40)	0.88 (0.74:1.04)	1.11 (0.94:1.31)

Incidence rate ratios are adjusted for year of birth, ethnicity, race, education, and personal income. See Tables S29-S31 for model specifications

^a^Number of days with poor physical health during the last 30 days

^b^Number of days with poor mental health during the last 30 days

^c^Number of days with activity limitations due to poor physical and mental health during the last 30 days

Associations between our gender-related variables, sex assigned at birth, self-reported gender identity and general health status, smoking, vaping, binge drinking, and BMI were analyzed using logistic regressions in samples 1 and 2 (Table 5, adjusted for year of birth, personal income, education level, ethnicity, and race), and we ran separate models with sex and gender identity (see Tables S24-S28 for specifications on the regression models including gender identity). In both samples, caregiver strain, discrimination, and male birth sex were associated with fair or poor self-rated health, while risk-taking and social support predicted good, very good, or excellent self-rated health. Further, caregiver strain and work strain were associated with smoking, while discrimination was associated with vaping and higher levels of risk-taking was associated with binge drinking. Caregiver strain, low levels of risk-taking, discrimination, and male birth sex were associated with overweight. The results for the remaining associations were inconclusive in one or both samples.

**Table 5:** Adjusted odds ratios and 95% CIs of associations with health-related measures in logistic regressions

	Health status^a^		Smoking^b^		Vaping^c^		Binge drinking^d^		BMI^e^	
	Sample 1 (*n* = 1884)	Sample 2 (*n* = 2010)	Sample 1 (*n* = 1883)	Sample 2 (*n* = 2009)	Sample 1 (*n* = 1882)	Sample 2 (*n* = 2009)	Sample 1 (*n* = 1880)	Sample 2 (*n* = 2007)	Sample 1 (*n* = 1858)	Sample 2 (*n* = 1993)
Caregiver strain	1.28 (1.11:1.47)	1.18 (1.03:1.34)	1.42 (1.24:1.62)	1.18 (1.05:1.33)	1.06 (0.90:1.26)	1.10 (0.93:1.29)	0.92 (0.81:1.06)	0.91 (0.80:1.04)	1.18 (1.05:1.32)	1.27 (1.13:1.41)
Work strain	0.88 (0.74:1.06)	0.98 (0.83:1.17)	1.37 (1.15:1.64)	1.26 (1.08.:1.46)	1.23 (0.97:1.55)	1.29 (1.05:1.59)	1.28 (1.08:1.51)	1.03 (0.88:1.21)	1.04 (0.91:1.19)	1.17 (1.02:1.33)
Independence	0.91 (0.77:1.07)	0.71 (0.56:0.89)	1.22 (1.03:1.46)	1.07 (0.85:1.35)	1.17 (0.95:1.45)	1.05 (0.76:1.44)	1.08 (0.93:1.25)	1.19 (0.94:1.49)	0.99 (0.88:1.12)	1.05 (0.87:1.26)
Risk-taking	0.57 (0.48:0.68)	0.69 (0.58:0.81)	1.45 (1.21:1.72)	1.05 (0.91:1.21)	1.20 (0.98:1.48)	1.00 (0.80:1.25)	1.53 (1.31:1.78)	1.25 (1.07:1.45)	0.82 (0.72:.93)	0.85 (0.75:0.97)
Emotional intelligence	0.91 (0.76:1.10)	0.90 (0.75:1.07)	1.46 (1.22:1.75)	1.12 (0.96:1.30)	1.39 (1.12:1.71)	1.05 (0.84:1.31)	1.18 (1.00:1.40)	1.04 (0.88:1.21)	0.99 (0.86:1.14)	1.03 (0.90:1.18)
Social support	0.83 (0.73:0.95)	0.78 (0.69:0.88)	0.98 (0.86:1.12)	0.97 (0.87:1.09)	0.98 (0.84:1.14)	0.88 (0.76:1.04)	1.01 (0.90:1.13)	0.87 (0.78:.97)	1.00 (0.91:1.10)	1.07 (0.97:.1.18)
Discrimination	1.43 (1.18:1.73)	1.55 (1.29:1.87)	1.09 (.90:1.31)	1.16 (0.97:1.38)	1.40 (1.15:1.70)	1.46 (1.17:1.81)	1.18 (1.00:1.39)	1.11 (0.92:1.32)	1.23 (1.06:1.42)	1.26 (1.08:1.48)
Sex (male = 0)	0.60 (0.44:0.82)	0.65 (0.48:0.87)	0.66 (0.48:0.89)	0.89 (0.69:1.16)	0.45 (0.31:0.64)	0.76 (0.52:1.11)	0.55 (0.42:0.71)	0.77 (0.59:.1.01)	0.76 (0.60:0.95)	0.58 (0.46:0.73)

Odds ratios are adjusted for year of birth, ethnicity, race, education, and personal income. Tables S32-S36 for model specifications

^a^Good, very good, excellent = 0, fair, and poor = 1

^b^Not smoking = 0, smoking = 1

^c^Not vaping = 0, vaping = 1

^d^Less than monthly = 0, monthly, weekly, and daily = 1

^e^Under or normal weight (BMI < 25) = 0, overweight or obese (BMI ≥ 25) = 1

**Fig. 2:**
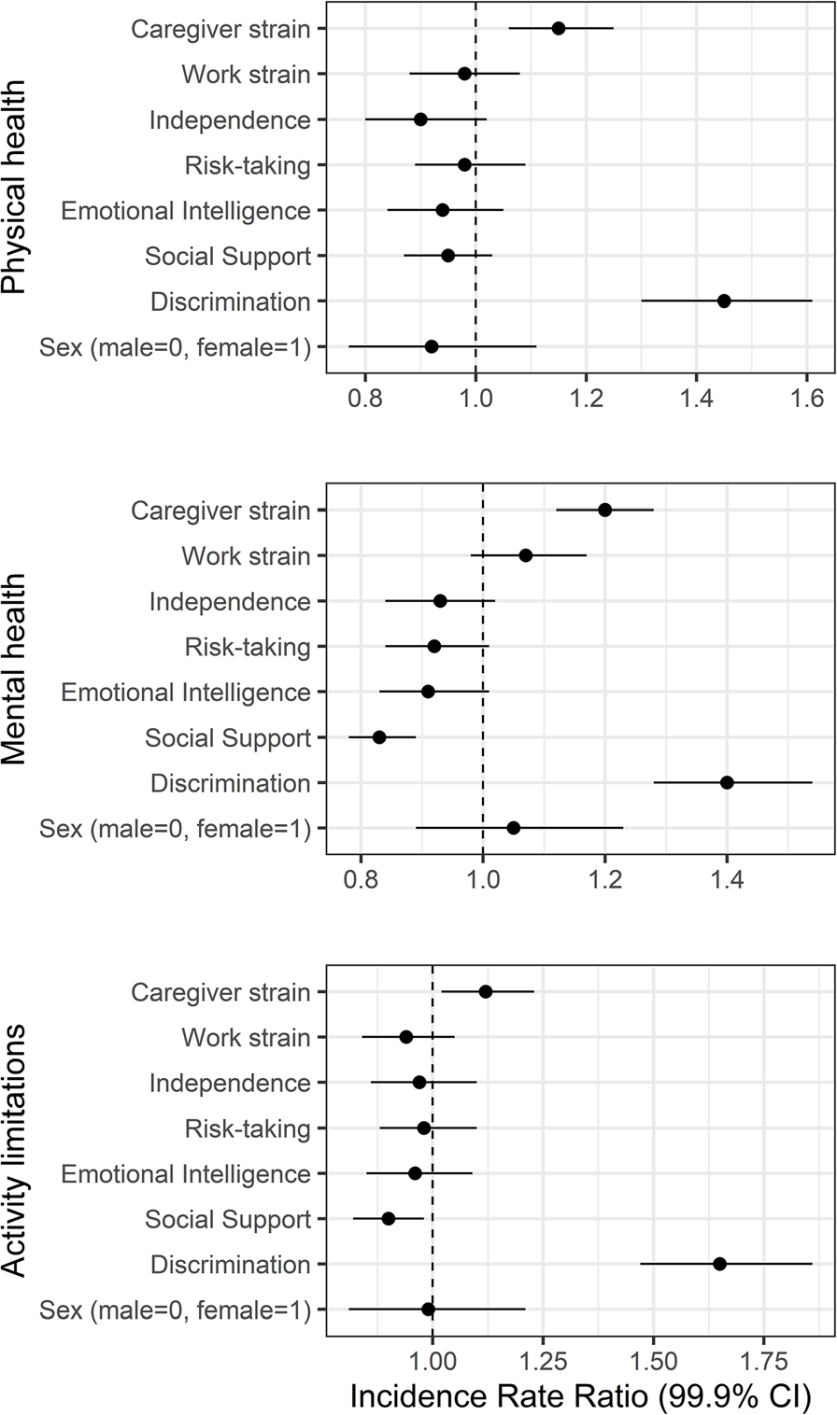
Adjusted incidence rate ratios of associations with recent physical health, mental health, and activity limitations. This figure displays the outcomes of the negative binomial regressions predicting health outcomes in the combined sample (sample 1 + sample 2) (Physical health, *N* = 3879; mental health, *N* = 3880; activity limitations, *N* = 3876). Error bars represent 99.9% confidence intervals. See Tables S37-S39 for model specifications

**Fig. 3:**
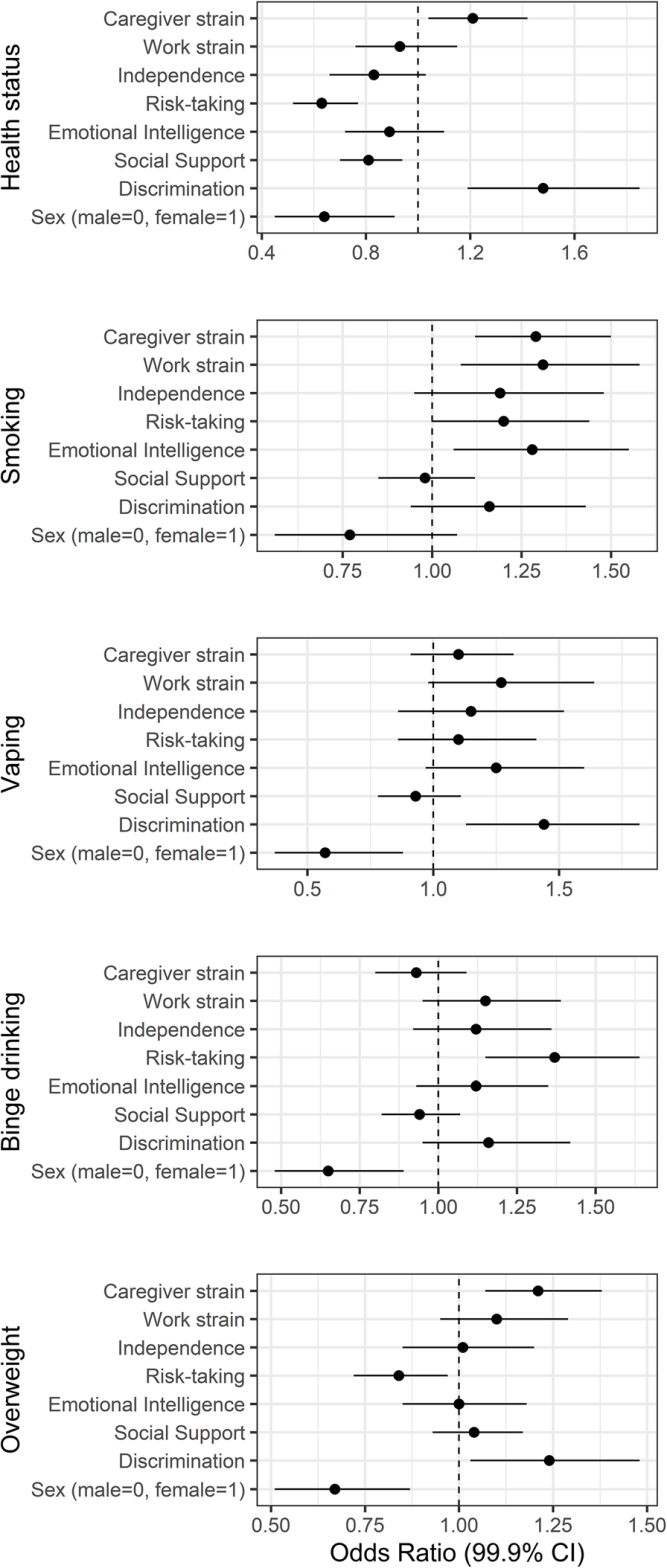
Adjusted odds ratios of associations with health status, smoking, vaping, binge drinking, and BMI. This figure displays the outcomes of the binary logistic regressions predicting health outcomes in the combined sample (sample 1 + sample 2) (health status, *N* = 3,894; smoking, *N* = 3,892 vaping, *N* = 3,891; binge drinking, *N* = 3,887; BMI, *N* = 3,851. Error bars represent 99.9% confidence intervals. See Tables S40-S44 for model specifications

Combining the data from samples 1 and 2 (and adjusting for sample in the regression models), all associations with self-related health and health-risk behaviors reported above persisted at the 99.9% confidence level (Figs. 2 and 3), as did the following associations: emotional intelligence with smoking and male birth sex with vaping and binge drinking.

## Discussion

Following a comprehensive review of gender measures from 1975 to 2015, we have applied a rigorous process to identify key aspects of gender for the purpose of developing a new gender assessment tool for use in clinical and population research, including large-scale health surveys involving diverse Western populations. Through exploratory and confirmatory factor analyses, we reduced the original 44 survey items to 25 (Table S45) and the 11 original constructs to 7 gender-related variables: caregiver strain, work strain, independence, risk-taking, emotional intelligence, social support, and discrimination.

Each variable seeks to capture an important aspect of gender within the populations studied. Each variable measures an individual participant’s self-reported behavior or attitude for that characteristic and is designed to be scored individually, as a distinct human behavior or attribute. Behaviors are not coded “masculine” or “feminine,” and we recommend against consolidating the variable scores into these unipolar or bipolar indices. Studies that reduce gender-related variables to “femininity” or “masculinity” scores give little guidance for behavioral interventions. For example, if caregiver strain is found to be associated with higher risk of recurrence or death in patients with ACS, it should be reported as such. Subsuming gender-related factors into masculine or feminine indices will reduce, rather than improve, the precision and applicability of survey-based measures of health.

Moreover, the regression analyses (Figs. 2 and 3) suggest that the gender-related variables pertaining to norms (caregiver strain and work strain) and relations (discrimination and social support) have stronger correlations with self-rated health measures than the variables pertaining to gender-related traits (with risk-taking as an important exception). This finding aligns with extant research suggesting that institutional and interpersonal aspects of gender may be more important than individual traits and characteristics in shaping health and disease processes [17].

Clinicians and public health researchers may employ the Stanford GVHR to gain a fuller understanding of gender differences in health outcomes than can be captured by simply asking people to self-identify their sex or gender. It might be used, for example, in the treatment of chronic pain or osteoporosis research, each of which has robust gender components [83]. Specifically, we recommend that researchers start by measuring all 7 variables alongside sex assigned at birth, self-reported gender identity, and other relevant factors, such as sexual orientation, ethnicity, age, income, and education, to specify which characteristics, traits, and behaviors may be predictive of the health issue in focus. In subsequent studies and patient-based outcome measures, the focus may be restricted to a subset of variables of documented relevance to a specific disease or health condition [84]. Our gender-related variables are developed to capture how gender norms, gender-related traits, and gender relations intersect to shape people’s health and disease (and vice versa). Researchers using our instrument are encouraged to be aware of broader institutional and cultural contexts that may influence patients’ gender roles and identities and health outcomes.

The Stanford GVHR is specific to the survey cohorts, time, place, and culture in which it was developed and tested. Because gender norms, traits, and relations vary across and within cultures and change over time [85], we recommend that variables be updated with each generation or even more frequently. Obvious limitations are that our variables were developed from English-language literature and validated in nonprobability samples, recruited online and through a research registry in US populations, and the age composition of our samples (median ages range from 36 to 50 years) does not represent typical patient populations encountered in clinical practice. We strongly encourage more research to test the validity of the gender-related variables within and across age groups and cultures, as well as across a wide range of global settings.

Any association of a gender-related variable with a health outcome does not infer causality. Our assessment was cross-sectional. Confounding or even reverse causality (health phenotypes affecting gender-related behaviors and attitudes) should be considered. Another limitation is the small number of items per construct which might limit generalizability; at the same time, a smaller number of items likely increases the usefulness of the questionnaire to practitioners and researchers. An avenue for further research is the expansion the number of items for each construct, especially emotional intelligence, which had less than ideal reliability across all three samples. In addition, a few of the initial gender-related constructs that were not retained in the exploratory factor analysis, such as competition and quality of family relationships, may have been deselected due to insufficient items in the study’s initial pool of attitudes and behaviors and should be reconsidered in future research.

### Perspectives and significance

This project represents an approach toward developing more comprehensive and precise survey-based measures of gender in relation to health. In the future, the proposed list of variables to measure other gender-related factors could be expanded to place more emphasis on gender relations, e.g., by integrating factors such as decision-making power (including over household resources and health expenditures) and the distribution of domestic labor in families and among both same- and different-sex cohabiting or romantic partners. Indeed, many of the measures that have informed our work may be outdated (noting that some date back to the 1970s and 1980s). It may be desirable to complement our proposed list of variables with more “timely” variables that better represent how specific patients and persons conceive of gender in 2020. It would also be interesting to explore associations between our gender-related variables and other health-related aspects such as health literacy, health-seeking behavior, and provider-patient interactions.

## Conclusion

Our questionnaire is designed to shed light on how specific gender-related behaviors and attitudes contribute to health and disease processes, irrespective of—or in addition to—biological sex and self-reported gender identity. Use of these gender-related variables in experimental studies, such as clinical trials, may also help us understand if gender factors play an important role as treatment effect modifiers and would thus need to be further considered in treatment decision-making.

## Supplementary Information

**Additional file 1: Fig. S1.** Flowchart of article inclusion and exclusion in the literature search. **Fig. S2.** Screeplot of the factor analysis reported in Table S8. **Table S1.** Item phrasing and descriptive statistics for the 44 potentially relevant gender-related items. **Table S2.** Response options for all 44 items included in the exploratory factor analyses. **Table S3.** Rank of gender characteristics based on occurrences (>2). **Table S4.** Search-terms for meta-analyses of existing scales measuring each gender variable. **Table S5.** Health-related items and response options. **Table S6.** Demographic items and response options. **Table S7.** Recoding of ten variables to allow for the largest possible sample in the EFA. **Table S8.** Exploratory Factor Analysis (Full factor model). **Table S9.** Communalities and unique variances for exploratory factor analysis presented in Table S8. **Table S10.** Exploratory Factor Analysis (Full factor model), Oblimin rotation. **Table S11.** Exploratory Factor Analysis (Full factor model), Varimax rotation. **Table S12.** Exploratory Factor Analysis (Full factor m odel), Equamax rotation. **Table S13.** Exploratory Factor Analysis (Full factor model), Quartimax rotation. **Table S14.** Factor loadings for CFA Models 1 and 2 in sample 1. **Table S15.** Factor loadings for CFA Samples 2 and 3 (Configural invariance). **Table S16.** Factor loadings for CFA Samples 2 and 3 (Metric invariance, 24 items). **Table S17.** Factor loadings for final CFA in samples 2 and 3 (Scalar invariance, 24 items). **Table S18.** Factor loadings for final CFA in samples 2 and 3 (Metric invariance, 25 items). **Table S19.** Factor loadings for final CFA samples 2 and 3 (Scalar invariance, 25 items). **Table S20.** Correlations between the factors in samples 1, 2 and 3. **Table S21.** Negative binomial regression predicting number of days with poor physical health (during past 30 days) (with gender identity as covariate). **Table S22.** Negative Binomial regression predicting number of days with poor mental health (during past 30 days) (with gender identity as covariate). **Table S23.** Negative binomial regression predicting number of days where poor mental or physical health prevented the respondent from doing usual activities (during past 30 days) (with gender identity as covariate). **Table S24.** Logistic regression predicting general health status (excellent, very good, good= 0, fair, poor= 1) (with gender identity as covariate). **Table S25.** Logistic regression predicting vaping (not vaping=0, vaping=1) (with gender identity as covariate). **Table S26.** Logistic regression predicting smoking (not smoking=0, smoking=1) (with gender identity as covariate). **Table S27.** Logistic regression predicting binge drinking (less than monthly=0, monthly, weekly, and daily=1) (with gender identity as covariate). **Table S28.** Logistic regression predicting overweight (BMI<25=0, BMI≥25 =1) (with gender identity as covariate). **Table S29.** Negative binomial regression predicting number of days with poor physical health (during past 30 days) (with sex as covariate). **Table S30.** Negative Binomial regression predicting number of days with poor mental health (during past 30 days) (with sex as covariate). **Table S31.** Negative binomial regression predicting number of days where poor mental or physical health prevented the respondent from doing usual activities (during past 30 days) (with sex as covariate). **Table S32.** Logistic regression predicting general health status (excellent, very good, good= 0, fair, poor= 1) (with sex as covariate). **Table S33.** Logistic regression predicting smoking (not smoking=0, smoking=1) (with sex as covariate). **Table S34.** Logistic regression predicting vaping (not vaping=0, vaping=1) (with sex as covariate). **Table S35.** Logistic regression predicting binge drinking (less than monthly=0, monthly, weekly, and daily=1) (with sex as covariate). **Table S36.** Logistic regression predicting Overweight (BMI<25=0, BMI≥25 =1) (with sex as covariate). **Table S37.** Negative binomial regression predicting number of days with poor physical health (during past 30 days) (combined samples). **Table S38.** Negative binomial regression predicting number of days with mental health (during past 30 days) (combined samples). **Table S39.** Negative binomial regression predicting number of days where poor mental or physical health prevented the respondent from doing usual activities (during past 30 days) (combined samples). **Table S40.** Logistic regression predicting general health status (excellent, very good, good= 0, fair, poor= 1) (combined samples). **Table S41.** Logistic regression predicting smoking (not smoking=0, smoking=1) (combined samples). **Table S42.** Logistic regression predicting vaping (not vaping=0, vaping=1) (combined samples). **Table S43.** Logistic regression predicting binge drinking (less than monthly=0, monthly, weekly, and daily=1) (combined samples). **Table S44.** Logistic regression predicting Overweight (BMI<25=0, BMI≥25 =1) (combined samples). **Table S45.** Final 25 survey items.

## Data Availability

Supplementary Information (SI) Text is included with this submission. Data and code needed to evaluate the conclusions are available here: https://osf.io/7yje9/.
