# Supplementary material for: Gender-related variables for health research
Source: Biol Sex Differ. 2021 Feb 22;12:23. doi: 10.1186/s13293-021-00366-3 (PMC7898259; doi:10.1186/s13293-021-00366-3)
Supplement: Supplementary file 1 — Additional file 1: Fig. S1. Flowchart of article inclusion and exclusion in the literature search. Fig. S2. Screeplot of the factor analysis reported in Table S8. Table S1. Item phrasing and descriptive statistics for the 44 potentially relevant gender-related items. Table S2. Response options for all 44 items included in the exploratory factor analyses. Table S3. Rank of gender characteristics based on occurrences (>2). Table S4. Search-terms for meta-analyses of existing scales measuring each gender variable. Table S5. Health-related items and response options. Table S6. Demographic items and response options. Table S7. Recoding of ten variables to allow for the largest possible sample in the EFA. Table S8. Exploratory Factor Analysis (Full factor model). Table S9. Communalities and unique variances for exploratory factor analysis presented in Table S8. Table S10. Exploratory Factor Analysis (Full factor model), Oblimin rotation. Table S11. Exploratory Factor Analysis (Full factor model), Varimax rotation. Table S12. Exploratory Factor Analysis (Full factor m odel), Equamax rotation. Table S13. Exploratory Factor Analysis (Full factor model), Quartimax rotation. Table S14. Factor loadings for CFA Models 1 and 2 in sample 1. Table S15. Factor loadings for CFA Samples 2 and 3 (Configural invariance). Table S16. Factor loadings for CFA Samples 2 and 3 (Metric invariance, 24 items). Table S17. Factor loadings for final CFA in samples 2 and 3 (Scalar invariance, 24 items). Table S18. Factor loadings for final CFA in samples 2 and 3 (Metric invariance, 25 items). Table S19. Factor loadings for final CFA samples 2 and 3 (Scalar invariance, 25 items). Table S20. Correlations between the factors in samples 1, 2 and 3. Table S21. Negative binomial regression predicting number of days with poor physical health (during past 30 days) (with gender identity as covariate). Table S22. Negative Binomial regression predicting number of days with poor mental health (during past 30 da [file 13293_2021_366_MOESM1_ESM.pdf]

Gender-Related Variables for Health Research

Supplementary Information (SI) Text

Methods: Documentation of Literature survey

Here we document the systematic literature search that guided our development of the gender variables. The description of the literature survey is divided into five steps. Step 1 specifies the test-specific literature searches in PsycInfo, PsycNet, and PubMed with respect to database specific search limitations, search terms and selection criteria. Step 2 lists all of the gender measures derived from the articles identified through the searches in PsycInfo, PsycNet and PubMed. In Step 3, we sort the identified gender measures into three overarching categories for analyzing gender (norms, identity and relations), and use citation frequencies to determine the prevalence and use of each scale in the literature. In Step 4, we identify the core traits and characteristics of relevance to each of the three categories of gender-related traits, gender norms, and gender relations. Finally, Step 5 documents the search strategy used to identify additional construct specific measures of relevance to our gender variables.

Step 1: Test-specific literature search in PsycINFO, PsycNET, and PubMed

This section specifies our search strategy. We carried out systematic searches in PsychINFO, PsycNET, and PubMED. We restricted the time span to the period from January 1975 through 2015.

Search limitations. We used the following database-specific search limitations:

> PsycINFO: Document type: Research literature in peer reviewed scholarly journals; Language: English; Population: Human; Classification: "Personality Scales & Inventories"; "Tests & Testing".
>
> PsycNet: Age-group: Adulthood (18 years or older); Document type: Abstract collection, Peer-reviewed articles; PsycNET Classifiers: "Tests and testing"; "Personality Scales & Inventories"; "Clinical Psychological Testing"; "Health Psychology Testing"; "Educational measurement"; "Occupational and employment testing"; "Consumer opinion and attitudes".
>
> PUBMED: Ages: all ages; Species: Humans; Language: English; Article types: all.
>
> 1. The search was narrowed to the following gender/sex related MeSH-terms: "gender identity"; "sex"; "sexism"; "interpersonal relations"; "female", "male". We excluded: "sexual and gender disorders"; "gender dysphoria"; "sex reassignment surgery"; "sex reassignment procedures.
> 2. We narrowed our search to the following "test-related" MeSH terms: "psychological tests"; "neuropsychological tests"; "personality tests"; "psychometrics"; "pain measurement"; 'behavior rating scale"; "thematic

apperception test"; "test anxiety scale"; "personal construct theory"; "Wechsler scales"; "personality assessment".

Search strings. We used the following search strings in the three databases:

<u>PsycINFO:</u> ("Gender Identity" OR "Gender Gap" OR "Gender Equality" OR "Sex Role Attitudes" OR "Sex Roles")

<u>PsycNet</u>: ("Gender" OR "Sex") NOT ("sexual")

<u>PubMed:</u> (Psychological tests[mh] OR Neuropsychological tests[mh] OR Personality tests[mh] OR Psychometrics[mh] OR Pain measurement[mh] OR Behavior rating scale[mh] OR Thematic Apperception Test[mh] OR Test anxiety scale[mh] OR Personal Construct Theory[mh] OR Wechsler Scales[mh] OR Personality Assessment[mh]) AND (Sex[mh] OR Gender identity[mh] OR Interpersonal relations[mh] OR Sexism[mh] OR women[mh] OR men[mh] or female [mh] OR male [mh]) AND (scale*[ti] OR measure*[ti] OR compos*[ti] OR inventor*[ti] OR index*[ti] OR indic*[ti] OR score*[ti] OR psychomet*[ti] OR instrument*[ti] OR batter*[ti] OR assess*[ti] OR test*[ti] OR rating*[ti]) AND (Gender*[ti] OR sex[ti] OR sexes[ti] OR mascu*[ti] OR femin*[ti] OR male[ti] OR males[ti] OR female*[ti] OR man[ti] OR woman[ti] OR men[ti]] OR women[ti] OR androgy*[ti] OR boy[ti] OR girl[ti] OR boys[ti] OR girls[ti]).

The literature on gender- and sex-related scales is extremely extensive and for reasons of feasibility, it will not be possible to capture everything through a broad search in PubMed.
An important caveat related to our selected search string in PubMed is the emphasis on gender/ sex related search terms in abstract titles. To provide a concrete example: Our search string may not capture potentially relevant scales that do not include the following terms in their titles: (Gender*[ti] OR sex[ti] OR sexes[ti] OR mascu*[ti] OR femin*[ti] OR male[ti] OR males[ti] OR female*[ti] OR man[ti] OR woman[ti] OR men[ti]] OR women[ti] OR androgy*[ti] OR boy[ti] OR girl[ti] OR boys[ti] OR girls[ti]). However, for influential scales we will likely capture several follow up studies adapting or testing the validity of these scales. Test of coverage is captured in a later step.

The search strings presented above returned 519 studies in PsycINFO, 432 studies in PsycNET and 2030 studies in PubMed (Fig. S1).

Selection criteria. We read through abstract and titles and used the following selection criteria to identify relevant publications:

All studies with scales or tests measuring aspects of gender are eligible, regardless of purpose of use in article (+); articles using non-gender related measures to illuminate gender differences on a given trait or topic (-); articles focusing on children or adolescents (17 years old or younger) (-); articles capturing scales

measuring issues of gender norms or relations (e.g. child-rearing, romantic relationships, division of domestic labor) (+); articles capturing scales concerning sexuality, sexual health, sexual violence, domestic violence and issues of gender- and sex development in childhood and adolescence (-) and body image (-).

Eligible publications. Using these, we identified 405 eligible publications: 315 in PsycINFO, 65 in PsychNET and 89 in PubMed. Of these, 64 publications were dublicates or triplicates (Fig. S1)

Step 2: Identifying measures of gender

As a second step in the review process, we searched through all of the 405 eligible references to identify relevant gender measures employed in each paper. We derived 127 unique gender measures through this approach. Four additional gender measures were identified through literature reviews published in books.

Step 3: Sorting and determining the impact of gender measures

As a third step in the review process, we sorted the 131 gender measures into three overarching categories for analyzing gender (norms, identity and relations). Given the large number of gender measures, we decided to use citation frequencies to determine the prevalence and use of each measure in the literature. All measures receiving at least 20 citations in Google Scholar within the last ten years were selected for further investigation, of which 74 scales met the criteria. All articles published from 2006-2015 were retained for further investigation. All articles published in 2005 or earlier, with less than 20 citations in Google Scholar were assorted into the “Ineligible Scales” category. This category also comprises measures that we found irrelevant after reading through abstract and full text. The scales assorted into each category (norms, identity and relations) are ranked based on their citation impact. Ineligible scales are listed at the bottom of this section.

Gender norms (N=3)

Coparenting and Family Rating System
Original scale: McHale, J. P., Kuersten-Hogan, R., & Lauretti, A. (2000). Evaluating coparenting and family-level dynamics during infancy and early childhood: The Coparenting and Family Rating System. *Family observational coding systems: Resources for systemic research*, 151-170.
Web of Science (2006->): Missing
Google scholar (2006->): 57

Parental Child-rearing Behaviour Scale
Original scale: Meunier, J. C., & Roskam, I. (2007). Psychometric properties of a parental childrearing behavior scale for French-speaking parents, children, and adolescents. *European Journal of Psychological Assessment*, *23*(2), 113-124.
Web of Science (2006->): 17 (full database), 3 (health-related subject categories)
Google scholar (2006->): 29

The Orientation towards Domestic Labor

Original scale: Hawkins, A. J., Marshall, C. M., & Allen, S. M. (1998). The Orientation Toward Domestic Labor Questionnaire: Exploring dual-earner wives' sense of fairness about family work. *Journal of Family Psychology*, *12*(2), 244.
Web of Science (2006->): 7 (full database)
Google scholar (2006->): 28

Gender-related traits (N=31)

Bem Sex Role Inventory
Original scale: Bem, S. L. (1974). The measurement of psychological androgyny. *Journal of consulting and clinical psychology*, *42*, 155-162
Web of Science (2006->): 802 (full database), 100 (health research)
Google scholar (2006->): 3670

Minnesota Multiphasic Personality Inventory (broader, including masc. and fem.)
Original scale: Hathaway, S. R., & McKinley, J. C. (1951). Minnesota Multiphasic Personality Inventory; manual (Revised).
Web of Science (2006->): Missing
Google scholar (2006->): 937

Conformity to Masculine Norms Inventory
Original scale: Mahalik, J. R., Locke, B. D., Ludlow, L. H., Diemer, M. A., Scott, R. P., Gottfried, M., & Freitas, G. (2003). Development of the Conformity to Masculine Norms Inventory. *Psychology of Men & Masculinity*, *4*(1), 3.
Web of Science (2006->) Missing
Google scholar (2006->): 676

Gender Role Conflict Scale
Original scale: O'Neil, J. M., Helms, B. J., Gable, R. K., David, L., & Wrightsman, L. S. (1986). Gender-Role Conflict Scale: College men's fear of femininity. *Sex roles*,*14*(5-6), 335-350.
Web of Science (2006->): 185 (full database), 20 (health research)
Google scholar (2006->): 576

Personal Attributes Questionnaire
Original scale: Spence, J. T., Helmreich, R. L., & Stapp, J. (1974). The Personal Attributes Questionnaire: A measure of sex role stereotypes and masculinity-femininity.
Web of Science (2006->): Missing
Google scholar (2006->): 428

Male Role Norms Scale
Original scale: Thompson, E. H., & Pleck, J. H. (1986). The structure of male role norms. *The American Behavioral Scientist*, *29*(5), 531.
Web of Science (2006->): 142 (full database) 15 (health)
Google scholar (2006->): 368

Masculinity Ideology
Original scale: Pleck, J. H., Sonenstein, F. L., & Ku, L. C. (1993). Masculinity ideology: Its impact on adolescent males' heterosexual relationships. *Journal of Social issues*, *49*(3), 11-29.
Web of Science (2006->): 131 (full database), 69 (health research)
Google scholar (2006->): 312

Californian Psychological Inventory
Original scale: Gough, H. G. (1975). *Manual for the California psychological inventory*. Consulting Psychologists Press.
Web of Science (2006->): Missing
Google scholar (2006->): 314

Masculine Gender Role Stress
Original scale: Eisler, R. M., & Skidmore, J. R. (1987). Masculine gender role stress scale development and component factors in the appraisal of stressful situations. *Behavior Modification*, *11*(2), 123-136.
Web of Science (2006->): 100 (full database), 19 (health)
Google scholar (2006->): 301

Traditional Machismo and Caballerismo Scale
Original scale: Arciniega, G. M., Anderson, T. C., Tovar-Blank, Z. G., & Tracey, T. J. (2008). Toward a fuller conception of Machismo: Development of a traditional Machismo and Caballerismo Scale. *Journal of Counseling Psychology*, *55*(1), 19.
Web of Science (2006->): 100 (full database), 33 (health research)
Google scholar (2006->): 276

Conformity to Feminine Norms Inventory
Original scale: Mahalik, J. R., Morray, E. B., Coonerty-Femiano, A., Ludlow, L. H., Slattery, S. M., & Smiler, A. (2005). Development of the conformity to feminine norms inventory. *Sex Roles*, *52*(7-8), 417-435.
Web of Science (2006->): 82 (full database), 17 (health research)
Google scholar (2006->): 241

Male Role Norms Inventory
Original scale: Levant, R. F., Hirsch, L. S., Celentano, E., & Cozza, T. M. (1992). The male role: An investigation of contemporary norms. *Journal of Mental Health Counseling*.
Web of Science (2006->): Missing
Google scholar (2006->): 191

Hypermasculinity Inventory
Original scale: Mosher, D. L., & Sirkin, M. (1984). Measuring a macho personality constellation. *Journal of Research in Personality*, *18*(2), 150-163.
Web of Science (2006=>): 83 (full database), 4 (health research)
Citations Google (2006=>): 251

Brannon Masculinity Scale
Original scale: Brannon, R., & Juni, S. (1984). A scale for measuring attitudes about masculinity. *Psychological Documents*, *14*.
Web of Science (2006->): 2 (full database)
Google scholar (2006->): 141

Feminine Gender Role Stress
Original scale: Gillespie, B. L., & Eisler, R. M. (1992). Development of the feminine gender role stress scale: A cognitive-behavioral measure of stress, appraisal, and coping for women. *Behavior Modification*, *16*(3), 426-438.
Web of Science (2006->): 31 (full database)
Google scholar (2006->): 78

Hyperfemininity
Original scale: Murnen, S. K., & Byrne, D. (1991). Hyperfeminity: Measurement and Initial Validation of the Construct. *The Journal of Sex Research*, 479-489.
Web of Science (2006->): 31 (full database) 4 (health)
Google scholar (2006->): 76

Adjective Check List (Masculinity and Femininity scales)
Original scale: Zuckerman, M. (1960). The development of an affect adjective check list for the measurement of anxiety. *Journal of Consulting Psychology*, *24*(5), 457.

Web of Science (2006->): 14 (full database)
Google scholar (2006->): 72

Normative Male Alexithymia Scale
Original scale: Levant, R. F., Good, G. E., Cook, S. W., O'Neil, J. M., Smalley, K. B., Owen, K., & Richmond, K. (2006). The normative Male Alexithymia Scale: Measurement of a gender-linked syndrome. *Psychology of Men & Masculinity*, *7*(4), 212.
Web of Science (2006->): Missing
Google scholar (2006->): 65

Japanese Gender Role Index
Original scale: Sugihara, Y., & Katsurada, E. (2002). Gender role development in Japanese culture: Diminishing gender role differences in a contemporary society. *Sex roles*, *47*(9-10), 443-452.
Web of Science (2006->): 13 (full database) 2 (health)
Google scholar (2006->): 44

Femininity Ideology Scale
Original scale: Levant, R., Richmond, K., Cook, S., House, A. T., & Aupont, M. (2007). The femininity ideology scale: Factor structure, reliability, convergent and discriminant validity, and social contextual variation. *Sex Roles, 57*, 373–383.
Web of Science (2006=>): 15 (full database), 3 (health research)
Google scholar (2006=>): 42

Gender Role Journey Measure
Original scale: O'Neil, J. M., Egan, J., Owen, S. V., & Murry, V. M. (1993). The gender role journey measure: Scale development and psychometric evaluation. *Sex Roles*,*28*(3-4), 167-185.
Web of science (2006=>): 10 (full database), 1 (health research)
Google scholar (2006=>): 43

Auburn Differential Masculinity Inventory
Original scale: Burk, L. R., Burkhart, B. R., & Sikorski, J. F. (2004). Construction and Preliminary Validation of the Auburn Differential Masculinity Inventory. *Psychology of Men & Masculinity*, *5*(1), 4.
Web of Science (2006->): Missing
Google scholar (2006=>): 36

PRF Andro Scale (Personality Research Form)
Original scale: Berzins, J. I., Welling, M. A., & Wetter, R. E. (1978). A new measure of psychological androgyny based on the Personality Research Form. *Journal of consulting and clinical psychology*, *46*(1), 126.
Web of Science (2006->): 8 (full database), 1 (health research)
Google scholar (2006->): 33

Sex-Role Identity Scale
Original scale: Storms, M. D. (1979). Sex role identity and its relationships to sex role attributes and sex role stereotypes. *Journal of Personality and Social Psychology*, *37*(10), 1779.
Web of science (2006=>): 15 (full database), 2 (health research)
Google scholar (2006=>): 27

New Masculine Gender Role Discrepancy
Original scale: Rummell, C. M., & Levant, R. F. (2014). Masculine gender role discrepancy strain and self-esteem. *Psychology of Men & Masculinity*, *15*(4), 419.
Too new for citations

Russian Male Norms Inventory

Original scale: Janey, B. A., Kim, T., Jampolskaja, T., Khuda, A., Larionov, A., Maksimenko, A., ... & Shipilova, A. (2013). Development of the Russian Male Norms Inventory. *Psychology of Men & Masculinity*, *14*(2), 138.
Too new for citations

Measurement of Men's Perceived Inexpressiveness
Original scale: Wong, Y. J., Horn, A. J., Gomory, A. M., & Ramos, E. (2013). Measure of Men's Perceived Inexpressiveness Norms (M2PIN): Scale development and psychometric properties. *Psychology of Men & Masculinity*, *14*(3), 288.
Too new for citations

Positive Negative Sex-role Inventory
Original scale: Berger, A., & Krahé, B. (2013). Negative attributes are gendered too: Conceptualizing and measuring positive and negative facets of sex-role identity. *European Journal of Social Psychology*, *43*(6), 516-531.
Too new for citations

Social Roles Questionnaire
Original scale: Baber, K. M., & Tucker, C. J. (2006). The social roles questionnaire: A new approach to measuring attitudes toward gender. *Sex Roles*, *54*(7-8), 459-467.
Too new for citations

Indian Gender role identity
Basu, J. (2010). Development of the Indian gender role identity scale. *Journal of the Indian Academy of Applied Psychology*, *36*(1), 25-34.
Too new for citations

Inventory of Subjective Masculinity
Wong, Y. J., Shea, M., Lafollette, J. R., Hickman, S. J., Cruz, N., & Boghokian, T. (2011). The inventory of subjective masculinity experiences: Development and psychometric properties. *The Journal of Men's Studies*,*19*(3), 236-255.
Too new for citations

Gender Relations (N=40)

The Ambivalent Sexism Inventory
Original scale: Glick, P., & Fiske, S. T. (1996). The ambivalent sexism inventory: Differentiating hostile and benevolent sexism. *Journal of personality and social psychology*, *70*(3), 491.
Web of Science (2006=>): 792 (full database), 41 (health research)
Google scholar (2006=>): 1960

Modern and Old-fashioned Sexism
Original scale: Swim, J. K., Aikin, K. J., Hall, W. S., & Hunter, B. A. (1995). Sexism and racism: Old-fashioned and modern prejudices. *Journal of Personality and Social Psychology, 68*, 199–214
Web of Science (2006=>):306 (full database), 12 (health research)
Google scholar (2006=>): 801

Dyadic Adjustment Scale across Gender
Original scale: Christensen, A., & Heavey, C. L. (1990). Gender and social structure in the demand/withdraw pattern of marital conflict. *Journal of personality and social psychology*, *59*(1), 73.
Web of science (2006=>): 193 (full database), 10 (health research)
Google scholar (2006=>): 488

Multidimensional Measure of Sexual Minority Identity

Original scale: Mohr, J., & Fassinger, R. (2000). Measuring dimensions of lesbian and gay male experience. *Measurement and Evaluation in Counseling and Development*, *33*(2), 66-66.
Web of science (2006=>): 123 (full database), 32 (health research)
Google scholar (2006=>): 346

Relationship Assessment Scale
Original scale: Hendrick, S. S., Dicke, A., & Hendrick, C. (1998). The relationship assessment scale. *Journal of Social and Personal Relationships*, *15*(1), 137-142.
Web of science (2006=>): 124 (full database), 34 (health research)
Google scholar (2006=>): 337

Neosexism Scale
Original scale: Tougas, F., Brown, R., Beaton, A. M., & Joly, S. (1995). Neosexism: Plus ça change, plus c'est pareil. *Personality and Social Psychology Bulletin*, *21*(8), 842-849.
Web of science (2006=>): 111 (full database), 1 (health research)
Google scholar (2006=>): 308

Attitudes toward Women Scale
Original scale: Spence, J. T., Helmreich, R., & Stapp, J. (1973). A short version of the Attitudes toward Women Scale (AWS). *Bulletin of the Psychonomic Society*,*2*(4), 219-220.
Web of Science (2006->): 104 (full database), 41 (health research)
Google scholar (2006->): 289

Routine and Strategic Relational Maintenance Scale
Stafford, L., Dainton, M., & Haas, S. (2000). Measuring routine and strategic relational maintenance: Scale revision, sex versus gender roles, and the prediction of relational characteristics. *Communications Monographs*, *67*(3), 306-323.
Web of Science (2006->): Missing
Google scholar (2006->): 211

Sex-Role Stereotypes
Original scale: Rosenkrantz, P., Vogel, S., Bee, H., Broverman, I., & Broverman, D. M. (1968). Sex-role stereotypes and self-concepts in college students. Journal of consulting and clinical psychology, 32(3), 287.
Web of Science (2006->): 43 (full database),
Google scholar (2006->): 210

Relationship Belief Inventory
Original scale: Eidelson, R. J., & Epstein, N. (1982). Cognition and relationship maladjustment: Development of a measure of dysfunctional relationship beliefs. *Journal of consulting and clinical psychology*, *50*(5), 715.
Web of science (2006=>): 46 (full database), 1 (health research)
Google scholar (2006=>): 196

The Romantic Beliefs Scale
Original scale: Sprecher, S., & Metts, S. (1989). Development of the Romantic Beliefs Scale and examination of the effects of gender and gender-role orientation.*Journal of Social and Personal Relationships*, *6*(4), 387-411.
Web of science (2006=>): 26 (full database), 2 (health research)
Google scholar (2006=>): 133

Relationship Authenticity
Original scale: Lopez, F. G., & Rice, K. G. (2006). Preliminary development and validation of a measure of relationship authenticity. *Journal of Counseling Psychology*, *53*(3), 362.
Web of science (2006=>): 53 (full database), 5 (health research)
Google scholar (2006=>): 123

Sex-Role Egalitarianism Scale
Original scale: Beere, C. A., King, D. W., Beere, D. B., & King, L. A. (1984). The Sex-Role Egalitarianism Scale: A measure of attitudes toward equality between the sexes. *Sex Roles*, *10*(7-8), 563-576.
Web of science (2006=>): 41 (full database), 5 (health research)
Google scholar (2006=>): 122

Quick Discrimination Index
Original scale: Ponterotto, J. G., Burkard, A., Rieger, B. P., Grieger, I., D'Onofrio, A., Dubuisson, A., Heenehan, M., Millstein, B., Parisi, M., Rath, J. F., & Sax, G. (1995). Development and initial validation of the Quick Discrimination Index (QDI). Educational and Psychological Measurement, 55, 1026-1031.
Web of science (2006=>): 31 (full database), 2 (health research)
Google scholar (2006=>): 110

Attitudes toward Traditional and Egalitarian Sex-Roles
Original scale: Larsen, K. S., & Long, E. (1988). Attitudes toward sex-roles: Traditional or egalitarian?. Sex Roles, 19(1-2), 1-12.
Web of science (2006=>): 43 (full database), 2 (health research)
Google scholar (2006=>): 110

Mutual Psychological Development Questionnaire
Original scale: Genero, N. P., Miller, J. B., Surrey, J., & Baldwin, L. M. (1992). Measuring perceived mutuality in close relationships: Validation of the Mutual Psychological Development Questionnaire. *Journal of Family Psychology*, *6*(1), 36.
Web of science (2006=>): Missing
Google scholar (2006=>): 103

Swedish Classical and Modern Sexism Scales
Original scale: Ekehammar, B., Akrami, N., & Araya, T. (2000). Development and validation of Swedish classical and modern sexism scales. *Scandinavian journal of psychology*, *41*(4), 307-314.
Web of science (2006=>): 19 (full database), 3 (health research)
Google scholar (2006=>): 65

Sex-Role Ideology
Original scale: Kalin, R., & Tilby, P. J. (1978). Development and validation of a sex-role ideology scale. *Psychological reports*, *42*(3), 731-738.
Web of Science (2006->): 19 (full database), 2 (health research)
Google scholar (2006->): 59

Quality in Relationships Inventory within Couples
Original scale: Pierce, G. R. (1994). The Quality of Relationships Inventory: Assessing the interpersonal context of social support.
Web of science (2006=>): Missing
Google scholar (2006=>): 52

Partner Behaviour Inventory
Original scale: Doss, B. D., & Christensen, A. (2006). Acceptance in romantic relationships: the frequency and acceptability of partner behavior inventory. *Psychological Assessment*, *18*(3), 289.
Web of science (2006=>): 13 (full database), 4 (health research)
Google scholar (2006=>): 45

Managing Affect and Differences Scale

Original scale: Arellano, C. M., & Markman, H. J. (1995). The Managing Affect and Differences Scale (MADS): A self-report measure assessing conflict management in couples. *Journal of Family Psychology*, *9*(3), 319
Web of science (2006=>): 8 (full database)
Google scholar (2006=>): 33

Women as Managers Scale
Original scale: Peters, L. H., Terborg, J. R., & Taynor, J. (1974). Women as Managers Scale:(WAMS): a Measure of Attitudes Toward Women in Management Positions. Journal Supplement Abstract Service of the American Psychological Association.
Web of science (2006=>): Missing
Google scholar (2006=>): 54

Gender Role Egalitarian Attitudes
Original Scale: Chang, L. (1999). Gender role egalitarian attitudes in Beijing, Hong Kong, Florida, and Michigan. *Journal of Cross-Cultural Psychology*, *30*(6), 722-741.
Web of Science (2006->): 22 (full database) 1 (health)
Google scholar (2006->): 54

Adherence to Extreme Gender Role Beliefs
Original scale: Hamburger, M. E., Hogben, M., McGowan, S., & Dawson, L. J. (1996). Assessing Hypergender Ideologies: Development and Initial Validation of a Gender-Neutral Measure of Adherence to Extreme Gender-Role Beliefs. Journal of Research in Personality, 30(2), 157-178.
Web of Science (2006->): 23 (full database) 3 (health)
Google scholar (2006->): 45

Gender Attitude Inventory
Original scale: Ashmore, R. D., Del Boca, F. K., & Bilder, S. M. (1995). Construction and validation of the Gender Attitude Inventory, a structured inventory to assess multiple dimensions of gender attitudes. *Sex roles*, *32*(11-12), 753-785.
Web of Science (2006->): 15 (full database)
Google scholar (2006->): 45

Sex-Role Orientation
Original scale: Brogan, D., & Kutner, N. G. (1976). Measuring sex-role orientation: A normative approach. *Journal of Marriage and the Family*, 31-40.
Web of science (2006=>): 12 (full database) 4 (health)
Google scholar (2006=>): 44

Gender ideology as an identity
Original scale: Kroska, A. (2000). Conceptualizing and measuring gender ideology as an identity. *Gender & Society*, *14*(3), 368-394.
Web of Science (2006->): 12 (full database) 2 (health)
Google scholar (2006->): 36

Gender Role Beliefs Scale
Original scale: Kerr, P. S., & Holden, R. R. (1996). Development of the gender role beliefs scale (GRBS). Journal of Social Behavior and Personality, 11(5), 3.
Web of Science (2006->): 9 (full database)
Google scholar (2006->): 34

Male Attitude Norms Inventory II
Original scale: Luyt, R. (2005). The Male Attitude Norms Inventory-II A Measure of Masculinity Ideology in South Africa. *Men and Masculinities*, *8*(2), 208-229.
Web of science (2006=>): Missing
Google scholar (2006=>): 36

Sexist Attitudes toward Women
Original scale: Benson, P. L., Institute, S., & Vincent, S. (1980). Development and validation of the sexist attitudes toward women stale (SATWS). Psychology of Women Quarterly, 5(2), 276-291.
Web of science (2006=>): 8 (full database), 4 (health)
Google scholar (2006=>): 31

Race and Gender-Specific Stress Measure for African-American Women
Original scale: Jackson, F. M. (2005). The Development of a Race and Gender-Specific Stress Measure for African- American Women: Jackson, Hogue, Phillips Contextualized Stress Measure. *Ethnicity & disease*, *15*(4), 594-600.
Web of science (2006=>): 16 (full database), 10 (health research)
Google scholar (2006=>): 26

Egalitarian Sex Role Attitudes
Suzuki, A. (1991). Egalitarian sex role attitudes: Scale development and comparison of American and Japanese women. Sex Roles, 24(5-6), 245-259.
Web of science (2006=>): 15 (full database), 4 (health research)
Google scholar (2006=>): 26

Schedule of Sexist Events
Original scale: Yoder, J. D., & McDonald, T. W. (1998). Measuring sexist discrimination in the workplace: Support for the validity of the schedule of sexist events. *Psychology of Women Quarterly, 22*, 487–491.
Google scholar (2006=>): 22
Web of Science (2006=>): 8 (full database) 1 (health research)

German Relationship Assessment Scale
Original scale: Dinkel, A., & Balck, F. (2005). An evaluation of the German relationship assessment scale. *Swiss Journal of Psychology*, *64*(4), 259-263.
Web of science (2006=>): 6 (full database), 1 (health research)
Google scholar (2006=>): 22

Concord-Index (measure of family relationships)
Original scale: Lee, P. H., Stewart, S. M., Lun, V., Bond, M. H., Yu, X., & Lam, T. H. (2012). Validating the concord index as a measure of family relationships in China.*Journal of Family Psychology*, *26*(6), 906.
Too new for citations

Conflict Disengagement Inventory (CDI)
Original scale: Sanford, K. (2014). A latent change score model of conflict resolution in couples Are negative behaviors bad, benign, or beneficial?. *Journal of Social and Personal Relationships*, 0265407513518156.
Too new for citations

Gender Minority Stress and Resilience
Original scale: Testa, R. J., Habarth, J., Peta, J., Balsam, K., & Bockting, W. (2015). Development of the Gender Minority Stress and Resilience Measure .*Psychology of Sexual Orientation and Gender Diversity*, *2*(1), 65.
Too new for citations

Gender-Based Attitudes Toward Marriage and Child Rearing
Original scale: Adams, M., Coltrane, S., & Parke, R. D. (2007). Cross-ethnic applicability of the gender-based attitudes toward marriage and child rearing scales. *Sex roles*,*56*(5-6), 325-339.
Too new for citations

Patriarchal Beliefs Scale
Original scale: Yoon, E., Adams, K., Hogge, I., Bruner, J. P., Surya, S., & Bryant, F. B. (2015). Development and validation of the Patriarchal Beliefs Scale. *Journal of counseling psychology*, *62*(2), 264.
Too new for citations

African American Men's Gendered Racism Stress Inventory
Original scale: Schwing, A. E., Wong, Y. J., & Fann, M. D. (2013). Development and validation of the African American Men's Gendered Racism Stress Inventory. *Psychology of Men & Masculinity*, *14*(1), 16.
Too new for citations

Ineligible Scales (N=57)

NEO Personality Inventory (broader scale)
Original scale: Costa, P. T., & McCrae, R. R. (1985). *The NEO personality inventory: Manual, form S and form R*. Psychological Assessment Resources.
Web of Science (2006->): Missing
Google scholar (2006->): 2200
Justification: Doesn't measure gender.

Conformity to Traditional Masculinity and Relationship Satisfaction
Original scale: Burn, S. M., & Ward, A. Z. (2005). Men's Conformity to Traditional Masculinity and Relationship Satisfaction. *Psychology of Men & Masculinity*, *6*(4), 254.
Web of Science (2006->): Missing
Google scholar (2006->): 99
Justification: Its traits and dimensions are based on the Conformity to Masculine Norms scale. This scale is already included.

Gender Diagnosticity
Original scale: Lippa, R., & Connelly, S. (1990). Gender diagnosticity: A new Bayesian approach to gender-related individual differences. *Journal of Personality and Social Psychology*, *59*(5), 1051.
Web of Science (2006->): 44 (full database), 5 (health research)
Google scholar (2006->): 61
Justification: Measure used to predict not measure gender

Gender Differences in Sexual Attitudes
Original scale: Hendrick, S., Hendrick, C., Slapion-Foote, M. J., & Foote, F. H. (1985). Gender differences in sexual attitudes. *Journal of Personality and Social Psychology*,*48*(6), 1630.
Web of Science (2006->): 21 (full database), 9 (health research)
Google scholar (2006->):61
Justification: Focuses on sexuality, not gender.

Masculine Gender Identity In females
Original scale: Blanchard, R., & Freund, K. (1983). Measuring masculine gender identity in females. *Journal of Consulting and Clinical Psychology*, *51*(2), 205.
Web of Science (2006->): 9 (full database) 5 (health)
Google scholar (2006->): 21
Justification: Specifically designed to measure transgender behavior. Items (behaviors and sentiments) do not capture gender related traits and characteristics.

Male Assessment of Self-Objectification
Original scale: Daniel, S., Bridges, S. K., & Martens, M. P. (2014). The development and validation of the Male Assessment of Self-Objectification (MASO). *Psychology of Men & Masculinity*, *15*(1), 78.
Too new for citations

Justification: Measures Body-related aspects of gender (not deemed relevant for our measure)

Masculine Body Ideal Distress Scale
Original scale: Kimmel, S. B., & Mahalik, J. R. (2004). Measuring masculine body ideal distress: Development of a measure. *International Journal of Men's Health*,*3*(1), 1.
Too new for citations
Justification: Measures Body-related aspects of gender (not deemed relevant for our measure)

Subjective Masculinity stress
Original scale: Wong, Y. J., Shea, M., Hickman, S. J., LaFollette, J. R., Cruz, N., & Boghokian, T. (2013). The Subjective Masculinity Stress Scale: Scale development and psychometric properties. *Psychology of Men & Masculinity*,*14*(2), 148.
Too new for citations
Justification: Does not rely on predefined gender characteristics and traits. Respondents define their own "gender stressors" and hereafter rate them.

Men's Perceived Inexpressiveness Norms
Original scale: Wong, Y. J., Horn, A. J., Gomory, A. M., & Ramos, E. (2013). Measure of Men's Perceived Inexpressiveness Norms (M2PIN): Scale development and psychometric properties. *Psychology of Men & Masculinity*, *14*(3), 288.
Too new for citations
Justification: Measures expressivity in men, not gender.

Reference Group Identity Dependence Scale
Original scale: Wade, J. C., & Gelso, C. J. (1998). Reference Group Identity Dependence Scale A Measure of Male Identity. *The Counseling Psychologist*, *26*(3), 384-412.
Web of Science (2006->): 11 (full database), 1 (health research)
Google scholar (2006->): 28
Justification: Does not address gender traits and characteristics

A Gender-Based Measurement Invariance Study of the Sociocultural Attitudes toward Appearance Questionnaire
Original scale: Wheeler, D. L., Vassar, M., & Hale, W. D. (2011). A gender-based measurement invariance study of the Sociocultural Attitudes Toward Appearance Questionnaire-3. *Body image*, *8*(2), 168-172.
Too new for citations
Justification: Measures Body-related aspects of gender (not deemed relevant for our measure)

LGBT Ally Identity Measure
Original scale: Jones, K. N., Brewster, M. E., & Jones, J. A. (2014). The creation and validation of the LGBT Ally Identity Measure. *Psychology of Sexual Orientation and Gender Diversity*, *1*(2), 181.
Too new for citations
Justification: Does not measure gender

O' Kelly Women's Belief Scale
Original scale: O'Kelly, M. (2011). Psychometric properties of the O'Kelly women's belief scales. *Journal of Rational-Emotive & Cognitive-Behavior Therapy*, *29*(3), 145-157.
Too new for citations
Justification: Does not address gender traits and characteristics

Lesbian and Gay Identity Scale
Original scale: Mohr, J., & Fassinger, R. (2000). Measuring dimensions of lesbian and gay male experience. *Measurement and Evaluation in Counseling and Development*, *33*(2), 66-66.
Too new for citations
Justification: Doesn't measure gender

Wiggins Interpersonal Behavior Circle
Original scale: Wiggins, J. S. (1979). A psychological taxonomy of trait-descriptive terms: The interpersonal domain. *Journal of personality and social psychology*, *37*(3), 395.
Too new for citations
Justification: Doesn't measure gender

Social Sex-Role Inventory (DSI)
Original scale: Shively, M. G., & De Cecco, J. P. (1977). Components of sexual identity. *Journal of homosexuality*, *3*(1), 41-48.
Web of Science (2006->): 23 (full database), 7 (health research)
Google scholar (2006->): 142
Justification: Doesn't measure gender

Masculine Gender Role Discrepancy
Original scale: Pleck, J. H. (1995). The gender role strain paradigm: An update.
Web of Science (2006->): Missing
Google scholar (2006->): 590
Justification: This is a theory, not a scale

Omnibus Personality Inventory
Original scale: Heist, P., & Yonge, G. D. (1968). *Omnibus personality inventory*. Psychological Corporation.
Justification: Fewer than 20 citations since 2006

Women's Role Strain Inventory
Original scale: Lengacher, C. A. (1993). Development of a predictive model for role strain in registered nurses returning to school. *The Journal of nursing education*, *32*(7), 301-308.
Justification: Fewer than 20 citations since 2006

Australian Sex-Role Scale
Original scale: Antill, J. K., Cunningham, J. D., Russell, G., & Thompson, N. L. (1981). An Australian sex-role scale. *Australian Journal of Psychology*, *33*(2), 169-183.
Justification: Fewer than 20 citations since 2006

Home Career Conflict Measure
Original scale: Farmer, H. S. (1984). Development of a measure of home-career conflict related to career motivation in college women. *Sex Roles*, *10*(9-10), 663-675.
Justification: Fewer than 20 citations since 2006

Sex-Role Blending
Original scale: Heilbrun, A. B. (1981). Gender differences in the functional linkage between androgyny, social cognition, and competence. *Journal of Personality and Social psychology*, *41*(6), 1106.
Justification: Fewer than 20 citations since 2006

Chinese Gender Role Stress
Original scale: Tang, C. S. K., & Lau, B. H. B. (1995). The assessment of gender role stress for Chinese. *Sex Roles*, *33*(7-8), 587-595.
Justification: Fewer than 20 citations since 2006

Feminine Gender Identity Scale
Original scale: Freund, K., Nagler, E., Langevin, R., Zajac, A., & Steiner, B. (1974). Measuring feminine gender identity in homosexual males. *Archives of Sexual Behavior*,*3*(3), 249-260.
Justification: Fewer than 20 citations since 2006

Self and Peer-Related Scale of Female and Male Roles and Attributes
Original scale: Chang, L., & McBride-Chang, C. (1997). Self-and peer-ratings of female and male roles and attributes. *The Journal of social psychology*, *137*(4), 527-529.
Justification: Fewer than 20 citations since 2006

Behavioral Self-Report of Femininity
Original scale: Greene, K. S., & Gynther, M. D. (1994). Another femininity scale? *Psychological reports*, *75*(1), 163-170.
Justification: Fewer than 20 citations since 2006

The Femininity Study
Original scale: Thorne, F. C. (1977). The measurement of femininity. *Journal of clinical psychology*, *33*(S1), 5-10.
Justification: Fewer than 20 citations since 2006

Undesirable Characteristics Scale
Original scale: Socially-undesirable sex-correlated characteristics: implications for androgyny and adjustment. *Journal of Consulting and Clinical Psychology,* 45, 1185-1186.
Justification: Fewer than 20 citations since 2006

Measure of Adult Stereotypic Sex-Role Concepts of Masculinity
Original scale: Newman, R. C. (1976). Development and Standardization of Measures of Stereotypic Sex-Role Concepts and of Sex-Role Adoption in Adults. *Psychological reports*, *39*(2), 623-630.
Justification: Fewer than 20 citations since 2006

Michigan Gender Identity Test (MIGIT)
Original scale: Dull, C. Y., Catford, J. C., Guiora, A. Z., Beit-Hallahmi, B., Paluszny, M., & Cooley, R. E. (1975). The Michigan gender identity test (MIGIT). *Comprehensive psychiatry*, *16*(6), 581-592.
Justification: Fewer than 20 citations since 2006

Sex Rep
Original scale: Baldwin, A. C., Critelli, J. W., Stevens, L. C., & Russell, S. (1986). Androgyny and sex role measurement: A personal construct approach. *Journal of Personality and Social Psychology*, *51*(5), 1081.
Justification: Fewer than 20 citations since 2006

Attitudes toward Masculinity Transcendence Scale
Original scale: Moreland, J., & Van Tuinen, M. (1978). The attitude toward masculinity transcendence scale. *Unpublished manuscript, Southern Illinois University at Carbondale*.
Justification: Fewer than 20 citations since 2006

Scales for Investigation of the Dual-Career Family
Original scale: Pendleton, B. F., Poloma, M. M., & Garland, T. N. (1980). Scales for investigation of the dual-career family. *Journal of Marriage and the Family*, 269-276.
Justification: Fewer than 20 citations since 2006

Sex-Role Antecedents Scale
Original scale: Mast, D. L., & Herron, W. G. (1986). The sex-role antecendents scales. *Perceptual and Motor Skills*, *63*(1), 27-56.
Justification: Fewer than 20 citations since 2006

Multi-dimensional Sex-role Inventory

Original scale: Bernard, L. C., & Wood, J. (1990). Further observations on the multidimensional aspects of masculinity-femininity: The multidimensional sex role inventory-revised. *Journal of Social Behavior and Personality*, *5*(4), 205.
Justification: Fewer than 20 citations since 2006

Masculine role inventory
Original scale: Snell Jr, W. E. (1986). The masculine role inventory: Components and correlates. *Sex Roles*, *15*(7-8), 443-455.
Justification: Fewer than 20 citations since 2006

Multifaceted Gender identity questionnaire
Original scale: Willemsen, T. M., & Fischer, A. H. (1999). Assessing multiple facets of gender identity: The Gender Identity Questionnaire. *Psychological Reports*, *84*(2), 561-562.
Justification: Fewer than 20 citations since 2006

Masculine Behavior
Original scale: Snell Jr, W. E. (1989). Development and validation of the Masculine Behavior Scale: A measure of behaviors stereotypically attributed to males vs. females.*Sex Roles*, *21*(11-12), 749-767.
Justification: Fewer than 20 citations since 2006

Masculine and Feminine Self-Disclosure Scale
Original scale: Snell Jr, W. E., Belk, S. S., & Hawkins II, R. C. (1986). The Masculine and Feminine Self-Disclosure Scale: The politics of masculine and feminine self-presentation. *Sex Roles*, *15*(5-6), 249-267.
Justification: Fewer than 20 citations since 2006

Male female relations questionnaire
Original scale: Sherman, P. J., & Spence, J. T. (1997). A COMPARISON OF TWO COHORTS OF COLLEGE STUDENTS IN RESPONSES TO THE MALE-FEMALE RELATIONS QUESTIONNAIRE. *Psychology of Women Quarterly*, *21*(2), 265-278.
Justification: Fewer than 20 citations since 2006

Machismo
Original scale: Villemez, W. J., & Touhey, J. C. (1977). A measure of individual differences in sex stereotyping and sex discrimination: The “Macho” scale. *Psychological Reports*, *41*(2), 411-415.
Justification: Fewer than 20 citations since 2006

Feminine Gender Identity Scale
Original scale: Willemsen, T. M., & Fischer, A. H. (1999). Assessing multiple facets of gender identity: The Gender Identity Questionnaire. *Psychological Reports*, *84*(2), 561-562.
Justification: Fewer than 20 citations since 2006

Womanist identity attitude scale
Original scale: Moradi, B., Yoder, J. D., & Berendsen, L. L. (2004). An evaluation of the psychometric properties of the womanist identity attitudes scale. *Sex Roles*,*50*(3-4), 253-266.
Justification: Fewer than 20 citations since 2006

Assessing the Dynamics of Gender in Couples and Families (Gendergram)
Original scale: White, M. B., & Tyson-Rawson, K. J. (1995). Assessing the dynamics of gender in couples and families: The gendergram. *Family Relations*, 253-260.
Justification: Fewer than 20 citations since 2006

Attitudes toward Multiple Role Planning

Original scale: Weitzman, L. M., & Fitzgerald, L. F. (1996). The development and initial validation of scales to assess attitudes toward multiple role planning. *Journal of Career Assessment*, *4*(3), 269-284.
Justification: Fewer than 20 citations since 2006

Traditional vs. Egalitarian Roles in Marriages
Original scale: Altrocchi, J., & Crosby, R. D. (1989). Clarifying and measuring the concept of traditional vs. egalitarian roles in marriages. *Sex roles*, *20*(11-12), 639-648.
Justification: Fewer than 20 citations since 2006

Women in Society Questionnaire
Original scale: Walker, L. (1994). Attitudes to minorities: Survey evidence of Western Australians' attitudes to Aborigines, Asians, and women. *Australian Journal of Psychology*, *46*(3), 137-143
Justification: Fewer than 20 citations since 2006

Attitudes to females' social roles
Original scale: Slade, P., & Jenner, F. A. (1978). Questionnaire measuring attitudes to females' social roles. Psychological Reports, 43(2), 351-354.
Justification: Fewer than 20 citations since 2006

Maferr Inventory of Feminine Values
Original scale: Steinmann, A. G. (1979). Maferr Inventory of Feminine Values: Specimen Set (and) Manual Series for the Interpretation of the Maferr Inventory of Feminine Values (MIFV).
Justification: Fewer than 20 citations since 2006

Tridimensional Sexism Scale
Original scale: Rombough, S., & Ventimiglia, J. C. (1981). Sexism: A tri-dimensional phenomenon. *Sex Roles*, *7*(7), 747-755.
Justification: Fewer than 20 citations since 2006

Sex Role Questionnaire
Original scale: Jean, P. J., & Reynolds, C. R. (1980). Development of the Bias in Attitudes Survey: A sex-role questionnaire. *Journal of Psychology*, *104*(2), 269.
Justification: Fewer than 20 citations since 2006

Traditional and Liberated Males' Attitudes
Original scale: Fiebert, M. S. (1983). Measuring traditional and liberated males' attitudes. Perceptual and motor skills.
Justification: Fewer than 20 citations since 2006

Superficiality and the Dimensionality of Sexism
Original scale: Korth, B. (1978). Superficiality and the Dimensionality of Sexism. Applied Psychological Measurement, 2(1), 51-61.
Justification: Fewer than 20 citations since 2006

The Traditional-Liberated Content Scale
Original scale: Fiebert, M. S., & Vera, W. (1985). Test-retest reliability of a male sex-role attitude survey: The traditional-liberated content scale. *Perceptual and motor skills*, *60*(1).
Justification: Fewer than 20 citations since 2006

Multidimensional Aversion to Women who Work Scale
Original scale: Valentine, S. (2001). Development of a brief multidimensional aversion to women who work scale. *Sex Roles*, *44*(11-12), 773-787.
Justification: Fewer than 20 citations since 2006

Wellesley Role Orientation Scale

Original scal: Alper, T. G. (1973). The relationship between role orientation and achievement motivation in college women. *Journal of Personality*.
Justification: Fewer than 20 citations since 2006

Sex Role Behavior Scale
Original scale: Orlofsky, J. L., Ramsden, M. W., & Cohen, R. S. (1982). Development of the revised sex-role behavior scale. *Journal of Personality Assessment, 46*, 632–638.
Justification: Fewer than 20 citations since 2006

Step 4: Developing a core list of gender variables

This section describes how we identified the core gender variables for each of the three categories gender-related traits, gender norms and gender relations.

Identifying core variables for gender norms

Through the literature search, we identified three eligible measures pertaining to the gender norms category. The first two measures (see below) concern child-rearing and co-parenting behavior. These aspects will be captured with a slightly broader variable focusing on various forms of caregiving. We hereby extend the restricted focus on "nuclear" family formations proposed by existing measures. The third measure focuses on dual-career wives' orientation towards domestic labor. We also intend to capture this aspect with a broader variable focusing on time-use for both women and men. An additional variable focusing work-related physical and psychological strain will also be included under this category.

Caregiving
- Parental Child-rearing Behavior Scale
- Co-parenting and family rating system

Time-use
- The orientation towards domestic labor

Identifying core variables for gender-related traits

In developing our final list of gender-related traits, we began by harvesting all relevant characteristics and traits covered by the 31 eligible gender-related traits scales identified in Step 3. Next, we aggregated similar and closely related gender traits and characteristics into 17 clusters, each capturing distinct aspects of an individual's gender identity, using inductive coding categories. Numerous cluster-solutions (i.e. solutions varying on the total number of clusters since characteristics and traits can be merged or split into smaller or larger clusters) were scrutinized as part of this step. Consensus was ultimately established on the 17-cluster solution as the most intuitively meaningful and feasible way of structuring the many traits and characteristics into key gender-related traits variables for health research (Table S3). We then ranked the clusters based on occurrences (i.e. number of characteristics and traits pertaining to each cluster) to obtain a closer understanding of their prevalence across existing scales. All in all, this strategy helped us make sense of the literature and carve out a condensed list of core gender-related traits variables. Specifically, we selected the four most prevalent clusters emerging from our coding. Risk-taking is not

among the "top-scorers," but has been included as a fifth variable given its well-documented links to health outcomes.

1. Emotionality and expressiveness (37 occurrences) (Captured by *Expressive*)
2. Empathy, caring and nurturing (37 occurrences) (Captured by *Empathetic/caring/nurturing*)
3. Self-reliance and independence (18 occurrences) (Captured by *Self-reliant/independent*)
4. Striving for status, competitiveness (17 occurrences) (Captured by *Competitive/striving*)
5. Risk-taking (5 occurrences) (Captured by *Sensation-seeking/risk-taking*)

Identifying core variables for gender relations

The literature search resulted in 40 scales pertaining to the gender relations category. Specifically, these scales capture two core variables for gender relations: equality in family relationships and discrimination. An additional variable focusing more broadly on social support will also be included under this category.

Gender discrimination

- Gender Minority Stress and Resilience
- Multidimensional Measure of Sexual Minority Identity
- The Ambivalent Sexism Inventory
- Race and Gender-Specific Stress Measure for African-American Women
- Schedule of Sexist Events
- African American Men's Gendered Racism Stress Inventory

Sexism and attitudes

- Patriarchal Beliefs Scale
- Modern and Old-Fashioned Sexism
- Sex-Role Ideology
- Gender Ideology as an Identity
- Gender Attitude Inventory
- Quick Discrimination Index
- Sex-Role Stereotypes
- Attitudes toward Traditional and Egalitarian Sex-Roles
- Women as Managers Scale
- Neosexism Scale
- Swedish Classical and Modern Sexism Scale
- Sexist Attitudes toward Women
- Sex-Role Egalitarianism Scale
- Attitudes toward Women Scale
- Gender Role Egalitarian Attitudes Scale
- Adherence to Extreme Gender Role Beliefs
- Sex-Role Orientation
- Gender Role Beliefs Scale
- Male Attitude Norms Inventory II
- Egalitarian Sex Role Attitudes

Equality in family relationships

- Dyadic Adjustment Scale across Gender
- Quality in Relationships Inventory within Couples
- Conflict Disengagement Inventory (CDI)
- German Relationship Assessment Scale (*Same as Relationship Assessment Scale)
- Partner Behavior Inventory
- Relationship Assessment Scale
- Relationship Belief Inventory
- Managing Affect and Differences Scale
- Concord-Index (measure of family relationships)
- Mutual Psychological Development Questionnaire (measuring perceived mutuality in close relationships)
- Relationship Authenticity (Authenticity in Relationships Scale)
- The Romantic Beliefs Scale
- Routine and Strategic Relational Maintenance Scale
- Gender-Based Attitudes Toward Marriage and Child Rearing

Step 5: Identifying measures for each gender variable

This section documents the search strategy used to identify additional construct specific measures of relevance to our gender variables. We for searched for meta-analyses in PsycINFO and PubMed and restricted the time-span to the period 2006 through 2015.

Search limitations. We used the following database-specific search limitations:

PsycINFO: Age-group: Adulthood (18 years or older); Record type: Peer-reviewed journal, Abstract collection, Peer-reviewed articles; Methodology: Meta-Analysis, Metasynthesis, systematic review; Language: English; Population: Human; PsycINFO Classifiers: “Tests and testing”; “Personality Scales &Inventories”; “Clinical Psychological Testing”; “Health Psychology Testing”; “Educational measurement”; “Occupational and employment testing”; “Consumer opinion and attitudes”.

PUBMED: Ages: all ages; Species: Humans; Language: English; Article types: all.

Search strings. The search terms used in this part of the literature search are listed in Table S4.

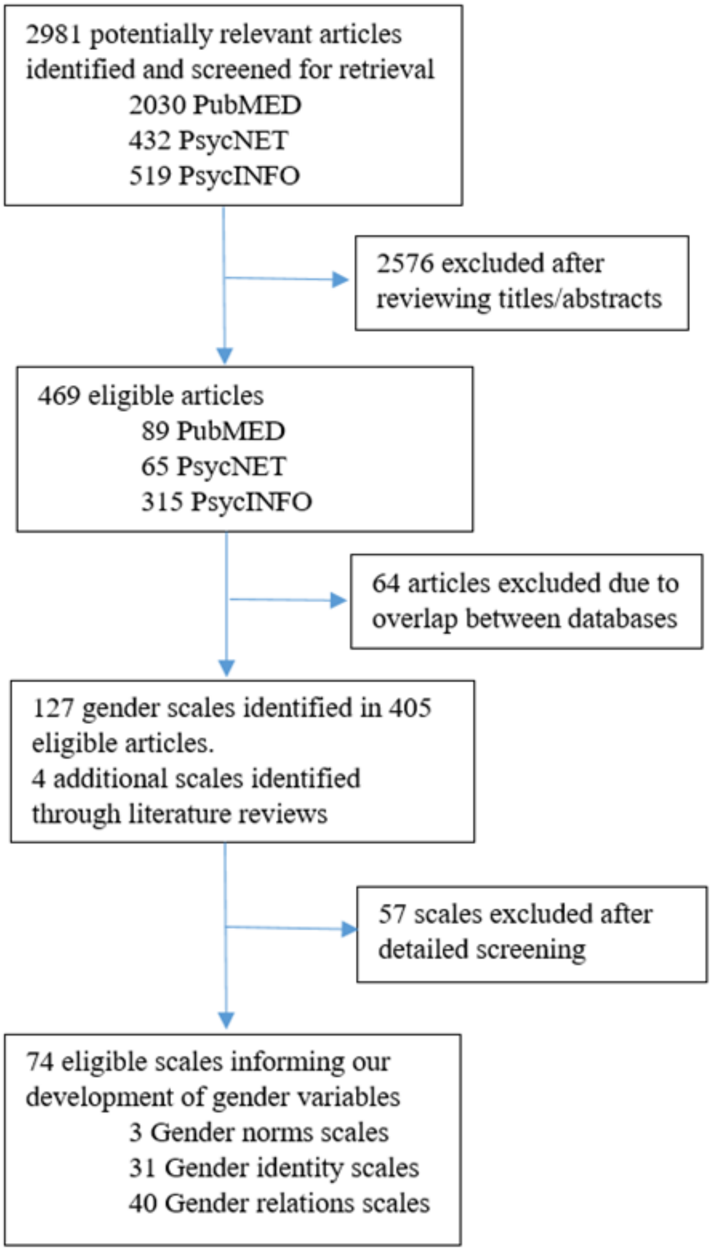

Fig. S1. Flowchart of article inclusion and exclusion in the literature search.

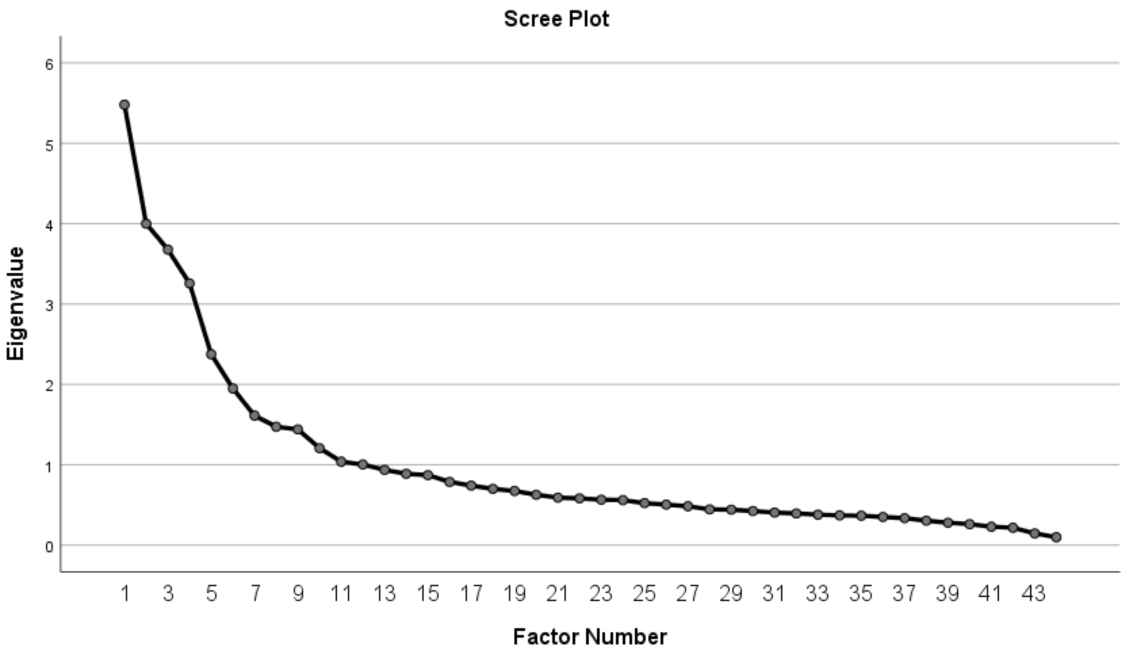

Fig. S2. Screeplot of the factor analysis reported in Table S8.

Table S1. Item phrasing and descriptive statistics for the 44 potentially relevant gender-related items.

				SAMPLE 1					SAMPLE 2						SAMPLE 3			
**Items**	Item name	Min	Max	N	Mean	S.D.	Median	IQR	N	Mean	S.D.	Median	IQR	N	Mean	S.D.	Median	IQR
Gender Norms																		
In the past year, how often did you feel emotionally exhausted because of your caretaking responsibilities?	careemot*	1	5	2032	1.59	1.07	1	1	2062	1.60	1.04	1	1	462	1.57	1.02	1	1
In the past year, how often did you feel physically exhausted because of your caretaking responsibilities?	carephys*	1	5	2032	1.58	1.05	1	1	2062	1.58	1.03	1	1	462	1.53	.97	1	1
In the past year, how often have your caretaking responsibilities caused you to worry about the future?	carefut*	1	5	2032	1.67	1.21	1	1	2062	1.67	1.17	1	1	462	1.64	1.16	1	1
On average, how many hours per weekday do you spend on the following: Sleep	timesleep	1	24	2017	6.88	2.26	7	2	2068	6.86	2.17	7	2	457	6.80	3.06	7	2
On average, how many hours per weekday do you spend on the following: Work (paid work, studying, internships, etc.)	timework	1	24	2017	6.74	3.35	8	4	2069	7.13	3.35	8	4	457	5.96	4.58	8	9
On average, how many hours per weekday do you spend on the following: Household Chores (yard work, food shopping, cooking, cleaning up, repairs, etc.)	timehouse	1	24	2019	2.07	1.66	2	1.5	2070	2.22	1.62	2	2	457	2.6	2.5	2	2
On average, how many hours per weekday do you spend on the following: Leisure (free time activities, relaxing, chatting, entertainment, etc.)	timeleisure	1	4	2018	4.31	3.23	4	4	2069	3.85	2.99	3	3	456	3.75	2.99	3	3
On average, how many hours per weekday do you spend on the following: Exercise (walking or biking to and from work, going to the gym, playing sports, etc.)	timeexc	1	24	2017	0.97	1.26	1	1	2066	0.925	1.26	1	1	456	1.23	1.18	1	1.5
On average, how many hours per weekday do you spend on the following: Taking care of	timecare	1	24	2014	1.68	2.80	0	2	2068	1.813	2.64	0.25	3	457	1.86	3.46	0	2.5

someone in need (caring for children, elders, partners in need, etc.)																		
On average, how many hours per weekday do you spend on the following: Commuting	timecom	1	24	2016	0.87	1.06	1	1	2065	0.86	1.01	1	1	457	1.05	1.55	1	2
How often does your job require working fast?	workspeed*	1	5	2027	2.79	1.36	3	3	2062	2.81	1.34	3	3	455	2.42	1.42	2	3
How often does your job involve repetitive tasks?	workrep*	1	5	2027	2.83	1.39	3	3	2062	2.83	1.34	3	3	455	2.24	1.3	2	2
How often do you feel emotionally exhausted from your work activities?	workemot*	1	5	2027	2.29	1.20	2	2	2062	2.26	1.14	2	2	455	2.05	1.19	2	2
How often do you feel physically exhausted from your work activities?	workphys*	1	5	2027	2.18	1.18	2	2	2062	2.13	1.13	2	2	455	1.89	1.14	1	2
How often does your job involve risk of harm or injury?	workrisk*	1	5	2027	1.47	0.85	1	1	2062	1.52	0.93	1	1	455	1.4	.82	1	1
How often does your job involve hazards, such as smoke, heat, noise, or chemicals?	workhaz*	1	5	2027	1.44	0.91	1	0	2062	1.48	0.96	1	1	455	1.37	.88	1	0
Gender-related traits																		
How often do you find yourself competing with others in situations that do not call for competition?	compdom	1	5	2043	2.47	0.91	2	1	2101	2.42	0.88	2	1	463	2.37	.89	2	1
How competitive are you compared to others?	compgen	1	5	2042	2.95	1.13	3	2	2100	2.96	1.13	3	2	471	3.10	1.18	3	2
In general, how prepared are you to take risks?	riskgen	1	5	2039	2.75	0.94	3	1	2095	2.75	0.91	3	1	467	2.99	.930	3	2
How prepared are you to take risks when making financial decisions?	riskfinan	1	5	2039	2.65	1.12	3	1	2095	2.70	1.10	3	1	467	2.76	1.09	3	2
How prepared are you to take risks when it comes to recreational activities?	riskrecrea	1	5	2039	3.05	1.11	3	2	2095	2.98	1.11	3	2	467	3.11	1.09	3	2
How important is it for you to be independent?	indepgen	1	5	2037	4.05	0.92	4	1	2094	4.16	0.86	4	1	467	4.18	.824	4	1
When you are in need, how often do you turn to others for help?	Indephelp	1	5	2037	3.29	0.83	3	1	2094	3.42	0.78	3	1	467	3.09	0.92	3	2
How important is it for you to solve your problems on your own?	indepprob	1	5	2036	3.81	0.94	4	2	2094	3.93	0.885	4	2	467	3.71	.973	4	1

How often do you worry about what other people think about you?	commother	1	5	2036	2.95	1.05	3	2	2091	2.72	1.04	3	1	466	2.90	0.99	3	2
When making an important decision in your personal life, how often do you take other people's needs into account?	commdecis	1	5	2035	3.54	0.92	4	1	2091	3.54	0.91	4	1	466	3.83	0.87	4	1
How often do friends talk to you about their problems?	commfriend	1	5	2036	3.23	0.95	3	1	2091	3.23	0.93	3	1	466	3.49	.95	4	1
How easy is it for you to spot when someone in a group is feeling uncomfortable?	commemp	1	5	2036	3.49	1.02	4	1	2091	3.52	1.00	4	1	466	3.77	0.93	4	1
How often do you talk to your friends about your problems?	exprfriend	1	5	2032	2.60	0.91	3	1	2088	2.55	0.88	2	1	463	2.81	.88	3	1
How easy is it for you to express what you are feeling to others?	exprother	1	5	2032	2.64	1.15	3	1	2089	2.75	1.17	3	2	463	2.89	1.11	3	2
How easy is it for you to understand your own feelings?	exprown	1	5	2032	3.36	1.09	3	1	2089	3.49	1.01	4	1	463	3.36	1.05	3	1
When you are in need, how easy is it for you to ask other people for help?	Exprhelp[X]	1	5	2032	2.31	1.07	2	2	2089	2.20	1.08	2	2	463	2.48	1.10	2	1
Gender relations																		
In the past year, how often did you have someone to give you advice?	socsupadv	1	5	2018	3.12	1.04	3	2	2061	3.00	1.02	3	2	455	3.24	1.10	3	2
In the past year, how often did you have someone to show you love and affection?	socsuplove	1	5	2018	3.56	1.18	4	2	2061	3.61	1.16	4	2	455	3.75	1.16	4	2
In the past year, how often did you have someone to help you with daily chores?	socsupchores	1	5	2018	2.96	1.21	3	2	2061	2.89	1.24	3	2	455	3.02	1.28	3	2
In the past year, how often did you feel lonely?	socsuplone	1	5	2018	3.36	1.06	3	1	2061	3.58	1.06	4	1	455	2.47	1.01	2	1
Because of your gender, how often have you felt discriminated against?	discrgen	1	5	2018	1.96	0.95	2	2	2061	1.81	0.89	2	1	455	2.05	0.92	2	1
Because of your gender, how often have you felt discriminated against when getting hired?	discrhire*	1	5	2051	1.59	0.93	1	1	2061	1.60	0.89	1	1	455	1.47	0.82	1	1
Because of your gender, how often have you felt discriminated against when at school?	discred	1	5	2018	1.63	0.91	1	1	2060	1.49	0.81	1	1	351	1.30	0.69	1	0
Because of your gender, how often have you felt	discrmed	1	5	2018	1.53	0.93	1	1	2061	1.37	0.78	1	0	455	1.47	0.84	1	1

discriminated against when receiving medical care?																	
Because of your gender, how often have you felt discriminated against in public settings?	discrpub	1	5	2018	1.89	0.96	1	2	2061	1.71	0.89	1	1	455	1.89	0.95	2	1
Because of your gender, how often have you felt discriminated against in your family?	discrfam	1	5	2018	1.58	0.94	1	1	2061	1.47	0.849	1	1	455	1.48	0.85	1	1
In the past year, how would you describe the quality of your relationship with your close relatives?	qualfam	1	5	2017	3.59	1.02	4	1	2059	3.53	1.02	2	1	452	3.81	0.95	4	1
In the past month, how often have you argued with close relatives?	famarg	1	5	2017	4.15	0.91	4	1	2059	4.31	0.85	1	1	452	4.34	0.80	2	1

Note: Items marked with * are recoded so that people not currently taking care of someone or not currently employed (or employed in the past) have been ascribed the value one. The recoding varies depending on the item. Items marked with ^{x} are reverse-scored (see specifications in Table S2). Item with ^{x} has been reverse coded for the EFA and CFA.

Table S2. Response options for all 44 items included in the exploratory factor analyses.

Item name	
compdom	1= Never, 2= Once in a while, 3= Sometimes, 4= Most of the time, 5=Always
compgen	1= Much less competitive than others, 2= A bit less competitive than others, 3=About as competitive as others,<br>4=A bit more competitive than others, 5=Much more competitive than others
riskgen	1=Not at all prepared, 2=Slightly prepared, 3=Moderately prepared, 4= very prepared, 5=Completely prepared
riskfinan	1=Not at all prepared, 2=Slightly prepared, 3=Moderately prepared, 4= very prepared, 5=Completely prepared
riskrecrea	1=Not at all prepared, 2=Slightly prepared, 3=Moderately prepared, 4= very prepared, 5=Completely prepared
indepgen	1=Not at all important, 2= Slightly important, 3=Moderately important, 4=Very important, 5=Extremely important
indephelp	1=Always, 2=Most of the time, 3=Sometimes, 4=Once in a while, 5=Never
indepprob	1=Not at all important, 2= Slightly important, 3=Moderately important, 4=Very important, 5=Extremely important
commother	1= Never, 2= Once in a while, 3= Sometimes, 4= Most of the time, 5=Always
commdecis	1= Never, 2= Once in a while, 3= Sometimes, 4= Most of the time, 5=Always
commfriend	1= Never, 2= Once in a while, 3= Sometimes, 4= Most of the time, 5=Always
commemp	1= Not at all easy, 2=Slightly easy, 3=Moderately easy, 4=Very easy, 5=Extremely easy
exprfriend	1= Never, 2= Once in a while, 3= Sometimes, 4= Most of the time, 5=Always
exprother	1= Not at all easy, 2=Slightly easy, 3=Moderately easy, 4=Very easy, 5=Extremely easy
exprown	1= Not at all easy, 2=Slightly easy, 3=Moderately easy, 4=Very easy, 5=Extremely easy
Exprhelp (reverse scored)	5= Not at all easy, 4=Slightly easy, 3=Moderately easy, 2=Very easy, 1=Extremely easy
careemot	1= Never, 2= Once in a while, 3= Sometimes, 4= Most of the time, 5=Always
carephys	1= Never, 2= Once in a while, 3= Sometimes, 4= Most of the time, 5=Always
carefut	1= Never, 2= Once in a while, 3= Sometimes, 4= Most of the time, 5=Always
timesleep	Numerical estimate of daily time-spending (0-24)
timework	Numerical estimate of daily time-spending (0-24)
timehouse	Numerical estimate of daily time-spending (0-24)
timeleisure	Numerical estimate of daily time-spending (0-24)
timeexc	Numerical estimate of daily time-spending (0-24)
timecare	Numerical estimate of daily time-spending (0-24)
timecom	Numerical estimate of daily time-spending (0-24)
workspeed	1= Never, 2= Once in a while, 3= Sometimes, 4= Most of the time, 5=Always
workrep	1= Never, 2= Once in a while, 3= Sometimes, 4= Most of the time, 5=Always
workemot	1= Never, 2= Once in a while, 3= Sometimes, 4= Most of the time, 5=Always
workphys	1= Never, 2= Once in a while, 3= Sometimes, 4= Most of the time, 5=Always
workrisk	1= Never, 2= Once in a while, 3= Sometimes, 4= Most of the time, 5=Always
workhaz	1= Never, 2= Once in a while, 3= Sometimes, 4= Most of the time, 5=Always
socsupadv	1= Never, 2= Once in a while, 3= Sometimes, 4= Most of the time, 5=Always
socsuplove	1= Never, 2= Once in a while, 3= Sometimes, 4= Most of the time, 5=Always
socsupchores	1= Never, 2= Once in a while, 3= Sometimes, 4= Most of the time, 5=Always
socsuplone	5= Never, 4= Once in a while, 3= Sometimes, 2= Most of the time, 1=Always
discrgen	1= Never, 2= Once in a while, 3= Sometimes, 4= Most of the time, 5=Always
discrhire	1= Never, 2= Once in a while, 3= Sometimes, 4= Most of the time, 5=Always
discred	1= Never, 2= Once in a while, 3= Sometimes, 4= Most of the time, 5=Always
discrmed	1= Never, 2= Once in a while, 3= Sometimes, 4= Most of the time, 5=Always
discrpub	1= Never, 2= Once in a while, 3= Sometimes, 4= Most of the time, 5=Always
discrfam	1= Never, 2= Once in a while, 3= Sometimes, 4= Most of the time, 5=Always
qualfam	1= Terrible, 2=Poor, 3=Average, 4=Good, 5=Excellent
famarg	5= Never, 4= Once in a while, 3= Sometimes, 2= Most of the time, 1=Always

Table S3. Rank of gender characteristics based on occurrences (>2).

Expressiveness and emotionality (37)	Emotional, Emotional, Emotional (+/-), Emotional control, Emotional Control, Emotional expressiveness (+/-), Emotional expressiveness (+/-), Emotional inexpressiveness, Emotionality, Expressiveness, Excitable, Excitable, Restricted emotionality, Restrictive emotion, Restrictive Emotionality, Restrictive emotionality, Inexpressive/Impassive, Concealing emotions, Feelings-easily-hurt (+/-),Fear of Unemotional Relationships, Personal sensitivity, Sensitive, Sentimental, Cries easily – Never cries, Secretive, Moody, Devaluation of emotion, Affection towards children, Affectionate, Restricted affectionate behavior between men, Devotion, Compassionate, Emotional, Sensitive, Affectionate, Easily expresses tender emotion, Affectionate
Empathy, caring and nurturing (37)	Warm, Warm, Warm, Warmth, Friendly, Friendly, Helpful, Helpful (+-) Helpful, Likeable, Kind, Forgiving, Appreciative, Praising, Care for children, Caretaking, Affection towards children, Loves children, Fear of not Being Nurturant, Eager to soothe hurt feelings, Sensitive to other's needs, Nurturance, Home oriented, Domestic, Nurturance, Affiliative concerns, Understanding, Understanding of others, Tender, Empathetic, Tender, Nurturing, Understanding, Sympathetic, Helpful, Tender, Attentive to the needs of others, Loves children, Likes to care for others
Self-reliance and independence (18)	Self-reliance, Self-reliance, Self-reliance, Self-Reliance, Self-sufficient, Indifferent-Needful of others approval (+/-), Independent, Independent, Individualistic, Dependence/Deference, Dependent, Autonomy, Individualism, Self-confident, Self-confident, Confident, Independent, Ability to implement action of one's own accord, Self-supportive
Striving for status, competition, (17)	Achievement/Status, Pursuit of status, Status, Being admired and respected, Competition, Competitive, Competitive, Winning, Ambitious, Gives up easily/Never gives up, Power, Power, superior, Social Ascendancy, Status norm, Power-hungry, Ambitious
Dominance (9)	Dominance, Dominance, Dominance, Dominant, Dominant, Forceful, Forceful, Autocratic, Control
Toughness (9)	Tough, Toughness, Toughness, Toughness, Weak/Strong under pressure, Physical inadequacy, Physical toughness, Not being weak, Toughness norm, Emotional toughness,
Decision-making (11)	Willing to take a stand, Make decisions easily (+/-), Makes decisions easily, Acts as a leader, Foresighted, Defends own beliefs, Hard-headed, Leadership ability, Solution-focused, Leadership abilities, Being a laeder
Aggressiveness (7)	Aggression, Aggressive, Aggressive, Aggressive, Aggression, Aggression, Aggressive
Assertive (6)	Assertive, Assertive, Fear of Behaving Assertively, Outspoken, Frank, Boastful, Ostentatious
Truthful, sincere, reliable (5)	Truthful, Sincere, Sincere, Reliable, Conscientious
Modesty (6)	Modesty, Shy, Modest, Gentle, Timid, Quiet
Risk (5)	Willing to take risks, Danger as exciting, Risk-taking, Enterprising, Orientation Toward Risk
Adaptable, gullible (5)	Adaptable, Gullible, Fickle, Yielding, Adaptive
Worry, fearful (3)	Worrying, Fearful, Need for security (+/-),Overcautious
Active-Passive (4)	Passive-Active, Passivity, Activity-Passivity, Active
Violence (3)	Violence, Violence, Violence as manly
Avoidance of femininity (3)	Avoidance of Femininity, Avoidance of Femininity, Avoiding femininity, Anti-femininity.

Table S4. Search-terms for meta-analyses of existing scales measuring each gender variable.

Gender Variable	Search terms
Caregiving	caregiv* [pubmed]; (caregiv* OR care*) [psychinfo]
Time Use	("time use" OR "time poverty" OR "time deficit" OR "leisure time" OR "unpaid work" OR "domestic work" OR "leisure time physical activity" OR "physical activity" OR "paid work" OR "sleep" OR "sleep time" OR "sleep patterns")
Occupation	("Occupational Segregation" OR "Occupational Preferences" OR "Occupational Patterns" OR "demand-control" OR "emotional labor" OR occupation OR profession OR "Job strain" OR "job hazard*" OR "occupational stratification")
Competitive/striving	("competitive attitude" OR competiti* OR success OR power OR winning OR striving)
Self-reliant/independent	("self-efficacy" OR "self-determination" OR agen* or assertive or adventurous OR "self-reliance" OR "self-reliant")
Sensation-seeking/risk-taking	("risk-taking" OR "sensation-seeking" OR "risky behavior"))
Communal	(collective* OR "communal orientation" OR communal* OR agreeable OR interdependen* OR nurtur* OR "tender-minded*" OR connected)
Empathetic	(empath* OR "emotional intelligence")
Expressive	(emotion* OR express* OR secretive OR extrover* OR introver* OR "restrictive emotionality" OR "restrictive emotional behavior" OR "affectionate behavior" OR affection OR inexpression OR impassive OR passivity OR "emotional control")
Social Support	"social support"
Gender Discrimination	("gender discrimination" OR discriminat*)

Table S5. Health-related items and response options.

Item name	Item	Response categories
General health	In general, would you say your health is...	1= Excellent, 2= Very good, 3=Good, 4=fair, 5=poor (recoded to dummy variable: excellent, very good, good=0, fair, poor=1)
Physical health	Now thinking about your physical health, which includes physical illness and injury, how many days during the past 30 days were your physical health not good?	Number of days (1-30)
Mental health	Now thinking about your mental health, which includes stress, depression, and problems with emotions, how many days during the past 30 days were your mental health not good?	Number of days (1-30)
Activity limitations	During the past 30 days, approximately how many days did poor physical or mental health keep you from doing your usual activities, such as self-care, work, or recreation?	Number of days (1-30)
Smoking	How many cigarettes do you smoke per day, not including e-cigarettes?	Number of cigarettes (recoded to dummy variable: not smoking=0, smoking=1)
Vaping	How many times do you vape or use an e-cigarette product per day?	Number of times vaping (recoded to dummy variable: not vaping=0, vaping=1)
Binge drinking	Men: In the past 3 months, how often have you had 5 or more drinks on one occasion?<br>Women: In the past 3 months, how often have you had 4 or more drinks on one occasion?	1=Never, 2=Less than monthly, 3=Monthly, 4=Weekly, 5=Daily or almost daily (recoded to unisex dummy variable: binge drinking less than monthly=0, binge drinking monthly, weekly or daily/almost daily=1)
BMI	[BMI is calculated based on self-reported height and weight]	BMI recoded to Dummy: BMI ≤25 = 0, BMI>25=1

Table S6. Demographic items and response options.

Item name	Item	Response categories
Sex	What was your birth sex?	1=Male, 2=female, 3=intersex, 4=other (please specify), 5=prefer not to state
Gender (man)	What is your gender (please select all that apply)	1=Man
Gender (woman)	What is your gender (please select all that apply)	1=Woman
Gender (Gender fluid/Non-binary)	What is your gender (please select all that apply)	1= Gender fluid/Non-binary/Gender "other"
Gender (other)	What is your gender (please select all that apply)	1=Other
Ethnicity (White)	What category describes you (you may choose more than one)	1=White
Ethnicity (Hispanic, Latino, Spanish)	What category describes you (you may choose more than one)	1= Hispanic, Latino, Spanish
Ethnicity (Black, African, American)	What category describes you (you may choose more than one)	1=Black, African American
Ethnicity (Asian)	What category describes you (you may choose more than one)	1=Asian
Ethnicity (Native American or Alaska Native)	What category describes you (you may choose more than one)	1= Native American, Alaska Native
Ethnicity (Middle Eastern or North American)	What category describes you (you may choose more than one)	1=Middle Eastern, North African
Ethnicity (Native Hawaiian or Pacific Islander)	What category describes you (you may choose more than one)	1= Native Hawaiian, Pacific Islander
Ethnicity (Other)	What category describes you (you may choose more than one)	1=Other
Birth year	In what year were you born?	1= open numeric response option (1900-2000)
Education level	What is the highest degree or level of school you have completed? If currently enrolled, please report the highest degree received.	1=No Schooling completed (…), 11=Doctorate degree
Personal income	What was your income last calendar year? Please combine all incomes. "Incomes" include wages, salaries, small business earnings, social security, armed forces pay, special cash bonuses and subsistence allowances.	1= Less than $10.000 (…). 13= More than $200.000

Table S7. Recoding of ten variables to allow for the largest possible sample in the EFA.

**Caregiving items**<br>The group of current caregivers in sample 1 consists of 605 respondents. To utilize the largest possible sample in the EFA, we recoded the following three items so that people not currently taking care of someone in need were ascribed the value 1 (same procedure was used in samples 2 and 3)
**careemot**. In the past year, how often did you feel emotionally exhausted because of your caretaking responsibilities? **1= Never**, 2= Once in a while, 3= Sometimes. 4= Most of the time. 5=Always
**carephys.** In the past year, how often did you feel physically exhausted because of your caretaking responsibilities? **1= Never**, 2= Once in a while, 3= Sometimes, 4= Most of the time, 5=Always
**carefut.** In the past year, how often have your caretaking responsibilities caused you to worry about the future?<br>**1= Never**, 2= Once in a while, 3= Sometimes, 4= Most of the time, 5=Always
**Work items**<br>The group of current employees in sample 1 consists of 1462 respondents. To utilize the largest possible sample in the EFA. we recoded the following six items so that people not currently employed were ascribed the value 1 (same procedure was used in sample 2)
**workspeed.** How often does your job require working fast? **1= Never**, 2= Once in a while, 3= Sometimes, 4= Most of the time, 5=Always
**workrep.** How often does your job involve repetitive tasks? **1= Never**, 2= Once in a while, 3= Sometimes, 4= Most of the time, 5=Always
**workemot.** How often do you feel emotionally exhausted from your work activities? **1= Never**, 2= Once in a while, 3= Sometimes, 4= Most of the time, 5=Always
**workphys.** How often do you feel physically exhausted from your work activities? **1= Never**, 2= Once in a while, 3= Sometimes, 4= Most of the time, 5=Always
**workrisk.** How often does your job involve risk of harm or injury? **1= Never**, 2= Once in a while, 3= Sometimes, 4= Most of the time, 5=Always
**workhaz.** How often does your job involve hazards, such as smoke, heat, noise, or chemicals? **1= Never**, 2= Once in a while, 3= Sometimes, 4= Most of the time, 5=Always
**Discrimination**<br>258 respondents have never worked and were not asked the question about perceived discrimination when getting hired (discrhire) in sample 1. To utilize the largest possible sample in the EFA, we recoded this item so that respondents that have never worked were ascribed the value 1 (all respondents were asked this question in sample 2, so no recoding was necessary).
**discrhire.** Because of your gender, how often have you felt discriminated against, when getting hired? **1= Never**, 2= Once in a while, 3= Sometimes, 4= Most of the time, 5=Always

Table S8. Exploratory Factor Analysis (Full factor model).

	1	*2*	*3*	*4*	*5*	*6*	*7*
discrgen	**0.853**	-0.043	-0.060	-0.077	0.093	-0.003	0.093
discrpub	**0.840**	0.002	-0.056	-0.063	0.055	0.012	0.035
discred	**0.811**	-0.031	-0.049	0.033	-0.054	0.040	-0.007
discrmed	**0.753**	0.012	0.033	-0.010	-0.050	0.047	-0.008
discrhire	**0.728**	0.082	0.021	0.009	-0.039	0.066	0.070
discrfam	**0.624**	-0.019	0.047	0.002	-0.003	-0.128	-0.042
workspeed	-0.041	**0.831**	-0.006	-0.046	0.084	0.053	0.068
workphys	0.017	**0.816**	0.020	-0.014	-0.003	-0.086	-0.066
workemot	0.052	**0.800**	0.019	-0.086	0.018	-0.058	-0.019
workrep	-0.031	**0.799**	0.003	-0.065	0.040	0.014	0.039
timework	-0.120	**0.582**	-0.155	-0.024	0.016	0.071	0.122
workrisk	0.126	**0.493**	0.039	0.182	-0.141	-0.014	-0.101
workhaz	0.116	**0.427**	0.026	0.167	-0.118	-0.042	-0.102
timeleisure	0.042	-0.358	-0.343	0.015	-0.034	-0.127	-0.128
timehouse	0.069	-0.230	0.130	0.056	0.060	0.019	0.068
timecom	-0.003	0.162	-0.061	0.150	0.058	-0.069	0.014
carephys	-0.010	-0.001	**0.938**	0.007	-0.020	0.008	-0.025
careemot	-0.003	-0.006	**0.933**	0.001	0.015	-0.027	-0.015
carefut	-0.018	-0.006	**0.903**	0.003	0.000	-0.021	-0.011
timecare	-0.006	-0.126	**0.703**	-0.080	0.055	0.069	0.068
timesleep	0.017	-0.042	-0.175	-0.047	-0.001	0.053	-0.005
riskgen	0.029	-0.047	0.000	**0.754**	-0.050	0.113	0.123
riskfinan	-0.031	0.057	0.025	**0.670**	-0.149	0.109	0.028
riskrecrea	0.023	0.010	-0.003	**0.593**	-0.028	0.069	0.051
compgen	-0.125	-0.031	0.006	**0.528**	0.077	-0.142	0.088
compdom	-0.021	-0.001	0.026	**0.489**	0.054	-0.226	-0.014
timeexc	0.019	-0.072	-0.120	0.243	0.044	-0.008	0.013
commfriend	0.060	0.004	-0.011	-0.006	**0.667**	-0.091	0.093
commemp	0.003	0.012	-0.003	0.024	**0.587**	-0.086	0.201
exprfriend	-0.010	0.030	-0.034	0.051	**0.522**	-0.034	-0.295
commdecis	-0.032	-0.035	0.124	-0.170	**0.466**	0.037	0.049
exprother	-0.049	0.008	0.001	0.101	**0.434**	0.199	-0.110
exprown	-0.042	-0.006	-0.016	0.022	0.367	0.252	0.137
socsuplone	-0.064	0.002	-0.057	-0.060	-0.143	**0.709**	0.064
socsuplove	0.098	-0.030	0.058	-0.073	0.188	**0.619**	-0.063
socsupchores	0.112	0.010	0.087	-0.004	-0.028	**0.510**	-0.191
qualfam	-0.089	-0.043	-0.037	0.070	0.101	0.386	-0.043
commother	0.019	0.016	0.004	-0.048	0.240	-0.386	-0.198
socsupadv	0.059	-0.011	-0.033	0.000	0.235	0.296	-0.233
qualfamarg	-0.167	0.007	-0.100	-0.226	0.001	0.263	0.142
indepprob	0.051	-0.001	0.039	0.193	0.194	-0.006	**0.706**
indepgen	0.052	0.034	-0.013	0.157	0.203	0.030	**0.679**
indephelp	0.002	-0.013	-0.011	-0.049	-0.133	-0.044	**0.568**
Exprhelp (reverse scored)	0.048	-0.038	0.053	-0.196	-0.132	-0.207	**0.489**

Note: N=1999. KMO: 0.826; Bartlett's Test of Sphericity: $\chi 2$ = 36185.003, df = 946, $p < 0.001$, Extraction method: Principal Axis Factoring. Rotation Method: Promax w. Kaiser Normalization. Rotation converged in 8 iterations. Total Variance Explained: 50.8%. Item loadings from the Pattern Matrix. The total variance explained by each factor was: Factor 1= 12.5%, Factor 2= 9.1%, Factor 3= 8.4%, Factor 4= 7.4%, Factor 5= 5.4%, Factor 6= 4.4%, Factor 7= 3.7%.

Table S9. Communalities and unique variances for exploratory factor analysis presented in Table S8.

**Communalities**			**Unique Variance**
	Initial	Extraction	
careemot	0.85	0.88	0.12
carefut	0.79	0.81	0.19
carephys	0.84	0.87	0.13
commdecis	0.22	0.22	0.78
commemp	0.26	0.30	0.7
commfriend	0.33	0.40	0.6
commother	0.26	0.21	0.79
compdom	0.40	0.26	0.74
compgen	0.39	0.28	0.72
discred	0.56	0.62	0.38
discrfam	0.43	0.46	0.54
discrgen	0.67	0.72	0.28
discrhire	0.51	0.52	0.48
discrmed	0.50	0.55	0.45
discrpub	0.66	0.69	0.31
exprfriend	0.42	0.47	0.53
exprhelp (reverse coded)	0.48	0.47	0.53
exprother	0.43	0.37	0.63
exprown	0.30	0.24	0.76
indepgen	0.41	0.44	0.56
indephelp	0.36	0.42	0.58
indepprob	0.43	0.48	0.52
qualfam	0.24	0.23	0.77
qualfamarg	0.20	0.20	0.8
riskfinan	0.40	0.46	0.54
riskgen	0.48	0.57	0.43
riskrecrea	0.35	0.36	0.64
socsupadv	0.33	0.30	0.7
socsupchores	0.37	0.31	0.69
socsuplone	0.37	0.49	0.51
socsuplove	0.50	0.49	0.51
timecare	0.54	0.51	0.49
timecom	0.15	0.07	0.93
timeexc	0.26	0.07	0.93
timehouse	0.24	0.07	0.93
timeleisure	0.45	0.30	0.7
timesleep	0.10	0.04	0.96
timework	0.50	0.37	0.63
workemot	0.60	0.63	0.37
workhaz	0.49	0.28	0.72
workphys	0.63	0.67	0.33
workrep	0.61	0.62	0.38
workrisk	0.54	0.36	0.64
workspeed	0.67	0.69	0.31

Table S10. Exploratory Factor Analysis (Full factor model), Oblimin rotation.

	1	*2*	*3*	*4*	*5*	*6*	*7*
discrgen	**0.853**	-0.034	-0.007	0.072	-0.081	0.086	0.089
discrpub	**0.841**	0.009	0.009	0.065	-0.066	0.033	0.059
discred	**0.806**	-0.022	0.039	0.058	0.023	-0.002	-0.040
discrmed	**0.749**	0.016	0.045	-0.025	-0.018	-0.003	-0.035
discrhire	**0.724**	0.088	0.063	-0.017	0.001	0.073	-0.025
discrfam	**0.633**	-0.017	-0.120	-0.043	-0.011	-0.036	-0.003
workspeed	-0.010	**0.821**	0.062	-0.037	-0.026	0.071	0.101
workphys	0.055	**0.801**	-0.070	-0.064	-0.004	-0.047	0.018
workrep	-0.001	**0.786**	0.021	-0.043	-0.049	0.049	0.059
workemot	0.086	**0.783**	-0.049	-0.058	-0.074	-0.002	0.037
timework	-0.106	**0.577**	0.071	0.126	-0.010	0.128	0.028
workrisk	0.144	**0.490**	0.003	-0.070	0.177	-0.083	-0.110
workhaz	0.133	**0.424**	-0.025	-0.054	0.162	-0.086	-0.093
timehouse	0.063	-0.222	0.022	-0.120	0.050	0.051	0.049
timecom	0.012	0.169	-0.051	0.043	0.149	0.007	0.054
socsuplone	-0.115	0.011	**0.669**	0.072	-0.033	0.055	-0.089
socsuplove	0.074	-0.017	**0.603**	-0.049	-0.031	-0.106	0.223
socsupchores	0.088	0.016	**0.497**	-0.081	0.021	-0.206	0.022
qualfam	-0.104	-0.029	0.383	0.037	0.094	-0.072	0.122
commother	0.060	0.009	-0.358	-0.015	-0.046	-0.204	0.209
socsupadv	0.063	-0.001	0.303	0.030	0.034	-0.268	0.253
qualfamarg	-0.189	0.003	0.231	0.113	-0.209	0.140	0.008
carephys	-0.002	-0.014	0.018	**-0.937**	-0.007	-0.039	-0.009
careemot	0.008	-0.019	-0.015	**-0.933**	-0.013	-0.032	0.021
carefut	-0.009	-0.019	-0.010	**-0.903**	-0.011	-0.027	0.006
timecare	-0.008	-0.134	0.067	**-0.692**	-0.086	0.047	0.054
timeleisure	0.034	-0.352	-0.128	0.360	0.009	-0.117	-0.047
timesleep	0.011	-0.040	0.045	0.180	-0.041	-0.003	0.000
riskgen	0.026	-0.006	0.158	-0.028	**0.732**	0.090	-0.040
riskfinan	-0.035	0.087	0.147	-0.054	**0.649**	0.012	-0.127
riskrecrea	0.025	0.041	0.108	-0.023	**0.578**	0.026	-0.019
compgen	-0.108	-0.005	-0.097	-0.033	**0.511**	0.060	0.060
compdom	0.002	0.020	-0.179	-0.053	**0.469**	-0.032	0.039
timeexc	0.022	-0.055	0.009	0.112	0.239	-0.001	0.039
indepprob	0.047	0.024	-0.001	-0.046	0.178	**0.663**	0.146
indepgen	0.047	0.058	0.032	0.006	0.146	**0.637**	0.158
indephelp	-0.020	-0.011	-0.069	0.021	-0.074	**0.577**	-0.158
exprhelp_reverse	0.034	-0.048	-0.234	-0.037	-0.224	**0.511**	-0.165
commfriend	0.102	0.021	-0.058	-0.004	0.026	0.019	**0.613**
commemp	0.037	0.030	-0.058	-0.012	0.048	0.133	**0.533**
exprfriend	0.032	0.041	0.005	0.014	0.088	-0.349	**0.503**
commdecis	-0.011	-0.031	0.045	-0.125	-0.140	-0.003	**0.434**
exprother	-0.030	0.027	0.224	-0.015	0.137	-0.170	**0.426**
exprown	-0.038	0.012	0.259	0.011	0.052	0.082	0.353

Note: N=1999. KMO: 0.826; Bartlett's Test of Sphericity: $\chi 2$ = 36185.003, df = 946, $p < 0.001$, Extraction method: Principal Axis Factoring. Rotation Method: Direct Oblimin. Rotation converged in 8 iterations. Total Variance Explained: 50.8%. Item loadings from the Pattern Matrix. The total variance explained by each factor was: Factor 1= 12.5%, Factor 2= 9.1%, Factor 3= 8.36%, Factor 4= 7.4%, Factor 5= 5.4%, Factor 6= 4.4%, Factor 7= 3.7%.

Table S11. Exploratory Factor Analysis (Full factor model), Varimax rotation.

	1	*2*	*3*	*4*	*5*	*6*	*7*
discrgen	**0.832**	-0.006	0.001	-0.026	0.131	-0.070	0.041
discrpub	**0.822**	0.038	0.005	-0.011	0.110	-0.054	-0.009
discred	**0.784**	0.016	0.001	0.062	0.026	-0.028	-0.039
discrmed	**0.732**	0.050	0.079	0.024	0.030	-0.020	-0.036
discrhire	**0.703**	0.123	0.073	0.050	0.033	-0.001	0.041
discrfam	**0.644**	0.014	0.092	0.022	0.038	-0.169	-0.062
qualfamarg	-0.245	-0.037	-0.130	-0.212	-0.023	0.234	0.142
workspeed	0.002	**0.816**	0.060	0.066	0.103	0.066	0.077
workphys	0.087	**0.802**	0.085	0.079	0.027	-0.073	-0.036
workrep	0.013	**0.778**	0.063	0.035	0.058	0.020	0.057
workemot	0.109	**0.778**	0.083	0.013	0.039	-0.057	0.006
timework	-0.121	**0.563**	-0.119	0.044	0.007	0.077	0.136
workrisk	0.164	**0.518**	0.082	0.220	-0.063	-0.013	-0.076
workhaz	0.155	**0.449**	0.066	0.199	-0.054	-0.038	-0.080
timeleisure	0.018	-0.367	-0.365	-0.035	-0.073	-0.121	-0.123
timehouse	0.069	-0.205	0.125	0.038	0.063	0.020	0.043
timecom	0.029	0.185	-0.032	0.168	0.059	-0.041	0.006
carephys	0.075	0.032	**0.927**	0.001	0.064	0.000	-0.025
careemot	0.090	0.027	**0.927**	-0.002	0.088	-0.031	-0.020
carefut	0.069	0.025	**0.895**	-0.003	0.071	-0.026	-0.014
timecare	0.035	-0.108	**0.686**	-0.084	0.096	0.054	0.051
timesleep	-0.013	-0.054	-0.180	-0.045	-0.011	0.046	-0.007
riskgen	0.041	0.085	0.033	**0.722**	0.043	0.170	0.084
riskfinan	-0.020	0.165	0.047	**0.637**	-0.046	0.155	0.016
riskrecrea	0.044	0.112	0.028	**0.575**	0.049	0.120	0.021
compgen	-0.063	0.055	0.037	**0.502**	0.084	-0.067	0.061
compdom	0.060	0.080	0.064	**0.466**	0.070	-0.156	-0.032
timeexc	0.027	-0.031	-0.105	0.234	0.056	0.022	-0.008
commfriend	0.156	0.032	0.062	0.092	**0.604**	-0.001	-0.015
exprfriend	0.100	0.050	0.022	0.139	**0.539**	0.072	-0.373
commemp	0.080	0.042	0.061	0.105	**0.512**	-0.008	0.104
exprother	-0.002	0.040	0.039	0.179	**0.473**	0.279	-0.193
commdecis	0.019	-0.041	0.153	-0.098	**0.426**	0.084	-0.023
exprown	-0.045	0.017	0.011	0.088	0.369	0.294	0.060
socsuplone	-0.213	-0.006	-0.099	-0.038	-0.034	**0.658**	0.051
socsuplove	0.028	-0.019	0.058	0.001	0.304	**0.614**	-0.130
socsupchores	0.052	0.021	0.076	0.036	0.113	**0.492**	-0.218
qualfam	-0.133	-0.027	-0.044	0.100	0.169	0.405	-0.081
socsupadv	0.064	0.000	-0.016	0.063	0.315	0.332	-0.287
commother	0.132	0.008	0.038	-0.027	0.186	-0.330	-0.209
indepprob	0.024	0.059	0.076	0.200	0.101	-0.010	**0.648**
indepgen	0.018	0.086	0.024	0.174	0.113	0.025	**0.621**
indephelp	-0.072	-0.015	-0.023	-0.087	-0.231	-0.107	**0.582**
exprhelp_reverse	0.002	-0.064	0.038	-0.238	-0.256	-0.279	**0.518**

Note: N=1999. KMO: 0.826; Bartlett's Test of Sphericity: $\chi 2$ = 36185.003, df = 946, $p < 0.001$, Extraction method: Principal Axis Factoring. Rotation Method: Varimax. Rotation converged in 8 iterations. Total Variance Explained: 50.8%. Item loadings from the Pattern Matrix. The total variance explained by each factor was: Factor 1= 12.5%, Factor 2= 9.1%, Factor 3= 8.36%, Factor 4= 7.4%, Factor 5= 5.4%, Factor 6= 4.4%, Factor 7= 3.7%.

Table S12. Exploratory Factor Analysis (Full factor model), Equamax rotation.

	1	*2*	*3*	*4*	*5*	*6*	*7*
discrgen	**0.832**	-0.006	0.001	-0.026	0.131	-0.070	0.041
discrpub	**0.822**	0.038	0.005	-0.011	0.110	-0.054	-0.009
discred	**0.784**	0.016	0.001	0.062	0.026	-0.028	-0.039
discrmed	**0.732**	0.050	0.079	0.024	0.030	-0.020	-0.036
discrhire	**0.703**	0.123	0.073	0.050	0.033	-0.001	0.041
discrfam	**0.644**	0.014	0.092	0.022	0.038	-0.169	-0.062
qualfamarg	-0.245	-0.037	-0.130	-0.212	-0.023	0.234	0.142
workspeed	0.002	**0.816**	0.060	0.066	0.103	0.066	0.077
workphys	0.087	**0.802**	0.085	0.079	0.027	-0.073	-0.036
workrep	0.013	**0.778**	0.063	0.035	0.058	0.020	0.057
workemot	0.109	**0.778**	0.083	0.013	0.039	-0.057	0.006
timework	-0.121	**0.563**	-0.119	0.044	0.007	0.077	0.136
workrisk	0.164	**0.518**	0.082	0.220	-0.063	-0.013	-0.076
workhaz	0.155	**0.449**	0.066	0.199	-0.054	-0.038	-0.080
timeleisure	0.018	-0.367	-0.365	-0.035	-0.073	-0.121	-0.123
timehouse	0.069	-0.205	0.125	0.038	0.063	0.020	0.043
timecom	0.029	0.185	-0.032	0.168	0.059	-0.041	0.006
carephys	0.075	0.032	**0.927**	0.001	0.064	0.000	-0.025
careemot	0.090	0.027	**0.927**	-0.002	0.088	-0.031	-0.020
carefut	0.069	0.025	**0.895**	-0.003	0.071	-0.026	-0.014
timecare	0.035	-0.108	**0.686**	-0.084	0.096	0.054	0.051
timesleep	-0.013	-0.054	-0.180	-0.045	-0.011	0.046	-0.007
riskgen	0.041	0.085	0.033	**0.722**	0.043	0.170	0.084
riskfinan	-0.020	0.165	0.047	**0.637**	-0.046	0.155	0.016
riskrecrea	0.044	0.112	0.028	**0.575**	0.049	0.120	0.021
compgen	-0.063	0.055	0.037	**0.502**	0.084	-0.067	0.061
compdom	0.060	0.080	0.064	**0.466**	0.070	-0.156	-0.032
timeexc	0.027	-0.031	-0.105	0.234	0.056	0.022	-0.008
commfriend	0.156	0.032	0.062	0.092	**0.604**	-0.001	-0.015
exprfriend	0.100	0.050	0.022	0.139	**0.539**	0.072	-0.373
commemp	0.080	0.042	0.061	0.105	**0.512**	-0.008	0.104
exprother	-0.002	0.040	0.039	0.179	**0.473**	0.279	-0.193
commdecis	0.019	-0.041	0.153	-0.098	**0.426**	0.084	-0.023
exprown	-0.045	0.017	0.011	0.088	0.369	0.294	0.060
socsuplone	-0.213	-0.006	-0.099	-0.038	-0.034	**0.658**	0.051
socsuplove	0.028	-0.019	0.058	0.001	0.304	**0.614**	-0.130
socsupchores	0.052	0.021	0.076	0.036	0.113	**0.492**	-0.218
qualfam	-0.133	-0.027	-0.044	0.100	0.169	0.405	-0.081
socsupadv	0.064	0.000	-0.016	0.063	0.315	0.332	-0.287
commother	0.132	0.008	0.038	-0.027	0.186	-0.330	-0.209
indepprob	0.024	0.059	0.076	0.200	0.101	-0.010	**0.648**
indepgen	0.018	0.086	0.024	0.174	0.113	0.025	**0.621**
indephelp	-0.072	-0.015	-0.023	-0.087	-0.231	-0.107	**0.582**
exprhelp_reverse	0.002	-0.064	0.038	-0.238	-0.256	-0.279	**0.518**

Note: N=1999. KMO: 0.826; Bartlett's Test of Sphericity: $\chi 2 = 36185.003$, df = 946, $p < 0.001$, Extraction method: Principal Axis Factoring. Rotation Method: Equamax. Rotation converged in 8 iterations. Total Variance Explained: 50.8%. Item loadings from the Pattern Matrix. The total variance explained by each factor was: Factor 1= 12.5%, Factor 2= 9.1%, Factor 3= 8.36%, Factor 4= 7.4%, Factor 5= 5.4%, Factor 6= 4.4%, Factor 7= 3.7%.

Table S13. Exploratory Factor Analysis (Full factor model), Quartimax rotation.

	1	*2*	*3*	*4*	*5*	*6*	*7*
discrgen	**0.833**	-0.004	-0.001	-0.026	0.127	-0.065	0.047
discrpub	**0.823**	0.040	0.003	-0.011	0.107	-0.049	-0.004
discred	**0.784**	0.018	-0.001	0.062	0.024	-0.022	-0.035
discrmed	**0.733**	0.052	0.077	0.023	0.028	-0.015	-0.032
discrhire	**0.703**	0.126	0.070	0.049	0.030	0.003	0.045
discrfam	**0.646**	0.016	0.090	0.022	0.035	-0.164	-0.059
qualfamarg	-0.248	-0.040	-0.129	-0.212	-0.021	0.232	0.141
workspeed	0.000	**0.817**	0.056	0.060	0.102	0.065	0.078
workphys	0.086	**0.803**	0.082	0.072	0.026	-0.072	-0.037
workrep	0.011	**0.779**	0.060	0.029	0.056	0.020	0.057
workemot	0.107	**0.779**	0.079	0.006	0.037	-0.056	0.006
timework	-0.124	**0.563**	-0.121	0.039	0.005	0.075	0.135
workrisk	0.163	**0.520**	0.079	0.216	-0.064	-0.011	-0.077
workhaz	0.155	**0.451**	0.063	0.195	-0.054	-0.036	-0.081
timeleisure	0.019	-0.369	-0.363	-0.032	-0.072	-0.120	-0.124
timehouse	0.069	-0.204	0.125	0.040	0.062	0.019	0.044
timecom	0.029	0.186	-0.033	0.167	0.057	-0.042	0.007
carephys	0.077	0.036	**0.927**	0.001	0.064	0.001	-0.025
careemot	0.093	0.032	**0.927**	-0.002	0.088	-0.030	-0.019
carefut	0.072	0.029	**0.894**	-0.003	0.071	-0.025	-0.013
timecare	0.036	-0.106	**0.687**	-0.082	0.096	0.054	0.053
timesleep	-0.013	-0.055	-0.180	-0.045	-0.010	0.046	-0.007
riskgen	0.040	0.091	0.033	**0.721**	0.041	0.168	0.086
riskfinan	-0.021	0.170	0.046	**0.636**	-0.046	0.154	0.017
riskrecrea	0.043	0.117	0.028	**0.575**	0.049	0.119	0.023
compgen	-0.062	0.059	0.037	**0.502**	0.080	-0.069	0.062
compdom	0.062	0.084	0.063	**0.465**	0.067	-0.157	-0.031
timeexc	0.027	-0.029	-0.105	0.234	0.055	0.021	-0.006
commfriend	0.158	0.035	0.061	0.093	**0.604**	-0.008	-0.003
exprfriend	0.103	0.051	0.020	0.140	**0.546**	0.067	-0.362
commemp	0.081	0.045	0.060	0.106	**0.509**	-0.015	0.114
exprother	-0.001	0.042	0.038	0.181	**0.479**	0.274	-0.183
commdecis	0.021	-0.040	0.153	-0.096	**0.428**	0.079	-0.015
exprown	-0.047	0.018	0.011	0.090	0.372	0.289	0.068
socsuplone	-0.218	-0.008	-0.099	-0.037	-0.026	**0.656**	0.052
socsuplove	0.025	-0.019	0.057	0.003	0.314	**0.611**	-0.122
socsupchores	0.050	0.022	0.075	0.037	0.123	**0.492**	-0.213
qualfam	-0.135	-0.027	-0.044	0.102	0.175	0.402	-0.077
commother	0.137	0.008	0.038	-0.027	0.185	-0.330	-0.206
socsupadv	0.064	0.001	-0.017	0.065	0.324	0.330	-0.279
indepprob	0.021	0.062	0.076	0.199	0.089	-0.014	**0.650**
indepgen	0.014	0.089	0.025	0.173	0.102	0.020	**0.623**
indephelp	-0.075	-0.015	-0.022	-0.089	-0.242	-0.107	**0.577**
exprhelp_reverse	0.000	-0.065	0.039	-0.240	-0.268	-0.277	**0.512**

Note: N=1999. KMO: 0.826; Bartlett's Test of Sphericity: $\chi2$ = 36185.003, df = 946, $p < 0.001$, Extraction method: Principal Axis Factoring. Rotation Method: Quartimax. Rotation converged in 8 iterations. Total Variance Explained: 50.8%. Item loadings from the Pattern Matrix. The total variance explained by each factor was: Factor 1= 12.5%, Factor 2= 9.1%, Factor 3= 8.36%, Factor 4= 7.4%, Factor 5= 5.4%, Factor 6= 4.4%, Factor 7= 3.7%.

Table S14. Factor loadings for CFA Models 1 and 2 in sample 1.

**Item**		Model 1	CI Lower	CI Upper	Model 2	CI Lower	CI Upper
discrgen	Factor 1	0.85	.824	.874	0.85	0.824	0.874
discrhire	Factor 1	0.708	.673	.739	0.707	0.673	0.738
discred	Factor 1	0.776	.751	.801	0.776	0.751	0.801
discrmed	Factor 1	0.727	.695	.757	0.727	0.695	0.756
discrpub	Factor 1	0.846	.826	.865	0.846	0.826	0.865
discrfam	Factor 1	0.648	.609	.684	0.648	0.609	0.684
workspeed	Factor 2	0.829	.802	.852	0.864	0.843	0.883
workrep	Factor 2	0.8	.775	.823	0.821	0.799	0.841
workemot	Factor 2	0.8	.777	.823	0.792	0.769	0.815
workphys	Factor 2	0.816	.795	.837	0.775	0.752	0.798
timework	Factor 2	0.501	.458	.543	0.52	0.478	0.56
workrisk	Factor 2	0.532	.500	.565			
workhaz	Factor 2	0.459	.424	.494			
carephys	Factor 3	0.941	.929	.953	0.941	0.929	0.953
careemot	Factor 3	0.953	.938	.967	0.953	0.938	0.967
carefut	Factor 3	0.91	.894	.924	0.91	0.894	0.924
timecare	Factor 3	0.644	.598	.686	0.643	0.598	0.686
riskgen	Factor 4	0.802	.765	.835	0.836	0.799	0.874
riskfinan	Factor 4	0.676	.643	.708	0.672	0.636	0.708
riskrecrea	Factor 4	0.647	.611	.682	0.649	0.611	0.685
compgen	Factor 4	0.451	.397	.504			
compdom	Factor 4	0.396	.336	.456			
socsuplone	Factor 5	0.421	.364	.473			
socsuplove	Factor 5	0.929	.867	.996	0.754	0.727	0.779
socsupchores	Factor 5	0.59	.537	.643	0.726	0.703	0.749
commfriend	Factor 6	0.565	.501	.619	0.507	0.457	0.556
commemp	Factor 6	0.476	.410	.528			
exprfriend	Factor 6	0.595	.546	.648	0.754	0.705	0.81
exprother	Factor 6	0.648	.593	.700	0.589	0.542	0.635
commdecis	Factor 6	0.361	.299	.420			
exprown	Factor 6	0.474	.420	.525			
indepprob	Factor 7	0.804	.722	.846	0.79	0.712	0.872
indepgen	Factor 7	0.719	.655	.757	0.762	0.683	0.844
indephelp	Factor 7	0.434	.367	.549			
Exprhelp (reverse scored)	Factor 7	0.368	.295	.500			

Note: The 95% confidence intervals were based on 2000 boostrap samples

Table S15. Factor loadings for CFA Samples 2 and 3 (Configural invariance).

**Item**		Sample 2	CI Lower	CI Upper	Sample 3	CI Lower	CI Upper
discrgen	Factor 1	0.492	0.445	0.533	0.86	0.799	0.914
discrhire	Factor 1	0.526	0.47	0.579	0.537	0.456	0.614
discred	Factor 1	0.566	0.517	0.613			
discrmed	Factor 1	0.582	0.554	0.606	0.608	0.526	0.677
discrpub	Factor 1	0.62	0.578	0.66	0.845	0.794	0.894
discrfam	Factor 1	0.62	0.582	0.657	0.577	0.494	0.66
workspeed	Factor 2	0.625	0.579	0.667	0.905	0.874	0.934
workrep	Factor 2	0.679	0.64	0.714	0.865	0.827	0.897
workemot	Factor 2	0.693	0.655	0.727	0.872	0.835	0.907
workphys	Factor 2	0.717	0.638	0.802	0.825	0.786	0.865
timework	Factor 2	0.726	0.697	0.754	0.619	0.542	0.691
carephys	Factor 3	0.746	0.714	0.775	0.933	0.901	0.96
careemot	Factor 3	0.76	0.734	0.783	0.942	0.914	0.967
carefut	Factor 3	0.76	0.707	0.818	0.905	0.865	0.94
timecare	Factor 3	0.789	0.761	0.813	0.526	0.413	0.646
riskgen	Factor 4	0.791	0.765	0.817	0.82	0.667	1,006
riskfinan	Factor 4	0.837	0.746	0.935	0.575	0.456	0.682
riskrecrea	Factor 4	0.84	0.811	0.865	0.501	0.374	0.609
socsuplove	Factor 5	0.842	0.804	0.879	0.909	0.805	1,022
socsupchores	Factor 5	0.85	0.824	0.873	0.607	0.538	0.653
commfriend	Factor 6	0.86	0.841	0.878	0.521	0.373	0.642
exprfriend	Factor 6	0.892	0.873	0.911	0.775	0.62	0.987
exprother	Factor 6	0.947	0.936	0.958	0.541	0.41	0.667
indepprob	Factor 7	0.971	0.959	0.98	0.768	0.555	1,147
indepgen	Factor 7	0.973	0.924	1,026	0.656	0.426	0.873

Note: The 95% confidence intervals were based on 2000 boostrap samples

Table S16. Factor loadings for CFA Samples 2 and 3 (Metric invariance, 24 items).

**Item**		Sample 2	CI Lower	CI Upper	Sample 3	CI Lower	CI Upper
discrgen	Factor 1	0.86	0.835	0.883	0.843	0.802	0.88
discrhire	Factor 1	0.705	0.672	0.734	0.66	0.625	0.696
discred							
discrmed	Factor 1	0.671	0.636	0.703	0.609	0.556	0.658
discrpub	Factor 1	0.862	0.84	0.88	0.815	0.773	0.853
discrfam	Factor 1	0.621	0.58	0.66	0.597	0.542	0.653
workspeed	Factor 2	0.85	0.826	0.87	0.902	0.875	0.927
workrep	Factor 2	0.782	0.758	0.806	0.874	0.842	0.904
workemot	Factor 2	0.794	0.77	0.817	0.871	0.837	0.902
workphys	Factor 2	0.757	0.733	0.779	0.834	0.803	0.866
timework	Factor 2	0.522	0.484	0.558	0.471	0.423	0.523
carephys	Factor 3	0.947	0.936	0.957	0.937	0.91	0.961
careemot	Factor 3	0.97	0.959	0.98	0.944	0.918	0.967
carefut	Factor 3	0.894	0.876	0.912	0.895	0.854	0.929
timecare	Factor 3	0.625	0.587	0.667	0.471	0.384	0.575
riskgen	Factor 4	0.847	0.81	0.885	0.758	0.678	0.833
riskfinan	Factor 4	0.675	0.639	0.711	0.604	0.545	0.662
riskrecrea	Factor 4	0.614	0.577	0.649	0.551	0.493	0.609
socsuplove	Factor 5	0.964	0.915	1,014	0.946	0.875	1,017
socsupchores	Factor 5	0.588	0.561	0.611	0.581	0.541	0.614
commfriend	Factor 6	0.529	0.476	0.582	0.515	0.452	0.583
exprfriend	Factor 6	0.763	0.707	0.817	0.754	0.666	0.836
exprother	Factor 6	0.56	0.512	0.608	0.572	0.506	0.631
indepprob	Factor 7	0.845	0.76	0.938	0.724	0.625	0.839
indepgen	Factor 7	0.71	0.636	0.785	0.696	0.605	0.785

Note: The 95% confidence intervals were based on 2000 boostrap samples

Table S17. Factor loadings for final CFA in samples 2 and 3 (Scalar invariance, 24 items).

**Item**		Sample 2	CI Lower	CI Upper	Sample 3	CI Lower	CI Upper
discrgen	Factor 1	0.859	0.835	0.883	0.837	0.792	0.876
discrhire	Factor 1	0.701	0.667	0.73	0.64	0.607	0.675
discred							
discrmed	Factor 1	0.672	0.636	0.703	0.614	0.561	0.664
discrpub	Factor 1	0.863	0.842	0.882	0.821	0.777	0.858
discrfam	Factor 1	0.62	0.579	0.66	0.6	0.548	0.654
workspeed	Factor 2	0.851	0.828	0.871	0.909	0.883	0.934
workrep	Factor 2	0.787	0.763	0.81	0.875	0.842	0.905
workemot	Factor 2	0.79	0.764	0.814	0.875	0.841	0.906
workphys	Factor 2	0.754	0.729	0.777	0.842	0.81	0.873
timework	Factor 2	0.526	0.489	0.562	0.487	0.437	0.54
carephys	Factor 3	0.947	0.936	0.957	0.937	0.91	0.961
careemot	Factor 3	0.97	0.959	0.98	0.944	0.918	0.967
carefut	Factor 3	0.894	0.876	0.912	0.895	0.855	0.929
timecare	Factor 3	0.625	0.587	0.667	0.471	0.383	0.575
riskgen	Factor 4	0.849	0.811	0.886	0.758	0.678	0.835
riskfinan	Factor 4	0.673	0.637	0.708	0.608	0.548	0.663
riskrecrea	Factor 4	0.613	0.577	0.648	0.559	0.5	0.619
socsuplove	Factor 5	0.963	0.915	1,013	0.946	0.877	1,015
socsupchores	Factor 5	0.588	0.562	0.611	0.584	0.545	0.617
commfriend	Factor 6	0.533	0.481	0.584	0.535	0.471	0.604
exprfriend	Factor 6	0.765	0.709	0.818	0.768	0.688	0.846
exprother	Factor 6	0.555	0.509	0.602	0.587	0.522	0.644
indepprob	Factor 7	0.866	0.776	0.969	0.731	0.62	0.861
indepgen	Factor 7	0.691	0.606	0.772	0.673	0.58	0.763

Note: The 95% confidence intervals were based on 2000 boostrap samples

Table S18. Factor loadings for final CFA in samples 2 and 3 (Metric invariance, 25 items).

**Item**		Sample 2	CI Lower	CI Upper	Sample 3	CI Lower	CI Upper
discrgen	Factor 1	0.847	0.82	0.87	0.819	0.742	0.875
discrhire	Factor 1	0.705	0.671	0.738	0.707	0.667	0.746
discred	Factor 1	0.732	0.699	0.763	0.696	0.65	0.74
discrmed	Factor 1	0.691	0.655	0.724	0.615	0.54	0.679
discrpub	Factor 1	0.867	0.847	0.883	0.796	0.719	0.856
discrfam	Factor 1	0.621	0.579	0.661	0.591	0.521	0.658
workspeed	Factor 2	0.85	0.826	0.871	0.896	0.869	0.925
workrep	Factor 2	0.783	0.757	0.807	0.847	0.809	0.882
workemot	Factor 2	0.794	0.769	0.818	0.862	0.823	0.899
workphys	Factor 2	0.758	0.733	0.781	0.826	0.79	0.861
timework	Factor 2	0.509	0.469	0.546	0.464	0.413	0.52
carephys	Factor 3	0.947	0.936	0.957	0.946	0.916	0.972
careemot	Factor 3	0.971	0.96	0.98	0.939	0.911	0.965
carefut	Factor 3	0.894	0.875	0.911	0.885	0.839	0.927
timecare	Factor 3	0.627	0.587	0.668	0.49	0.4	0.604
riskgen	Factor 4	0.848	0.809	0.886	0.736	0.642	0.829
riskfinan	Factor 4	0.675	0.637	0.711	0.594	0.52	0.659
riskrecrea	Factor 4	0.613	0.573	0.649	0.534	0.464	0.6
socsuplove	Factor 5	0.967	0.923	1,017	0.964	0.889	1,039
socsupchores	Factor 5	0.586	0.558	0.609	0.584	0.537	0.62
commfriend	Factor 6	0.525	0.471	0.58	0.508	0.438	0.578
exprfriend	Factor 6	0.764	0.71	0.819	0.751	0.656	0.84
exprother	Factor 6	0.562	0.515	0.607	0.563	0.493	0.635
indepprob	Factor 7	0.837	0.749	0.932	0.705	0.603	0.812
indepgen	Factor 7	0.717	0.643	0.795	0.697	0.598	0.794

Note: The 95% confidence intervals were based on 2000 boostrap samples

Table S19. Factor loadings for final CFA samples 2 and 3 (Scalar invariance, 25 items).

**Item**		Sample 2	CI Lower	CI Upper	Sample 3	CI Lower	CI Upper
discrgen	Factor 1	0.846	0.819	0.869	0.815	0.731	0.876
discrhire	Factor 1	0.707	0.674	0.74	0.674	0.634	0.717
discred	Factor 1	0.732	0.7	0.764	0.683	0.638	0.73
discrmed	Factor 1	0.691	0.655	0.724	0.618	0.543	0.681
discrpub	Factor 1	0.866	0.847	0.883	0.797	0.715	0.859
discrfam	Factor 1	0.621	0.579	0.662	0.595	0.527	0.661
workspeed	Factor 2	0.85	0.827	0.871	0.899	0.87	0.929
workrep	Factor 2	0.785	0.759	0.808	0.841	0.804	0.876
workemot	Factor 2	0.792	0.767	0.816	0.862	0.821	0.9
workphys	Factor 2	0.758	0.733	0.781	0.83	0.793	0.866
timework	Factor 2	0.508	0.468	0.545	0.468	0.416	0.523
carephys	Factor 3	0.947	0.936	0.957	0.945	0.915	0.972
careemot	Factor 3	0.971	0.96	0.98	0.939	0.911	0.966
carefut	Factor 3	0.894	0.875	0.911	0.885	0.839	0.927
timecare	Factor 3	0.627	0.587	0.668	0.49	0.4	0.605
riskgen	Factor 4	0.849	0.81	0.888	0.73	0.632	0.825
riskfinan	Factor 4	0.673	0.635	0.709	0.594	0.52	0.659
riskrecrea	Factor 4	0.613	0.574	0.65	0.539	0.469	0.604
socsuplove	Factor 5	0.966	0.921	1.016	0.964	0.891	1.038
socsupchores	Factor 5	0.586	0.559	0.61	0.588	0.543	0.626
commfriend	Factor 6	0.527	0.474	0.581	0.525	0.454	0.596
exprfriend	Factor 6	0.765	0.711	0.819	0.76	0.669	0.845
exprother	Factor 6	0.56	0.513	0.605	0.579	0.51	0.647
indepprob	Factor 7	0.848	0.762	0.946	0.705	0.597	0.819
indepgen	Factor 7	0.707	0.631	0.787	0.685	0.585	0.784

Note: The 95% confidence intervals were based on 2000 boostrap samples

Table S20. Correlations between the factors in samples 1, 2 and 3.

**Sample 1**	Independent	Emotional Intelligence	Social Support	Risk-taking	Caregiver strain	Work strain	Discrimination
Independent	1,000						
Emotional Intelligence	-0.101	1.000					
Social Support	-0.105	0.364	1.000				
Risk-taking	0.256	0.236	0.145	.,000			
Caregiver strain	0.071	0.133	0.101	0.043	1.000		
Work strain	0.151	0.129	0.032	0.219	0.105	1.000	
Discrimination	0.031	0.207	0.021	0.049	0.13	0.094	1.000

**Sample 2**	Independent	Emotional Intelligence	Social Support	Risk-taking	Caregiver strain	Work strain	Discrimination
Independent	1.000						
Emotional Intelligence	-0.196	1.000					
Social Support	-0.077	0.314	1.000				
Risk-taking	0.174	0.18	0.149	1.000			
Caregiver strain	0.058	0.055	0.036	0.032	1.000		
Work strain	0.105	0.076	0.036	0.133	0.101	1.000	
Discrimination	-0.087	0.182	-0.053	0.001	0.132	0.108	1.000

**Sample 3**	Independent	Emotional Intelligence	Social Support	Risk-taking	Caregiver strain	Work strain	Discrimination
Independent	1.000						
Emotional Intelligence	-.261	1.000					
Social Support	-.224	.340	1.000				
Risk-taking	.104	.194	.009	1.000			
Caregiver strain	-.076	.055	.041	-.048	1.000		
Work strain	.089	.065	-.010	-.027	.108	1.000	
Discrimination	.012	.118	.033	-.018	.220	.068	1.000

Note: Sample 1= 2009, Sample 2=2054, Sample 3= 449. The discrimination variable in sample 3 was computed without the discred variable.

Table S21. Negative binomial regression predicting number of days with poor physical health (during past 30 days) (with gender identity as covariate).

	SAMPLE 1			*SAMPLE 2*		
	Incidence rate ratio	95% CI		Incidence rate ratio	95% CI	
Caregiver strain	1.20	1.12	1.29	1.11	1.04	1.18
Work strain	0.96	0.88	1.05	1.01	0.93	1.10
Independence	0.93	0.85	1.01	0.85	0.75	0.95
Risk-taking	0.95	0.87	1.04	1.00	0.92	1.08
Emotional intelligence	0.93	0.85	1.03	0.92	0.85	1.01
Social support	0.95	0.89	1.03	0.94	0.89	1.00
Discrimination	1.45	1.33	1.58	1.43	1.31	1.57
Year of birth	0.99	0.98	1.00	0.99	0.98	0.99
Personal Income ($10,000-$19,999)	1.02	0.80	1.29	1.07	0.86	1.33
Personal Income ($20,000-$29,999)	0.94	0.73	1.21	1.01	0.81	1.25
Personal Income ($30,000-$39,999)	0.84	0.64	1.10	0.87	0.68	1.11
Personal Income ($40,000-$49,999)	1.01	0.76	1.33	0.71	0.56	0.91
Personal Income ($50,000-$59,999)	0.93	0.68	1.27	0.68	0.50	0.92
Personal Income ($60,000-$69,999)	0.83	0.60	1.16	0.70	0.52	0.95
Personal Income ($70,000-$79,999)	0.82	0.54	1.23	0.61	0.44	0.85
Personal Income ($80,000-$89,999)	0.65	0.41	1.02	0.45	0.32	0.62
Personal Income ($90,000-$99,999)	1.03	0.56	1.92	0.80	0.54	1.19
Personal Income ($100,000-$149,999)	0.78	0.52	1.17	0.67	0.47	0.96
Personal Income (More than $150,000)	1.36	0.88	2.10	0.55	0.29	1.03
Educational Level (High school graduate)	1.04	0.64	1.70	0.88	0.50	1.55
Educational Level (Some college credit, no degree)	0.88	0.55	1.40	1.12	0.65	1.92
Educational Level (Trade/Technical/Vocational education)	0.86	0.48	1.54	0.83	0.46	1.51
Educational Level (Associate degree)	0.79	0.48	1.30	1.00	0.58	1.74
Educational Level (Bachelor's degree)	0.75	0.47	1.20	0.85	0.50	1.46
Educational Level (Master's degree)	0.89	0.54	1.48	0.84	0.48	1.48
Educational Level (professional degree)	0.71	0.24	2.05	0.92	0.43	1.95
Educational Level (Doctorate degree)	0.68	0.37	1.25	0.69	0.35	1.33
Ethnicity (White)	1.02	0.77	1.36	0.96	0.74	1.24
Ethnicity (Hispanic, Latinx, Spanish)	0.98	0.71	1.37	0.96	0.70	1.31
Ethnicity (Black, African American)	0.94	0.67	1.30	0.86	0.66	1.12
Ethnicity (Asian)	0.83	0.60	1.15	0.87	0.61	1.24
Ethnicity (Native)	0.91	0.67	1.24	1.64	1.12	2.41
Woman	0.95	0.82	1.11	0.93	0.80	1.07
Non-binary/Gender fluid + Gender=other	0.96	0.58	1.60	0.44	0.17	1.15
**Goodness of Fit**						
Deviance/df	1.114			1.120		
Pearson Chi-Square/df	1.240			1.159		
Loglikelihood	-4848.379			-5161.248		
AIC	9768.758			10394.496		
**Omnibus tests**						
	183.600			204.826		
Chi-square						
df	34			34		
N	1881			2008		

Note: Participants reporting Native Hawaiian/Pacific Islander, Middle east/North Africa or Other as ethnicity are the reference group for the ethnicity variables. Personal Income < $10.000 is the reference group for the "Personal Income" variables. Educational level < high school diploma is the reference group for the "Educational Level" variables. The models are computed with robust covariance matrix estimation.

Table S22. Negative Binomial regression predicting number of days with poor mental health (during past 30 days) (with gender identity as covariate).

	SAMPLE 1			*SAMPLE 2*		
	Incidence rate ratio	95% CI		Incidence rate ratio	95% CI	
Caregiver strain	1.21	1.15	1.27	1.20	1.14	1.27
Work strain	1.03	0.96	1.11	1.12	1.04	1.21
Independence	0.98	0.91	1.04	0.83	0.74	0.93
Risk-taking	0.86	0.80	0.93	0.99	0.91	1.08
Emotional intelligence	0.97	0.90	1.05	0.86	0.79	0.94
Social support	0.86	0.81	0.90	0.81	0.76	0.86
Discrimination	1.42	1.33	1.51	1.36	1.24	1.49
Year of birth	1.02	1.01	1.02	1.02	1.01	1.02
Personal Income ($10,000-$19,999)	0.98	0.82	1.16	1.03	0.84	1.25
Personal Income ($20,000-$29,999)	0.75	0.62	0.91	0.89	0.71	1.10
Personal Income ($30,000-$39,999)	0.78	0.63	0.96	0.85	0.68	1.05
Personal Income ($40,000-$49,999)	0.76	0.62	0.95	0.63	0.50	0.80
Personal Income ($50,000-$59,999)	0.81	0.65	1.01	0.51	0.38	0.68
Personal Income ($60,000-$69,999)	0.72	0.56	0.92	0.54	0.40	0.73
Personal Income ($70,000-$79,999)	0.69	0.50	0.96	0.55	0.39	0.76
Personal Income ($80,000-$89,999)	0.75	0.52	1.08	0.77	0.47	1.26
Personal Income ($90,000-$99,999)	0.98	0.66	1.45	0.61	0.38	0.97
Personal Income ($100,000-$149,999)	0.59	0.42	0.82	0.62	0.44	0.88
Personal Income (More than $150,000)	1.00	0.67	1.48	0.43	0.25	0.74
Educational Level (High school graduate)	1.06	0.72	1.58	1.50	0.86	2.61
Educational Level (Some college credit, no degree)	1.04	0.72	1.52	1.68	0.98	2.85
Educational Level (Trade/Technical/Vocational education)	1.15	0.73	1.81	1.90	1.03	3.53
Educational Level (Associate degree)	1.01	0.67	1.52	1.61	0.93	2.77
Educational Level (Bachelor's degree)	0.88	0.61	1.28	1.53	0.90	2.60
Educational Level (Master's degree)	1.07	0.72	1.59	1.61	0.92	2.81
Educational Level (professional degree)	0.71	0.36	1.41	1.84	0.90	3.77
Educational Level (Doctorate degree)	0.91	0.57	1.46	1.74	0.87	3.48
Ethnicity (White)	1.53	1.24	1.89	1.32	1.00	1.73
Ethnicity (Hispanic, Latinx, Spanish)	1.06	0.85	1.32	1.10	0.83	1.47
Ethnicity (Black, African American)	1.27	1.00	1.62	0.94	0.71	1.24
Ethnicity (Asian)	1.13	0.88	1.45	1.08	0.75	1.54
Ethnicity (Native)	1.06	0.80	1.41	1.88	1.27	2.79
Woman	0.95	0.84	1.07	1.20	1.04	1.39
Non-binary/Gender fluid + Gender=other	1.24	0.96	1.61	0.92	0.43	1.98
**Goodness of Fit**						
Deviance/df	1.173			1.131		
Pearson Chi-Square/df	1.016			1.106		
Loglikelihood	-5669.909			-5571.861		
AIC	11411.818			11215.721		
**Omnibus tests**						
	285.752			316.349		
Chi-square						
df	34			34		
N	1883			2005		

Note: Participants reporting Native Hawaiian/Pacific Islander, Middle east/North Africa or Other as ethnicity are the reference group for the ethnicity variables. Personal Income < $10.000 is the reference group for the "Personal Income" variables. Educational level < high school diploma is the reference group for the "Educational Level" variables. The models are computed with robust covariance matrix estimation.

Table S23. Negative binomial regression predicting number of days where poor mental or physical health prevented the respondent from doing usual activities (during past 30 days) (with gender identity as covariate).

	SAMPLE 1			*SAMPLE 2*		
	Incidence rate ratio	95% CI		Incidence rate ratio	95% CI	
Caregiver strain	1.13	1.05	1.22	1.10	1.02	1.19
Work strain	0.88	0.80	0.97	0.98	0.90	1.08
Independence	1.02	0.94	1.12	0.88	0.77	1.01
Risk-taking	0.93	0.85	1.02	1.02	0.93	1.11
Emotional intelligence	0.99	0.89	1.10	0.92	0.83	1.01
Social support	0.92	0.86	0.99	0.87	0.81	0.94
Discrimination	1.74	1.59	1.90	1.57	1.41	1.74
Year of birth	1.01	1.00	1.01	1.00	1.00	1.01
Personal Income ($10,000-$19,999)	1.01	0.80	1.27	0.90	0.70	1.16
Personal Income ($20,000-$29,999)	0.90	0.70	1.16	0.92	0.72	1.17
Personal Income ($30,000-$39,999)	0.76	0.56	1.02	0.77	0.59	1.01
Personal Income ($40,000-$49,999)	0.92	0.68	1.23	0.65	0.48	0.87
Personal Income ($50,000-$59,999)	0.76	0.56	1.04	0.52	0.37	0.73
Personal Income ($60,000-$69,999)	0.63	0.44	0.90	0.68	0.47	0.97
Personal Income ($70,000-$79,999)	0.67	0.42	1.05	0.62	0.43	0.89
Personal Income ($80,000-$89,999)	0.99	0.57	1.70	0.64	0.40	1.03
Personal Income ($90,000-$99,999)	1.00	0.51	1.95	0.70	0.44	1.11
Personal Income ($100,000-$149,999)	0.91	0.58	1.42	0.70	0.46	1.06
Personal Income (More than $150,000)	1.57	0.95	2.60	0.75	0.38	1.47
Educational Level (High school graduate)	1.07	0.64	1.78	0.99	0.51	1.92
Educational Level (Some college credit, no degree)	0.85	0.52	1.39	1.14	0.61	2.13
Educational Level (Trade/Technical/Vocational education)	1.32	0.74	2.36	0.81	0.40	1.65
Educational Level (Associate degree)	0.82	0.48	1.40	0.96	0.51	1.82
Educational Level (Bachelor’s degree)	0.87	0.53	1.41	0.77	0.42	1.44
Educational Level (Master’s degree)	0.72	0.43	1.22	0.70	0.37	1.33
Educational Level (professional degree)	0.24	0.10	0.56	0.51	0.22	1.18
Educational Level (Doctorate degree)	0.64	0.36	1.15	0.61	0.28	1.31
Ethnicity (White)	1.11	0.84	1.48	0.89	0.65	1.21
Ethnicity (Hispanic, Latinx, Spanish)	0.88	0.66	1.18	1.00	0.70	1.43
Ethnicity (Black, African American)	0.99	0.72	1.37	0.76	0.54	1.06
Ethnicity (Asian)	0.77	0.56	1.05	0.85	0.56	1.31
Ethnicity (Native)	0.93	0.62	1.39	2.03	1.26	3.28
Woman	0.88	0.74	1.03	1.09	0.92	1.29
Non-binary/Gender fluid + Gender=other	1.30	0.82	2.04	0.62	0.16	2.45
**Goodness of Fit**						
Deviance/df	1.081			1.057		
Pearson Chi-Square/df	1.165			1.148		
Loglikelihood	-4584.605			-4640.077		
AIC	9241.210			9352.154		
**Omnibus tests**						
Chi-square	250.517			222.470		
df	34			34		
N	1879			2007		

Note: Participants reporting Native Hawaiian/Pacific Islander, Middle east/North Africa or Other as ethnicity are the reference group for the ethnicity variables. Personal Income < $10.000 is the reference group for the “Personal Income” variables. Educational level < high school diploma is the reference group for the “Educational Level” variables. The models are computed with robust covariance matrix estimation.

Table S24. Logistic regression predicting general health status (excellent, very good, good= 0, fair, poor= 1) (with gender identity as covariate)

	SAMPLE 1			*SAMPLE 2*		
	Incidence rate ratio	95% CI		Incidence rate ratio	95% CI	
Caregiver strain	1.28	1.12	1.47	1.17	1.03	1.34
Work strain	0.87	0.73	1.05	0.99	0.83	1.17
Independence	0.91	0.77	1.06	0.71	0.56	0.90
Risk-taking	0.56	0.47	0.67	0.69	0.58	0.81
Emotional intelligence	0.92	0.76	1.11	0.89	0.75	1.06
Social support	0.83	0.73	0.95	0.78	0.69	0.88
Discrimination	1.43	1.18	1.73	1.57	1.29	1.89
Year of birth	0.98	0.97	0.99	0.98	0.97	0.99
Personal Income ($10,000-$19,999)	0.79	0.52	1.20	1.19	0.78	1.80
Personal Income ($20,000-$29,999)	0.88	0.57	1.36	0.83	0.54	1.28
Personal Income ($30,000-$39,999)	0.61	0.37	1.02	0.82	0.51	1.32
Personal Income ($40,000-$49,999)	0.76	0.46	1.28	0.62	0.37	1.04
Personal Income ($50,000-$59,999)	0.62	0.37	1.05	0.52	0.29	0.93
Personal Income ($60,000-$69,999)	0.86	0.48	1.55	0.78	0.41	1.49
Personal Income ($70,000-$79,999)	0.61	0.28	1.32	0.63	0.33	1.22
Personal Income ($80,000-$89,999)	0.19	0.04	0.81	0.52	0.20	1.33
Personal Income ($90,000-$99,999)	0.30	0.06	1.51	1.01	0.46	2.23
Personal Income ($100,000-$149,999)	0.11	0.02	0.48	0.60	0.30	1.20
Personal Income (More than $150,000)	0.09	0.01	0.71	0.73	0.23	2.33
Educational Level (High school graduate)	0.45	0.19	1.09	0.84	0.28	2.54
Educational Level (Some college credit, no degree)	0.58	0.25	1.32	0.93	0.32	2.68
Educational Level (Trade/Technical/Vocational education)	0.56	0.19	1.60	0.79	0.25	2.51
Educational Level (Associate degree)	0.42	0.17	1.05	1.01	0.34	2.96
Educational Level (Bachelor's degree)	0.41	0.18	0.95	0.50	0.17	1.45
Educational Level (Master's degree)	0.47	0.19	1.15	0.39	0.13	1.19
Educational Level (professional degree)	0.35	0.06	2.04	0.24	0.05	1.24
Educational Level (Doctorate degree)	0.17	0.04	0.74	0.63	0.17	2.35
Ethnicity (White)	1.95	1.08	3.53	0.99	0.52	1.87
Ethnicity (Hispanic, Latinx, Spanish)	0.83	0.42	1.63	1.06	0.55	2.04
Ethnicity (Black, African American)	2.31	1.20	4.42	0.90	0.46	1.77
Ethnicity (Asian)	1.69	0.88	3.27	0.76	0.32	1.82
Ethnicity (Native)	0.46	0.18	1.16	0.86	0.38	1.97
Woman	0.57	0.42	0.78	0.65	0.48	0.87
Non-binary/Gender fluid + Gender=other	1.08	0.40	2.93	0.33	0.03	3.69
**Goodness of Fit**						
Deviance/df	0.826			0.877		
Pearson Chi-Square/df	1.015			1.015		
Loglikelihood	-766.888			-867.561		
AIC	1603.776			1805.123		
**Omnibus tests**						
Chi-square	210.935			191.691		
df	34			34		
N	1891			2013		

Note: Participants reporting Native Hawaiian/Pacific Islander, Middle east/North Africa or Other as ethnicity are the reference group for the ethnicity variables. Personal Income < $10.000 is the reference group for the "Personal Income" variables. Educational level < high school diploma is the reference group for the "Educational Level" variables. The models are computed with robust covariance matrix estimation.

Table S25. Logistic regression predicting vaping (not vaping=0, vaping=1) (with gender identity as covariate).

	SAMPLE 1			*SAMPLE 2*		
	Incidence rate ratio	95% CI		Incidence rate ratio	95% CI	
Caregiver strain	1.06	0.90	1.25	1.10	0.93	1.29
Work strain	1.21	0.96	1.53	1.29	1.05	1.59
Independence	1.18	0.96	1.45	1.05	0.77	1.44
Risk-taking	1.18	0.97	1.45	1.00	0.80	1.24
Emotional intelligence	1.39	1.12	1.72	1.05	0.84	1.31
Social support	0.98	0.84	1.14	0.89	0.76	1.04
Discrimination	1.40	1.16	1.70	1.47	1.18	1.83
Year of birth	0.98	0.97	1.00	1.01	1.00	1.03
Personal Income ($10,000-$19,999)	1.89	0.95	3.76	1.20	0.60	2.38
Personal Income ($20,000-$29,999)	1.63	0.83	3.21	1.65	0.87	3.11
Personal Income ($30,000-$39,999)	2.56	1.34	4.90	1.03	0.52	2.06
Personal Income ($40,000-$49,999)	2.04	0.99	4.20	1.08	0.52	2.21
Personal Income ($50,000-$59,999)	2.41	1.21	4.80	1.47	0.71	3.03
Personal Income ($60,000-$69,999)	2.87	1.38	5.98	1.30	0.57	2.98
Personal Income ($70,000-$79,999)	1.65	0.72	3.78	0.89	0.34	2.32
Personal Income ($80,000-$89,999)	0.69	0.16	3.06	1.02	0.31	3.30
Personal Income ($90,000-$99,999)	4.67	1.84	11.85	0.99	0.32	3.05
Personal Income ($100,000-$149,999)	2.19	0.96	4.98	0.90	0.35	2.30
Personal Income (More than $150,000)	7.25	3.29	16.00	0.45	0.05	3.75
Educational Level (High school graduate)	0.36	0.13	0.94	0.85	0.16	4.60
Educational Level (Some college credit, no degree)	0.30	0.12	0.78	0.81	0.16	4.19
Educational Level (Trade/Technical/Vocational education)	0.38	0.11	1.33	0.45	0.07	2.89
Educational Level (Associate degree)	0.28	0.10	0.79	1.18	0.23	6.08
Educational Level (Bachelor's degree)	0.18	0.07	0.46	0.66	0.13	3.38
Educational Level (Master's degree)	0.26	0.10	0.69	0.45	0.08	2.46
Educational Level (professional degree)	0.30	0.06	1.42	0.50	0.06	4.48
Educational Level (Doctorate degree)	0.21	0.06	0.71	0.49	0.06	4.41
Ethnicity (White)	1.69	0.93	3.05	1.04	0.53	2.05
Ethnicity (Hispanic, Latinx, Spanish)	1.18	0.60	2.30	1.35	0.62	2.92
Ethnicity (Black, African American)	1.47	0.70	3.12	0.73	0.35	1.53
Ethnicity (Asian)	1.08	0.53	2.22	0.73	0.28	1.86
Ethnicity (Native)	1.48	0.60	3.66	1.66	0.69	4.01
Woman	0.42	0.30	0.60	0.74	0.50	1.09
Non-binary/Gender fluid + Gender=other	0.44	0.09	2.23	0.64	0.08	5.30
**Goodness of Fit**						
Deviance/df	0.822			0.571		
Pearson Chi-Square/df	1.034			1.014		
Loglikelihood	-761.661			-564.438		
AIC	1593.322			1198.877		
**Omnibus tests**						
	269.616			60.175		
Chi-square						
df	34			34		
				2012		
N	1888					

Note: Participants reporting Native Hawaiian/Pacific Islander, Middle east/North Africa or Other as ethnicity are the reference group for the ethnicity variables. Personal Income < $10.000 is the reference group for the "Personal Income" variables. Educational level < high school diploma is the reference group for the "Educational Level" variables. The models are computed with robust covariance matrix estimation.

Table S26. Logistic regression predicting smoking (not smoking=0, smoking=1) (with gender identity as covariate).

	SAMPLE 1			*SAMPLE 2*		
	Incidence rate ratio	95% CI		Incidence rate ratio	95% CI	
Caregiver strain	1.41	1.23	1.61	1.18	1.05	1.33
Work strain	1.36	1.14	1.63	1.25	1.08	1.46
Independence	1.22	1.03	1.46	1.07	0.85	1.35
Risk-taking	1.43	1.20	1.71	1.06	0.92	1.23
Emotional intelligence	1.46	1.22	1.75	1.12	0.96	1.30
Social support	0.98	0.86	1.11	0.98	0.87	1.09
Discrimination	1.11	0.92	1.33	1.17	0.98	1.39
Year of birth	0.97	0.96	0.98	1.00	0.99	1.01
Personal Income ($10,000-$19,999)	1.06	0.64	1.74	1.24	0.81	1.89
Personal Income ($20,000-$29,999)	1.41	0.87	2.28	0.89	0.58	1.38
Personal Income ($30,000-$39,999)	0.83	0.47	1.44	1.18	0.75	1.85
Personal Income ($40,000-$49,999)	1.21	0.70	2.10	0.94	0.59	1.51
Personal Income ($50,000-$59,999)	1.13	0.64	1.99	0.88	0.53	1.46
Personal Income ($60,000-$69,999)	1.32	0.73	2.38	0.86	0.47	1.57
Personal Income ($70,000-$79,999)	1.18	0.60	2.34	1.04	0.58	1.86
Personal Income ($80,000-$89,999)	0.52	0.16	1.65	1.47	0.73	2.95
Personal Income ($90,000-$99,999)	1.96	0.85	4.54	0.62	0.28	1.39
Personal Income ($100,000-$149,999)	0.92	0.48	1.77	0.95	0.52	1.74
Personal Income (More than $150,000)	4.58	2.24	9.40	0.38	0.12	1.20
Educational Level (High school graduate)	1.33	0.43	4.10	0.70	0.25	1.94
Educational Level (Some college credit, no degree)	1.04	0.35	3.13	0.65	0.24	1.75
Educational Level (Trade/Technical/Vocational education)	1.73	0.50	6.00	1.00	0.35	2.90
Educational Level (Associate degree)	0.72	0.23	2.27	0.70	0.26	1.91
Educational Level (Bachelor's degree)	0.49	0.16	1.48	0.30	0.11	0.80
Educational Level (Master's degree)	0.45	0.14	1.41	0.23	0.08	0.66
Educational Level (professional degree)	0.44	0.08	2.29	0.32	0.08	1.26
Educational Level (Doctorate degree)	0.77	0.22	2.68	0.18	0.04	0.79
Ethnicity (White)	1.40	0.70	2.79	0.69	0.39	1.23
Ethnicity (Hispanic, Latinx, Spanish)	0.94	0.51	1.75	0.44	0.23	0.85
Ethnicity (Black, African American)	0.84	0.37	1.91	0.41	0.22	0.78
Ethnicity (Asian)	0.44	0.18	1.07	0.29	0.12	0.68
Ethnicity (Native)	0.75	0.29	1.94	1.66	0.85	3.22
Woman	0.65	0.48	0.88	0.91	0.70	1.18
Non-binary/Gender fluid + Gender=other	0.18	0.02	1.38	0.28	0.04	1.87
**Goodness of Fit**						
Deviance/df	0.822			1.007		
Pearson Chi-Square/df	1.034			1.007		
Loglikelihood	-761.661			-994.960		
AIC	1593.322			2059.921		
**Omnibus tests**						
	269.616			146.339		
Chi-square						
df	34			34		
N	1889			2012		

Note: Participants reporting Native Hawaiian/Pacific Islander, Middle east/North Africa or Other as ethnicity are the reference group for the ethnicity variables. Personal Income < $10.000 is the reference group for the "Personal Income" variables. Educational level < high school diploma is the reference group for the "Educational Level" variables. The models are computed with robust covariance matrix estimation.

Table S27. Logistic regression predicting binge drinking (less than monthly=0, monthly, weekly, and daily=1) (with gender identity as covariate).

	SAMPLE 1			*SAMPLE 2*		
	Incidence rate ratio	95% CI		Incidence rate ratio	95% CI	
Caregiver strain	0.92	0.80	1.05	0.91	0.80	1.04
Work strain	1.28	1.09	1.52	1.03	0.88	1.21
Independence	1.08	0.93	1.24	1.19	0.94	1.49
Risk-taking	1.53	1.31	1.78	1.25	1.08	1.45
Emotional intelligence	1.18	1.00	1.40	1.04	0.89	1.22
Social support	1.01	0.90	1.13	0.87	0.78	0.97
Discrimination	1.17	0.99	1.37	1.12	0.93	1.34
Year of birth	1.02	1.01	1.03	1.03	1.02	1.04
Personal Income ($10,000-$19,999)	1.03	0.65	1.62	1.27	0.78	2.04
Personal Income ($20,000-$29,999)	1.15	0.73	1.82	1.94	1.23	3.06
Personal Income ($30,000-$39,999)	1.20	0.76	1.90	1.33	0.81	2.17
Personal Income ($40,000-$49,999)	1.31	0.81	2.13	1.79	1.08	2.96
Personal Income ($50,000-$59,999)	0.93	0.56	1.53	1.37	0.80	2.36
Personal Income ($60,000-$69,999)	1.21	0.71	2.06	1.98	1.11	3.55
Personal Income ($70,000-$79,999)	0.83	0.45	1.54	1.44	0.78	2.65
Personal Income ($80,000-$89,999)	1.17	0.53	2.58	1.15	0.52	2.57
Personal Income ($90,000-$99,999)	1.68	0.73	3.86	2.29	1.13	4.62
Personal Income ($100,000-$149,999)	1.16	0.63	2.12	1.93	1.05	3.57
Personal Income (More than $150,000)	2.08	1.04	4.16	2.42	0.99	5.88
Educational Level (High school graduate)	1.20	0.40	3.59	0.50	0.15	1.65
Educational Level (Some college credit, no degree)	1.67	0.58	4.79	0.90	0.29	2.82
Educational Level (Trade/Technical/Vocational education)	2.37	0.71	7.93	0.85	0.25	2.92
Educational Level (Associate degree)	1.36	0.45	4.12	0.65	0.20	2.09
Educational Level (Bachelor's degree)	1.52	0.53	4.36	0.63	0.20	1.97
Educational Level (Master's degree)	1.80	0.62	5.28	0.57	0.18	1.86
Educational Level (professional degree)	3.24	0.77	13.57	0.58	0.14	2.50
Educational Level (Doctorate degree)	1.76	0.52	5.91	0.65	0.15	2.73
Ethnicity (White)	1.89	1.08	3.30	0.92	0.53	1.60
Ethnicity (Hispanic, Latinx, Spanish)	1.64	0.96	2.79	0.83	0.47	1.46
Ethnicity (Black, African American)	0.75	0.36	1.54	1.04	0.56	1.92
Ethnicity (Asian)	0.58	0.30	1.12	0.54	0.27	1.08
Ethnicity (Native)	0.46	0.19	1.09	0.79	0.38	1.64
Woman	0.57	0.44	0.74			
Non-binary/Gender fluid + Gender=other	0.53	0.18	1.56			
Gender Dummy (woman=1)				0.78	0.60	1.03
**Goodness of Fit**						
Deviance/df	1.001			0.571		
Pearson Chi-Square/df	1.017			1.014		
Loglikelihood	-923.706			-564.438		
AIC	1917.411			1198.877		
**Omnibus tests**						
Chi-square	212.582			60.175		
df	34			34		
N	1888			2000		

Note: Participants reporting Native Hawaiian/Pacific Islander, Middle east/North Africa or Other as ethnicity are the reference group for the ethnicity variables. Personal Income < $10.000 is the reference group for the "Personal Income" variables. Educational level < high school diploma is the reference group for the "Educational Level" variables. It was not possible to estimate the effect of "Non-binary/Gender fluid + Gender=other" in Sample 2. Instead, we included a dischotomous gender variable (man=0, woman=1). The models are computed with robust covariance matrix estimation.

Table S28. Logistic regression predicting overweight (BMI<25=0, BMI≥25 =1) (with gender identity as covariate).

	SAMPLE 1			*SAMPLE 2*		
				Incidence rate ratio	95% CI	
Caregiver strain	1.18	1.05	1.31	1.27	1.14	1.41
Work strain	1.04	0.91	1.19	1.16	1.02	1.33
Independence	0.99	0.88	1.12	1.05	0.88	1.27
Risk-taking	0.81	0.72	0.93	0.86	0.76	0.97
Emotional intelligence	0.99	0.87	1.14	1.03	0.90	1.18
Social support	0.99	0.90	1.09	1.07	0.97	1.18
Discrimination	1.22	1.06	1.41	1.27	1.09	1.49
Year of birth	0.98	0.97	0.99	0.97	0.97	0.98
Personal Income ($10,000-$19,999)	1.14	0.80	1.61	0.83	0.57	1.19
Personal Income ($20,000-$29,999)	1.23	0.86	1.77	0.83	0.58	1.20
Personal Income ($30,000-$39,999)	1.16	0.80	1.68	0.77	0.53	1.14
Personal Income ($40,000-$49,999)	1.19	0.81	1.75	0.77	0.51	1.14
Personal Income ($50,000-$59,999)	1.51	1.01	2.26	1.15	0.75	1.77
Personal Income ($60,000-$69,999)	1.33	0.85	2.09	1.27	0.76	2.14
Personal Income ($70,000-$79,999)	1.32	0.78	2.22	2.20	1.30	3.71
Personal Income ($80,000-$89,999)	1.20	0.60	2.38	0.98	0.54	1.78
Personal Income ($90,000-$99,999)	1.98	0.88	4.48	1.05	0.58	1.90
Personal Income ($100,000-$149,999)	1.06	0.63	1.80	1.00	0.59	1.69
Personal Income (More than $150,000)	1.26	0.65	2.45	0.96	0.43	2.15
Educational Level (High school graduate)	0.86	0.39	1.88	0.24	0.07	0.85
Educational Level (Some college credit, no degree)	0.89	0.41	1.90	0.32	0.09	1.10
Educational Level (Trade/Technical/Vocational education)	0.87	0.34	2.25	0.21	0.06	0.76
Educational Level (Associate degree)	1.03	0.46	2.34	0.23	0.07	0.81
Educational Level (Bachelor's degree)	0.58	0.27	1.24	0.21	0.06	0.73
Educational Level (Master's degree)	0.76	0.34	1.69	0.21	0.06	0.74
Educational Level (professional degree)	0.58	0.18	1.80	0.13	0.03	0.52
Educational Level (Doctorate degree)	0.68	0.26	1.77	0.13	0.03	0.51
Ethnicity (White)	1.11	0.72	1.71	1.02	0.63	1.66
Ethnicity (Hispanic, Latinx, Spanish)	1.31	0.83	2.08	1.57	0.95	2.59
Ethnicity (Black, African American)	1.68	1.01	2.79	1.14	0.66	1.96
Ethnicity (Asian)	0.51	0.31	0.83	0.52	0.29	0.93
Ethnicity (Native)	2.11	1.02	4.36	1.20	0.62	2.30
Woman	0.77	0.62	0.97	0.58	0.47	0.73
Non-binary/Gender fluid + Gender=other	0.95	0.44	2.08	0.35	0.06	2.17
**Goodness of Fit**						
Deviance/df	1.315			1.283		
Pearson Chi-Square/df	1.020			1.019		
Loglikelihood	-1202.188			-1257.609		
AIC	2474.377			2585.218		
**Omnibus tests**						
	138.059			165.330		
Chi-square						
df	34			34		
	1864					
N				1996		

Note: Participants reporting Native Hawaiian/Pacific Islander, Middle east/North Africa or Other as ethnicity are the reference group for the ethnicity variables. Personal Income < $10.000 is the reference group for the "Personal Income" variables. Educational level < high school diploma is the reference group for the "Educational Level" variables. The models are computed with robust covariance matrix estimation.

Table S29. Negative binomial regression predicting number of days with poor physical health (during past 30 days) (with sex as covariate).

	SAMPLE 1			*SAMPLE 2*		
	Incidence rate ratio	95% CI		Incidence rate ratio	95% CI	
Caregiver strain	1.20	1.12	1.29	1.11	1.04	1.18
Work strain	0.96	0.87	1.05	1.02	0.94	1.11
Independence	0.93	0.85	1.01	0.84	0.75	0.95
Risk-taking	0.95	0.87	1.04	1.00	0.92	1.08
Emotional intelligence	0.93	0.84	1.03	0.93	0.85	1.01
Social support	0.95	0.89	1.02	0.94	0.89	1.00
Discrimination	1.46	1.34	1.59	1.43	1.30	1.57
Year of birth	0.99	0.98	1.00	0.99	0.98	0.99
Personal Income ($10,000-$19,999)	1.01	0.79	1.29	1.07	0.86	1.34
Personal Income ($20,000-$29,999)	0.94	0.73	1.21	1.01	0.82	1.25
Personal Income ($30,000-$39,999)	0.83	0.64	1.09	0.87	0.68	1.12
Personal Income ($40,000-$49,999)	1.00	0.75	1.33	0.71	0.56	0.91
Personal Income ($50,000-$59,999)	0.92	0.67	1.26	0.68	0.50	0.93
Personal Income ($60,000-$69,999)	0.83	0.60	1.15	0.70	0.52	0.95
Personal Income ($70,000-$79,999)	0.81	0.54	1.22	0.62	0.44	0.86
Personal Income ($80,000-$89,999)	0.66	0.42	1.03	0.45	0.32	0.63
Personal Income ($90,000-$99,999)	1.03	0.55	1.91	0.76	0.51	1.15
Personal Income ($100,000-$149,999)	0.77	0.51	1.16	0.68	0.48	0.96
Personal Income (More than $150,000)	1.26	0.81	1.95	0.55	0.29	1.03
Educational Level (High school graduate)	1.04	0.64	1.70	0.88	0.50	1.55
Educational Level (Some college credit, no degree)	0.88	0.55	1.40	1.11	0.65	1.90
Educational Level (Trade/Technical/Vocational education)	0.87	0.49	1.55	0.84	0.46	1.51
Educational Level (Associate degree)	0.80	0.48	1.32	1.00	0.58	1.74
Educational Level (Bachelor's degree)	0.76	0.47	1.20	0.85	0.50	1.46
Educational Level (Master's degree)	0.90	0.54	1.48	0.85	0.48	1.48
Educational Level (professional degree)	0.72	0.25	2.10	0.92	0.44	1.96
Educational Level (Doctorate degree)	0.69	0.38	1.26	0.69	0.36	1.33
Ethnicity (White)	1.07	0.80	1.43	0.96	0.74	1.25
Ethnicity (Hispanic, Latinx, Spanish)	1.01	0.72	1.40	0.97	0.71	1.32
Ethnicity (Black, African American)	0.97	0.70	1.35	0.87	0.66	1.14
Ethnicity (Asian)	0.86	0.62	1.19	0.88	0.62	1.26
Ethnicity (Native)	0.91	0.67	1.23	1.62	1.11	2.38
Sex (male=0, female=1)	0.94	0.81	1.10	0.93	0.80	1.08
**Goodness of Fit**						
Deviance/df	1.114			1.120		
Pearson Chi-Square/df	1.240			1.159		
Loglikelihood	-4827.875			-5152.368		
AIC	9725.751			10374.737		
**Omnibus tests**						
Chi-square	179.945			200.473		
df	33			33		
N	1874			2005		

Note: Participants reporting Native Hawaiian/Pacific Islander, Middle east/North Africa or Other as ethnicity are the reference group for the ethnicity variables. Personal Income < $10.000 is the reference group for the "Personal Income" variables. Educational level < high school diploma is the reference group for the "Educational Level" variables. The models are computed with robust covariance matrix estimation.

Table S30. Negative Binomial regression predicting number of days with poor mental health (during past 30 days) (with sex as covariate).

	SAMPLE 1				*SAMPLE 2*			
	Incidence ratio	rate	95% CI		Incidence ratio	rate	95% CI	
Caregiver strain		1.21	1.14	1.27		1.20	1.14	1.27
Work strain		1.03	0.96	1.11		1.12	1.04	1.21
Independence		0.97	0.91	1.04		0.83	0.74	0.94
Risk-taking		0.86	0.80	0.92		0.99	0.91	1.08
Emotional intelligence		0.97	0.89	1.05		0.86	0.78	0.93
Social support		0.86	0.81	0.90		0.81	0.76	0.86
Discrimination		1.44	1.34	1.53		1.35	1.24	1.48
Year of birth		1.02	1.01	1.02		1.02	1.01	1.02
Personal Income ($10,000-$19,999)		0.98	0.82	1.17		1.03	0.84	1.26
Personal Income ($20,000-$29,999)		0.74	0.61	0.90		0.89	0.72	1.10
Personal Income ($30,000-$39,999)		0.78	0.63	0.95		0.85	0.68	1.05
Personal Income ($40,000-$49,999)		0.76	0.61	0.95		0.64	0.50	0.80
Personal Income ($50,000-$59,999)		0.81	0.65	1.01		0.51	0.38	0.68
Personal Income ($60,000-$69,999)		0.71	0.56	0.91		0.55	0.41	0.74
Personal Income ($70,000-$79,999)		0.69	0.49	0.96		0.55	0.40	0.76
Personal Income ($80,000-$89,999)		0.77	0.53	1.10		0.77	0.47	1.26
Personal Income ($90,000-$99,999)		0.97	0.65	1.45		0.63	0.39	1.01
Personal Income ($100,000-$149,999)		0.59	0.42	0.82		0.63	0.44	0.89
Personal Income (More than $150,000)		0.93	0.62	1.38		0.44	0.25	0.75
Educational Level (High school graduate)		1.07	0.72	1.58		1.50	0.87	2.61
Educational Level (Some college credit, no degree)		1.05	0.72	1.54		1.67	0.98	2.85
Educational Level (Trade/Technical/Vocational education)		1.16	0.73	1.82		1.90	1.03	3.51
Educational Level (Associate degree)		1.02	0.67	1.54		1.61	0.93	2.76
Educational Level (Bachelor's degree)		0.89	0.61	1.29		1.53	0.90	2.59
Educational Level (Master's degree)		1.08	0.72	1.61		1.61	0.92	2.80
Educational Level (professional degree)		0.73	0.37	1.44		1.83	0.90	3.74
Educational Level (Doctorate degree)		0.93	0.58	1.49		1.73	0.86	3.47
Ethnicity (White)		1.59	1.29	1.96		1.32	1.01	1.73
Ethnicity (Hispanic, Latinx, Spanish)		1.07	0.86	1.33		1.11	0.83	1.47
Ethnicity (Black, African American)		1.31	1.03	1.67		0.94	0.72	1.24
Ethnicity (Asian)		1.16	0.91	1.48		1.09	0.76	1.56
Ethnicity (Native)		1.06	0.80	1.41		1.89	1.27	2.80
Sex (male=0, female=1)		0.94	0.83	1.06		1.21	1.05	1.40
**Goodness of Fit**								
Deviance/df		1.172				1.131		
Pearson Chi-Square/df		1.019				1.104		
Loglikelihood		-5645.574				-5566.070		
AIC		11361.148				11202.139		
**Omnibus tests**								
Chi-square		284.801				315.270		
df		33				33		
N		1896				2004		

Note: Participants reporting Native Hawaiian/Pacific Islander, Middle east/North Africa or Other as ethnicity are the reference group for the ethnicity variables. Personal Income < $10.000 is the reference group for the "Personal Income" variables. Educational level < high school diploma is the reference group for the "Educational Level" variables. The models are computed with robust covariance matrix estimation.

Table S31. Negative binomial regression predicting number of days where poor mental or physical health prevented the respondent from doing usual activities (during past 30 days) (with sex as covariate).

	SAMPLE 1				*SAMPLE 2*		
	Incidence rate ratio	95% CI			Incidence rate ratio	95% CI	
Caregiver strain	1.13	1.04	1.21		1.11	1.03	1.19
Work strain	0.88	0.80	0.96		0.99	0.90	1.08
Independence	1.02	0.94	1.12		0.87	0.77	1.00
Risk-taking	0.93	0.85	1.01		1.02	0.94	1.12
Emotional intelligence	0.98	0.88	1.10		0.92	0.84	1.02
Social support	0.92	0.86	0.99		0.87	0.81	0.94
Discrimination	1.76	1.61	1.93		1.56	1.40	1.73
Year of birth	1.01	1.00	1.01		1.00	1.00	1.01
Personal Income ($10,000-$19,999)	1.01	0.80	1.27		0.91	0.70	1.17
Personal Income ($20,000-$29,999)	0.89	0.70	1.14		0.93	0.73	1.18
Personal Income ($30,000-$39,999)	0.75	0.56	1.01		0.78	0.59	1.02
Personal Income ($40,000-$49,999)	0.91	0.68	1.22		0.65	0.48	0.87
Personal Income ($50,000-$59,999)	0.76	0.56	1.03		0.52	0.37	0.73
Personal Income ($60,000-$69,999)	0.63	0.44	0.89		0.67	0.47	0.97
Personal Income ($70,000-$79,999)	0.66	0.42	1.04		0.62	0.43	0.89
Personal Income ($80,000-$89,999)	1.01	0.59	1.74		0.65	0.40	1.04
Personal Income ($90,000-$99,999)	0.99	0.51	1.93		0.62	0.40	0.95
Personal Income ($100,000-$149,999)	0.90	0.57	1.41		0.70	0.46	1.06
Personal Income (More than $150,000)	1.48	0.88	2.50		0.75	0.38	1.47
Educational Level (High school graduate)	1.08	0.65	1.79		1.00	0.52	1.93
Educational Level (Some college credit, no degree)	0.86	0.53	1.41		1.13	0.61	2.10
Educational Level (Trade/Technical/Vocational education)	1.33	0.75	2.39		0.82	0.40	1.66
Educational Level (Associate degree)	0.84	0.49	1.42		0.96	0.51	1.82
Educational Level (Bachelor's degree)	0.88	0.54	1.43		0.78	0.42	1.44
Educational Level (Master's degree)	0.74	0.44	1.25		0.71	0.37	1.34
Educational Level (professional degree)	0.25	0.11	0.58		0.53	0.23	1.25
Educational Level (Doctorate degree)	0.67	0.37	1.20		0.61	0.28	1.33
Ethnicity (White)	1.15	0.87	1.53		0.90	0.65	1.23
Ethnicity (Hispanic, Latinx, Spanish)	0.90	0.67	1.20		1.01	0.71	1.43
Ethnicity (Black, African American)	1.03	0.74	1.43		0.77	0.55	1.08
Ethnicity (Asian)	0.78	0.57	1.06		0.88	0.57	1.34
Ethnicity (Native)	0.93	0.62	1.40		2.04	1.26	3.30
Sex (male=0, female=1)	0.88	0.74	1.04		1.11	0.94	1.31
**Goodness of Fit**							
Deviance/df	1.081				1.057		
Pearson Chi-Square/df	1.170				1.143		
Loglikelihood	-4564.397				-4630.156		
AIC	9198.795				9330.312		
**Omnibus tests**							
Chi-square	246.377				223.091		
df	33				33		
N	1872				2004		

Note: Participants reporting Native Hawaiian/Pacific Islander, Middle east/North Africa or Other as ethnicity are the reference group for the ethnicity variables. Personal Income < $10.000 is the reference group for the "Personal Income" variables. Educational level < high school diploma is the reference group for the "Educational Level" variables. The models are computed with robust covariance matrix estimation.

Table S32. Logistic regression predicting general health status (excellent, very good, good= 0, fair, poor= 1) (with sex as covariate).

	SAMPLE 1				*SAMPLE 2*			
	Incidence ratio	rate	95% CI		Incidence ratio	rate	95% CI	
Caregiver strain		1.28	1.11	1.47		1.18	1.03	1.34
Work strain		0.88	0.74	1.06		0.98	0.83	1.17
Independence		0.91	0.77	1.07		0.71	0.56	0.89
Risk-taking		0.57	0.48	0.68		0.69	0.58	0.81
Emotional intelligence		0.91	0.76	1.10		0.90	0.75	1.07
Social support		0.83	0.73	0.95		0.78	0.69	0.88
Discrimination		1.43	1.18	1.73		1.55	1.29	1.87
Year of birth		0.98	0.97	0.99		0.98	0.97	0.99
Personal Income ($10,000-$19,999)		0.78	0.52	1.19		1.19	0.78	1.79
Personal Income ($20,000-$29,999)		0.87	0.56	1.34		0.83	0.54	1.29
Personal Income ($30,000-$39,999)		0.60	0.36	1.00		0.83	0.52	1.33
Personal Income ($40,000-$49,999)		0.75	0.45	1.26		0.62	0.37	1.04
Personal Income ($50,000-$59,999)		0.62	0.37	1.05		0.52	0.29	0.93
Personal Income ($60,000-$69,999)		0.85	0.47	1.52		0.78	0.41	1.48
Personal Income ($70,000-$79,999)		0.60	0.28	1.30		0.63	0.33	1.22
Personal Income ($80,000-$89,999)		0.19	0.04	0.81		0.52	0.20	1.34
Personal Income ($90,000-$99,999)		0.30	0.06	1.47		0.96	0.42	2.20
Personal Income ($100,000-$149,999)		0.11	0.02	0.47		0.60	0.30	1.21
Personal Income (More than $150,000)		0.09	0.01	0.74		0.73	0.23	2.33
Educational Level (High school graduate)		0.45	0.19	1.08		0.85	0.28	2.54
Educational Level (Some college credit, no degree)		0.58	0.26	1.33		0.92	0.32	2.65
Educational Level (Trade/Technical/Vocational education)		0.56	0.20	1.60		0.79	0.25	2.52
Educational Level (Associate degree)		0.43	0.17	1.06		1.01	0.34	2.96
Educational Level (Bachelor’s degree)		0.41	0.18	0.94		0.50	0.17	1.44
Educational Level (Master’s degree)		0.47	0.19	1.15		0.39	0.13	1.19
Educational Level (professional degree)		0.35	0.06	2.02		0.25	0.05	1.25
Educational Level (Doctorate degree)		0.18	0.04	0.75		0.63	0.17	2.35
Ethnicity (White)		1.96	1.09	3.52		1.00	0.53	1.88
Ethnicity (Hispanic, Latinx, Spanish)		0.83	0.43	1.63		1.07	0.56	2.05
Ethnicity (Black, African American)		2.22	1.16	4.25		0.91	0.47	1.78
Ethnicity (Asian)		1.68	0.88	3.23		0.79	0.33	1.86
Ethnicity (Native)		0.48	0.19	1.18		0.85	0.37	1.96
Sex (male=0, female=1)		0.60	0.44	0.82		0.65	0.48	0.87
**Goodness of Fit**								
Deviance/df		0.828				0.877		
Pearson Chi-Square/df		1.037				1.013		
Loglikelihood		1600.261				1801.791		
AIC		1601.549				1802.996		
**Omnibus tests**								
		203.535				188.828		
Chi-square								
df		33				33		
N		1884				2010		

Note: Participants reporting Native Hawaiian/Pacific Islander, Middle east/North Africa or Other as ethnicity are the reference group for the ethnicity variables. Personal Income < $10.000 is the reference group for the “Personal Income” variables. Educational level < high school diploma is the reference group for the “Educational Level” variables. The models are computed with robust covariance matrix estimation.

Table S33. Logistic regression predicting smoking (not smoking=0, smoking=1) (with sex as covariate).

	SAMPLE 1			*SAMPLE 2*		
	Incidence rate ratio	95% CI		Incidence rate ratio	95% CI	
Caregiver strain	1.42	1.24	1.62	1.18	1.05	1.33
Work strain	1.37	1.15	1.64	1.26	1.08	1.46
Independence	1.22	1.03	1.46	1.07	0.85	1.35
Risk-taking	1.45	1.21	1.72	1.05	0.91	1.21
Emotional intelligence	1.46	1.22	1.75	1.12	0.96	1.30
Social support	0.98	0.86	1.12	0.97	0.87	1.09
Discrimination	1.09	0.90	1.31	1.16	0.97	1.38
Year of birth	0.97	0.96	0.98	1.00	0.99	1.01
Personal Income ($10,000-$19,999)	1.04	0.63	1.71	1.22	0.80	1.86
Personal Income ($20,000-$29,999)	1.41	0.87	2.28	0.89	0.58	1.37
Personal Income ($30,000-$39,999)	0.82	0.47	1.43	1.16	0.74	1.82
Personal Income ($40,000-$49,999)	1.20	0.69	2.07	0.94	0.59	1.51
Personal Income ($50,000-$59,999)	1.10	0.62	1.94	0.88	0.54	1.46
Personal Income ($60,000-$69,999)	1.31	0.72	2.37	0.86	0.47	1.57
Personal Income ($70,000-$79,999)	1.17	0.59	2.31	1.04	0.58	1.85
Personal Income ($80,000-$89,999)	0.50	0.16	1.59	1.47	0.73	2.95
Personal Income ($90,000-$99,999)	1.94	0.84	4.48	0.64	0.29	1.43
Personal Income ($100,000-$149,999)	0.90	0.47	1.73	0.95	0.52	1.75
Personal Income (More than $150,000)	4.84	2.36	9.94	0.38	0.12	1.21
Educational Level (High school graduate)	1.34	0.43	4.12	0.69	0.25	1.90
Educational Level (Some college credit, no degree)	1.02	0.34	3.08	0.65	0.24	1.75
Educational Level (Trade/Technical/Vocational education)	1.74	0.50	6.06	1.00	0.35	2.89
Educational Level (Associate degree)	0.72	0.23	2.28	0.70	0.26	1.91
Educational Level (Bachelor's degree)	0.49	0.16	1.47	0.30	0.11	0.79
Educational Level (Master's degree)	0.45	0.14	1.40	0.23	0.08	0.66
Educational Level (professional degree)	0.42	0.08	2.25	0.32	0.08	1.26
Educational Level (Doctorate degree)	0.75	0.22	2.60	0.18	0.04	0.78
Ethnicity (White)	1.31	0.65	2.63	0.71	0.40	1.25
Ethnicity (Hispanic, Latinx, Spanish)	0.90	0.48	1.68	0.46	0.24	0.87
Ethnicity (Black, African American)	0.78	0.34	1.79	0.43	0.23	0.81
Ethnicity (Asian)	0.42	0.17	1.04	0.30	0.13	0.70
Ethnicity (Native)	0.74	0.29	1.91	1.65	0.85	3.20
Sex (male=0, female=1)	0.66	0.48	0.89	0.89	0.69	1.16
**Goodness of Fit**						
Deviance/df	0.824			1.007		
Pearson Chi-Square/df	1.042			1.008		
Loglikelihood	-761.542			-994.497		
AIC	1591.084			2056.993		
**Omnibus tests**						
	267.437			143.257		
Chi-square						
df	33			33		
N	1883			2009		

Note: Participants reporting Native Hawaiian/Pacific Islander, Middle east/North Africa or Other as ethnicity are the reference group for the ethnicity variables. Personal Income < $10.000 is the reference group for the "Personal Income" variables. Educational level < high school diploma is the reference group for the "Educational Level" variables. The models are computed with robust covariance matrix estimation.

Table S34. Logistic regression predicting vaping (not vaping=0, vaping=1) (with sex as covariate).

	SAMPLE 1			*SAMPLE 2*		
	Incidence rate ratio	95% CI		Incidence rate ratio	95% CI	
Caregiver strain	1.06	0.90	1.26	1.10	0.93	1.29
Work strain	1.23	0.97	1.55	1.29	1.05	1.59
Independence	1.17	0.95	1.45	1.05	0.76	1.44
Risk-taking	1.20	0.98	1.48	1.00	0.80	1.25
Emotional intelligence	1.39	1.12	1.71	1.05	0.84	1.31
Social support	0.98	0.84	1.14	0.88	0.76	1.04
Discrimination	1.40	1.15	1.70	1.46	1.17	1.81
Year of birth	0.98	0.97	1.00	1.01	1.00	1.03
Personal Income ($10,000-$19,999)	1.97	0.98	3.95	1.20	0.60	2.38
Personal Income ($20,000-$29,999)	1.70	0.85	3.40	1.65	0.88	3.12
Personal Income ($30,000-$39,999)	2.65	1.36	5.14	1.04	0.52	2.09
Personal Income ($40,000-$49,999)	2.11	1.01	4.39	1.09	0.53	2.23
Personal Income ($50,000-$59,999)	2.49	1.23	5.04	1.48	0.72	3.05
Personal Income ($60,000-$69,999)	2.99	1.42	6.29	1.32	0.58	3.01
Personal Income ($70,000-$79,999)	1.72	0.74	3.98	0.90	0.35	2.33
Personal Income ($80,000-$89,999)	0.72	0.16	3.22	1.03	0.32	3.33
Personal Income ($90,000-$99,999)	4.89	1.91	12.55	1.02	0.33	3.14
Personal Income ($100,000-$149,999)	2.26	0.98	5.21	0.91	0.35	2.32
Personal Income (More than $150,000)	7.17	3.21	16.00	0.46	0.06	3.77
Educational Level (High school graduate)	0.35	0.13	0.93	0.85	0.16	4.59
Educational Level (Some college credit, no degree)	0.29	0.11	0.76	0.81	0.16	4.16
Educational Level (Trade/Technical/Vocational education)	0.37	0.10	1.32	0.45	0.07	2.88
Educational Level (Associate degree)	0.28	0.10	0.78	1.17	0.23	6.04
Educational Level (Bachelor's degree)	0.17	0.07	0.44	0.66	0.13	3.35
Educational Level (Master's degree)	0.25	0.09	0.67	0.45	0.08	2.44
Educational Level (professional degree)	0.29	0.06	1.39	0.50	0.06	4.46
Educational Level (Doctorate degree)	0.21	0.06	0.68	0.49	0.06	4.39
Ethnicity (White)	1.85	1.02	3.36	1.05	0.53	2.05
Ethnicity (Hispanic, Latinx, Spanish)	1.23	0.64	2.37	1.36	0.63	2.92
Ethnicity (Black, African American)	1.44	0.67	3.08	0.73	0.35	1.53
Ethnicity (Asian)	1.18	0.58	2.39	0.73	0.29	1.87
Ethnicity (Native)	1.48	0.60	3.61	1.63	0.67	3.95
Sex (male=0, female=1)	0.45	0.31	0.64	0.76	0.52	1.11
**Goodness of Fit**						
Deviance/df	0.643			0.572		
Pearson Chi-Square/df	1.040			1.013		
Loglikelihood	-594.193			-564.518		
AIC	1256.386			1197.036		
**Omnibus tests**						
	169.275			59.470		
Chi-square						
df	33			33		
N	1882			2009		

Note: Participants reporting Native Hawaiian/Pacific Islander, Middle east/North Africa or Other as ethnicity are the reference group for the ethnicity variables. Personal Income < $10.000 is the reference group for the "Personal Income" variables. Educational level < high school diploma is the reference group for the "Educational Level" variables. The models are computed with robust covariance matrix estimation.

Table S35. Logistic regression predicting binge drinking (less than monthly=0, monthly, weekly, and daily=1) (with sex as covariate).

	SAMPLE 1			*SAMPLE 2*		
	Incidence rate ratio	95% CI		Incidence rate ratio	95% CI	
Caregiver strain	0.92	0.81	1.06	0.91	0.80	1.04
Work strain	1.28	1.08	1.51	1.03	0.88	1.21
Independence	1.08	0.93	1.25	1.19	0.94	1.49
Risk-taking	1.53	1.31	1.78	1.25	1.07	1.45
Emotional intelligence	1.18	1.00	1.40	1.04	0.88	1.21
Social support	1.01	0.90	1.13	0.87	0.78	0.97
Discrimination	1.18	1.00	1.39	1.11	0.92	1.32
Year of birth	1.02	1.01	1.03	1.03	1.02	1.04
Personal Income ($10,000-$19,999)	1.02	0.64	1.60	1.26	0.78	2.03
Personal Income ($20,000-$29,999)	1.14	0.73	1.81	1.99	1.26	3.13
Personal Income ($30,000-$39,999)	1.18	0.75	1.87	1.34	0.82	2.20
Personal Income ($40,000-$49,999)	1.29	0.79	2.09	1.81	1.09	2.99
Personal Income ($50,000-$59,999)	0.91	0.55	1.50	1.39	0.81	2.38
Personal Income ($60,000-$69,999)	1.19	0.70	2.03	1.98	1.11	3.55
Personal Income ($70,000-$79,999)	0.81	0.44	1.51	1.44	0.78	2.65
Personal Income ($80,000-$89,999)	1.15	0.52	2.55	1.16	0.52	2.59
Personal Income ($90,000-$99,999)	1.66	0.72	3.81	2.31	1.14	4.67
Personal Income ($100,000-$149,999)	1.13	0.62	2.08	1.95	1.05	3.59
Personal Income (More than $150,000)	2.01	1.01	4.03	2.44	1.01	5.92
Educational Level (High school graduate)	1.22	0.41	3.65	0.50	0.15	1.66
Educational Level (Some college credit, no degree)	1.68	0.58	4.82	0.90	0.29	2.81
Educational Level (Trade/Technical/Vocational education)	2.44	0.73	8.16	0.85	0.25	2.93
Educational Level (Associate degree)	1.37	0.45	4.18	0.65	0.20	2.11
Educational Level (Bachelor’s degree)	1.54	0.54	4.41	0.63	0.20	1.96
Educational Level (Master’s degree)	1.82	0.62	5.34	0.58	0.18	1.89
Educational Level (professional degree)	3.28	0.78	13.78	0.58	0.13	2.50
Educational Level (Doctorate degree)	1.77	0.52	5.95	0.65	0.15	2.74
Ethnicity (White)	1.89	1.08	3.31	0.93	0.54	1.60
Ethnicity (Hispanic, Latinx, Spanish)	1.63	0.96	2.77	0.86	0.50	1.51
Ethnicity (Black, African American)	0.74	0.36	1.53	1.05	0.57	1.93
Ethnicity (Asian)	0.59	0.30	1.13	0.54	0.27	1.09
Ethnicity (Native)	0.45	0.19	1.06	0.79	0.38	1.65
Sex (male=0, female=1)	0.55	0.42	0.71	0.77	0.59	1.01
**Goodness of Fit**						
Deviance/df	1.000			1.002		
Pearson Chi-Square/df	1.017			1.011		
Loglikelihood	-922.640			-988.206		
AIC	1913.280			2044.412		
**Omnibus tests**						
	214.714			116.766		
Chi-square						
df	33			33		
N	1880			2009		

Note: Participants reporting Native Hawaiian/Pacific Islander, Middle east/North Africa or Other as ethnicity are the reference group for the ethnicity variables. Personal Income < $10.000 is the reference group for the “Personal Income” variables. Educational level < high school diploma is the reference group for the “Educational Level” variables. The models are computed with robust covariance matrix estimation.

Table S36. Logistic regression predicting Overweight (BMI<25=0, BMI≥25 =1) (with sex as covariate).

	SAMPLE 1			*SAMPLE 2*		
	Incidence rate ratio	95% CI		Incidence rate ratio	95% CI	
Caregiver strain	1.18	1.05	1.32	1.27	1.13	1.41
Work strain	1.04	0.91	1.19	1.17	1.02	1.33
Independence	0.99	0.88	1.12	1.05	0.87	1.26
Risk-taking	0.82	0.72	0.93	0.85	0.75	0.97
Emotional intelligence	0.99	0.86	1.14	1.03	0.90	1.18
Social support	1.00	0.91	1.10	1.07	0.97	1.18
Discrimination	1.23	1.06	1.42	1.26	1.08	1.48
Year of birth	0.98	0.97	0.99	0.97	0.96	0.98
Personal Income ($10,000-$19,999)	1.12	0.79	1.58	0.83	0.57	1.19
Personal Income ($20,000-$29,999)	1.20	0.84	1.72	0.84	0.58	1.21
Personal Income ($30,000-$39,999)	1.13	0.78	1.63	0.77	0.52	1.13
Personal Income ($40,000-$49,999)	1.15	0.78	1.69	0.77	0.52	1.15
Personal Income ($50,000-$59,999)	1.46	0.97	2.19	1.16	0.75	1.79
Personal Income ($60,000-$69,999)	1.29	0.82	2.02	1.28	0.76	2.16
Personal Income ($70,000-$79,999)	1.27	0.75	2.14	2.21	1.31	3.74
Personal Income ($80,000-$89,999)	1.16	0.59	2.31	0.99	0.54	1.79
Personal Income ($90,000-$99,999)	1.91	0.84	4.32	1.07	0.58	1.97
Personal Income ($100,000-$149,999)	1.02	0.60	1.74	1.01	0.60	1.70
Personal Income (More than $150,000)	1.27	0.65	2.48	0.97	0.44	2.17
Educational Level (High school graduate)	0.86	0.39	1.90	0.24	0.07	0.84
Educational Level (Some college credit, no degree)	0.90	0.42	1.92	0.32	0.09	1.09
Educational Level (Trade/Technical/Vocational education)	0.88	0.34	2.28	0.21	0.06	0.76
Educational Level (Associate degree)	1.06	0.47	2.40	0.23	0.07	0.80
Educational Level (Bachelor's degree)	0.59	0.28	1.26	0.21	0.06	0.72
Educational Level (Master's degree)	0.78	0.35	1.73	0.21	0.06	0.74
Educational Level (professional degree)	0.58	0.18	1.82	0.13	0.03	0.52
Educational Level (Doctorate degree)	0.69	0.27	1.79	0.13	0.03	0.51
Ethnicity (White)	1.08	0.70	1.66	1.03	0.64	1.67
Ethnicity (Hispanic, Latinx, Spanish)	1.28	0.81	2.03	1.60	0.97	2.63
Ethnicity (Black, African American)	1.66	0.99	2.76	1.16	0.67	1.99
Ethnicity (Asian)	0.49	0.30	0.80	0.54	0.30	0.95
Ethnicity (Native)	2.10	1.02	4.34	1.17	0.61	2.23
Sex (male=0, female=1)	0.76	0.60	0.95	0.58	0.46	0.73
**Goodness of Fit**						
Deviance/df	1.313			1.283		
Pearson Chi-Square/df	1.020			1.020		
Loglikelihood	-1197.707			-1256.644		
AIC	2463.413			2581.288		
**Omnibus tests**						
	137.355			163.390		
Chi-square						
df	33			33		
N	1858			1993		

Note: Participants reporting Native Hawaiianå/Pacific Islander, Middle east/North Africa or Other as ethnicity are the reference group for the ethnicity variables. Personal Income < $10.000 is the reference group for the "Personal Income" variables. Educational level < high school diploma is the reference group for the "Educational Level" variables. The models are computed with robust covariance matrix estimation.

Table S37. Negative binomial regression predicting number of days with poor physical health (during past 30 days) (combined samples).

	SEX				*GENDER*			
	Incidence ratio	rate	99.9% CI		Incidence ratio	rate	99.9% CI	
Caregiver strain		1.15	1.06	1.25		1.152	1.063	1.248
Work strain		0.98	0.88	1.08		0.978	0.883	1.084
Independence		0.90	0.80	1.02		0.902	0.801	1.016
Risk-taking		0.98	0.89	1.09		0.979	0.884	1.083
Emotional intelligence		0.94	0.84	1.05		0.938	0.839	1.049
Social support		0.95	0.87	1.03		0.946	0.874	1.025
Discrimination		1.45	1.30	1.61		1.446	1.305	1.603
Year of birth		0.99	0.98	0.99		0.988	0.981	0.994
Personal Income ($10,000-$19,999)		1.06	0.80	1.40		1.063	0.804	1.406
Personal Income ($20,000-$29,999)		0.99	0.75	1.30		0.986	0.748	1.300
Personal Income ($30,000-$39,999)		0.87	0.63	1.19		0.868	0.632	1.191
Personal Income ($40,000-$49,999)		0.85	0.62	1.17		0.852	0.619	1.174
Personal Income ($50,000-$59,999)		0.79	0.54	1.15		0.794	0.545	1.156
Personal Income ($60,000-$69,999)		0.76	0.52	1.12		0.766	0.524	1.118
Personal Income ($70,000-$79,999)		0.70	0.45	1.09		0.701	0.447	1.098
Personal Income ($80,000-$89,999)		0.54	0.33	0.89		0.538	0.327	0.886
Personal Income ($90,000-$99,999)		0.89	0.49	1.60		0.922	0.516	1.647
Personal Income ($100,000-$149,999)		0.72	0.46	1.13		0.721	0.458	1.134
Personal Income (More than $150,000)		0.99	0.56	1.75		1.055	0.598	1.859
Educational Level (High school graduate)		0.95	0.51	1.77		0.951	0.510	1.772
Educational Level (Some college credit, no degree)		0.96	0.53	1.75		0.970	0.535	1.759
Educational Level (Trade/Technical/Vocational education)		0.84	0.43	1.67		0.840	0.425	1.662
Educational Level (Associate degree)		0.88	0.48	1.63		0.877	0.474	1.622
Educational Level (Bachelor's degree)		0.78	0.43	1.41		0.779	0.431	1.408
Educational Level (Master's degree)		0.86	0.46	1.61		0.860	0.459	1.612
Educational Level (professional degree)		0.80	0.30	2.14		0.792	0.295	2.122
Educational Level (Doctorate degree)		0.70	0.33	1.48		0.695	0.328	1.471
Ethnicity (White)		0.98	0.70	1.35		0.950	0.685	1.318
Ethnicity (Hispanic, Latinx, Spanish)		0.97	0.66	1.42		0.953	0.652	1.393
Ethnicity (Black, African American)		0.89	0.62	1.27		0.873	0.610	1.248
Ethnicity (Asian)		0.83	0.55	1.24		0.808	0.538	1.214
Ethnicity (Native)		1.24	0.79	1.94		1.236	0.790	1.935
Sex (male=0, female=1)		0.92	0.77	1.11				
Woman						0.926	0.773	1.109
Non-binary/Gender fluid + Gender=other						0.844	0.381	1.871
Sample (Sample 1=1)		0.82	0.65	1.02		0.816	0.652	1.022
**Goodness of Fit**								
Deviance/df		1.107				1.107		
Pearson Chi-Square/df		1.198				1.197		
Loglikelihood		-10001.663				-10032.722		
AIC		20075.326				20139.445		
**Omnibus tests**								
Chi-square		340.684				345.482		
df		34				35		
N		3879				3889		

Note: Participants reporting Native Hawaiian/Pacific Islander, Middle east/North Africa or Other as ethnicity are the reference group for the ethnicity variables. Personal Income < $10.000 is the reference group for the "Personal Income" variables. Educational level < high school diploma is the reference group for the "Educational Level" variables. The models are computed with robust covariance matrix estimation.

Table S38. Negative binomial regression predicting number of days with mental health (during past 30 days) (combined samples).

	SEX			*GENDER*		
	Incidence rate ratio	99.9% CI		Incidence rate ratio	99.9% CI	
Caregiver strain	1.20	1.12	1.28	1.20	1.13	1.28
Work strain	1.07	0.98	1.17	1.07	0.98	1.17
Independence	0.93	0.84	1.02	0.93	0.84	1.02
Risk-taking	0.92	0.84	1.01	0.92	0.84	1.01
Emotional intelligence	0.91	0.83	1.01	0.91	0.83	1.01
Social support	0.83	0.78	0.89	0.84	0.78	0.90
Discrimination	1.40	1.28	1.54	1.40	1.28	1.53
Year of birth	1.02	1.01	1.02	1.02	1.01	1.02
Personal Income ($10,000-$19,999)	0.99	0.79	1.23	0.99	0.79	1.23
Personal Income ($20,000-$29,999)	0.83	0.65	1.06	0.84	0.66	1.07
Personal Income ($30,000-$39,999)	0.81	0.63	1.04	0.81	0.63	1.04
Personal Income ($40,000-$49,999)	0.69	0.53	0.90	0.69	0.53	0.90
Personal Income ($50,000-$59,999)	0.66	0.49	0.90	0.66	0.49	0.90
Personal Income ($60,000-$69,999)	0.65	0.47	0.90	0.65	0.47	0.90
Personal Income ($70,000-$79,999)	0.61	0.41	0.90	0.61	0.41	0.90
Personal Income ($80,000-$89,999)	0.76	0.44	1.30	0.75	0.44	1.28
Personal Income ($90,000-$99,999)	0.78	0.46	1.34	0.77	0.45	1.32
Personal Income ($100,000-$149,999)	0.63	0.41	0.96	0.63	0.41	0.95
Personal Income (More than $150,000)	0.76	0.44	1.30	0.79	0.46	1.36
Educational Level (High school graduate)	1.14	0.65	2.01	1.14	0.65	2.00
Educational Level (Some college credit, no degree)	1.18	0.69	2.04	1.18	0.68	2.03
Educational Level (Trade/Technical/Vocational education)	1.32	0.69	2.53	1.32	0.69	2.53
Educational Level (Associate degree)	1.13	0.64	1.99	1.12	0.64	1.98
Educational Level (Bachelor's degree)	1.02	0.60	1.75	1.02	0.59	1.75
Educational Level (Master's degree)	1.17	0.66	2.07	1.17	0.66	2.07
Educational Level (professional degree)	1.05	0.46	2.43	1.04	0.45	2.40
Educational Level (Doctorate degree)	1.15	0.58	2.29	1.14	0.57	2.27
Ethnicity (White)	1.37	1.03	1.83	1.34	1.00	1.79
Ethnicity (Hispanic, Latinx, Spanish)	1.07	0.80	1.43	1.06	0.79	1.42
Ethnicity (Black, African American)	1.07	0.78	1.45	1.05	0.77	1.43
Ethnicity (Asian)	1.04	0.73	1.48	1.02	0.72	1.46
Ethnicity (Native)	1.47	0.93	2.30	1.46	0.93	2.29
Sex (male=0, female=1)	1.05	0.89	1.23			
Woman				1.05	0.89	1.23
Non-binary/Gender fluid + Gender=other				1.23	0.78	1.93
Sample (Sample 1=1)	0.90	0.75	1.09	0.91	0.75	1.09
**Goodness of Fit**						
Deviance/df	1.145			1.146		
Pearson Chi-Square/df	1.059			1.058		
Loglikelihood	-11254.537			-11285.196		
AIC	22581.073			22644.392		
**Omnibus tests**						
	567.157			568.564		
Chi-square						
df	34			35		
	3880			3890		
N						

Table S39. Negative binomial regression predicting number of days where poor mental or physical health prevented the respondent from doing usual activities (during past 30 days) (combined samples).

	SEX			*GENDER*		
	Incidence rate ratio	99.9% CI		Incidence rate ratio	99.9% CI	
Caregiver strain	1.12	1.02	1.23	1.12	1.02	1.23
Work strain	0.94	0.84	1.05	0.94	0.84	1.05
Independence	0.97	0.86	1.10	0.98	0.86	1.11
Risk-taking	0.98	0.88	1.10	0.98	0.88	1.09
Emotional intelligence	0.96	0.85	1.09	0.96	0.85	1.09
Social support	0.90	0.82	0.98	0.90	0.82	0.98
Discrimination	1.65	1.47	1.86	1.65	1.47	1.85
Year of birth	1.00	1.00	1.01	1.00	1.00	1.01
Personal Income ($10,000-$19,999)	0.96	0.72	1.29	0.96	0.72	1.29
Personal Income ($20,000-$29,999)	0.92	0.69	1.24	0.93	0.70	1.24
Personal Income ($30,000-$39,999)	0.76	0.55	1.07	0.77	0.55	1.07
Personal Income ($40,000-$49,999)	0.75	0.53	1.07	0.76	0.53	1.08
Personal Income ($50,000-$59,999)	0.62	0.42	0.91	0.63	0.43	0.92
Personal Income ($60,000-$69,999)	0.63	0.41	0.97	0.64	0.42	0.98
Personal Income ($70,000-$79,999)	0.63	0.38	1.03	0.64	0.39	1.04
Personal Income ($80,000-$89,999)	0.76	0.42	1.38	0.76	0.42	1.38
Personal Income ($90,000-$99,999)	0.77	0.40	1.47	0.84	0.44	1.62
Personal Income ($100,000-$149,999)	0.80	0.47	1.36	0.80	0.47	1.37
Personal Income (More than $150,000)	1.15	0.59	2.22	1.21	0.63	2.32
Educational Level (High school graduate)	1.02	0.51	2.02	1.02	0.51	2.02
Educational Level (Some college credit, no degree)	0.97	0.51	1.86	0.97	0.51	1.88
Educational Level (Trade/Technical/Vocational education)	0.96	0.45	2.03	0.96	0.45	2.03
Educational Level (Associate degree)	0.88	0.44	1.74	0.87	0.44	1.74
Educational Level (Bachelor’s degree)	0.81	0.42	1.54	0.80	0.42	1.54
Educational Level (Master’s degree)	0.72	0.36	1.43	0.71	0.36	1.41
Educational Level (professional degree)	0.41	0.15	1.11	0.40	0.15	1.07
Educational Level (Doctorate degree)	0.66	0.30	1.46	0.65	0.30	1.43
Ethnicity (White)	0.98	0.70	1.38	0.96	0.68	1.35
Ethnicity (Hispanic, Latinx, Spanish)	0.93	0.63	1.36	0.92	0.62	1.34
Ethnicity (Black, African American)	0.86	0.59	1.28	0.85	0.57	1.25
Ethnicity (Asian)	0.76	0.50	1.16	0.75	0.49	1.13
Ethnicity (Native)	1.45	0.80	2.61	1.44	0.80	2.60
Sex (male=0, female=1)	0.99	0.81	1.21			
Woman				0.99	0.81	1.20
Non-binary/Gender fluid + Gender=other				1.18	0.56	2.49
Sample (Sample 1=1)	0.94	0.74	1.20	0.95	0.74	1.21
**Goodness of Fit**						
Deviance/df	1.059			1.060		
Pearson Chi-Square/df	1.148			1.145		
Loglikelihood	-9221.048			-9251.865		
AIC	18514.096			18577.731		
**Omnibus tests**						
	425.819			428.103		
Chi-square						
df	34			35		
N	3876			3886		

Note: Participants reporting Native Hawaiian/Pacific Islander, Middle east/North Africa or Other as ethnicity are the reference group for the ethnicity variables. Personal Income < $10.000 is the reference group for the “Personal Income” variables. Educational level < high school diploma is the reference group for the “Educational Level” variables. The models are computed with robust covariance matrix estimation.

Table S40. Logistic regression predicting general health status (excellent, very good, good= 0, fair, poor= 1) (combined samples).

	SEX			*GENDER*		
	Odds ratio	99.9% CI		Odds ratio	99.9% CI	
Caregiver strain	1.21	1.04	1.42	1.21	1.03	1.42
Work strain	0.93	0.76	1.15	0.93	0.76	1.14
Independence	0.83	0.66	1.03	0.83	0.66	1.03
Risk-taking	0.63	0.52	0.77	0.62	0.51	0.76
Emotional intelligence	0.89	0.72	1.10	0.89	0.72	1.11
Social support	0.81	0.70	0.94	0.81	0.70	0.94
Discrimination	1.48	1.19	1.85	1.49	1.20	1.86
Year of birth	0.98	0.97	0.99	0.98	0.97	0.99
Personal Income ($10,000-$19,999)	0.95	0.59	1.54	0.95	0.59	1.54
Personal Income ($20,000-$29,999)	0.81	0.49	1.35	0.81	0.49	1.35
Personal Income ($30,000-$39,999)	0.71	0.40	1.25	0.71	0.41	1.25
Personal Income ($40,000-$49,999)	0.67	0.37	1.23	0.67	0.37	1.23
Personal Income ($50,000-$59,999)	0.57	0.29	1.09	0.57	0.29	1.09
Personal Income ($60,000-$69,999)	0.79	0.38	1.62	0.79	0.39	1.63
Personal Income ($70,000-$79,999)	0.61	0.27	1.38	0.61	0.27	1.38
Personal Income ($80,000-$89,999)	0.35	0.10	1.29	0.35	0.10	1.29
Personal Income ($90,000-$99,999)	0.66	0.20	2.12	0.69	0.22	2.12
Personal Income ($100,000-$149,999)	0.37	0.14	0.97	0.37	0.14	0.98
Personal Income (More than $150,000)	0.30	0.06	1.58	0.30	0.06	1.59
Educational Level (High school graduate)	0.59	0.19	1.84	0.59	0.19	1.85
Educational Level (Some college credit, no degree)	0.71	0.24	2.10	0.71	0.24	2.10
Educational Level (Trade/Technical/Vocational education)	0.65	0.18	2.31	0.65	0.18	2.31
Educational Level (Associate degree)	0.68	0.22	2.10	0.68	0.22	2.10
Educational Level (Bachelor's degree)	0.43	0.15	1.28	0.44	0.15	1.29
Educational Level (Master's degree)	0.40	0.13	1.28	0.40	0.12	1.28
Educational Level (professional degree)	0.27	0.04	1.87	0.27	0.04	1.87
Educational Level (Doctorate degree)	0.33	0.07	1.49	0.32	0.07	1.49
Ethnicity (White)	1.31	0.64	2.67	1.31	0.64	2.67
Ethnicity (Hispanic, Latinx, Spanish)	0.95	0.44	2.04	0.95	0.44	2.05
Ethnicity (Black, African American)	1.30	0.61	2.79	1.33	0.62	2.84
Ethnicity (Asian)	1.14	0.48	2.67	1.13	0.48	2.65
Ethnicity (Native)	0.69	0.26	1.86	0.69	0.26	1.86
Sex (male=0, female=1)	0.64	0.45	0.91			
Woman				0.62	0.44	0.88
Non-binary/Gender fluid + Gender=other				0.84	0.19	3.72
Sample (Sample 1=1)	0.79	0.52	1.18	0.79	0.52	1.18
**Goodness of Fit**						
Deviance/df	0.857			0.857		
Pearson Chi-Square/df	1.006			1.010		
Loglikelihood	-1656.691			-1653.960		
AIC	3385.382			3377.919		
**Omnibus tests**				351.378		
	358.997			351.378		
Chi-square						
df				34		
				3904		
N	3894					

Note: Participants reporting Native Hawaiian/Pacific Islander, Middle east/North Africa or Other as ethnicity are the reference group for the ethnicity variables. Personal Income < $10.000 is the reference group for the "Personal Income" variables. Educational level < high school diploma is the reference group for the "Educational Level" variables. The models are computed with robust covariance matrix estimation.

Table S41. Logistic regression predicting smoking (not smoking=0, smoking=1) (combined samples).

	SEX			*GENDER*		
	Odds ratio	99.9% CI		Odds ratio	99.9% CI	
Caregiver strain	1.29	1.12	1.50	1.29	1.12	1.50
Work strain	1.31	1.08	1.58	1.30	1.08	1.57
Independence	1.19	0.95	1.48	1.19	0.95	1.49
Risk-taking	1.20	1.00	1.44	1.21	1.01	1.45
Emotional intelligence	1.28	1.06	1.55	1.28	1.06	1.55
Social support	0.98	0.85	1.12	0.97	0.85	1.12
Discrimination	1.16	0.94	1.43	1.17	0.95	1.44
Year of birth	0.99	0.98	1.00	0.99	0.98	1.00
Personal Income ($10,000-$19,999)	1.21	0.71	2.05	1.22	0.72	2.08
Personal Income ($20,000-$29,999)	1.13	0.67	1.93	1.13	0.66	1.93
Personal Income ($30,000-$39,999)	1.08	0.61	1.92	1.10	0.62	1.94
Personal Income ($40,000-$49,999)	1.04	0.57	1.88	1.04	0.57	1.88
Personal Income ($50,000-$59,999)	1.01	0.54	1.88	1.01	0.54	1.89
Personal Income ($60,000-$69,999)	1.08	0.53	2.17	1.08	0.54	2.18
Personal Income ($70,000-$79,999)	1.09	0.52	2.28	1.10	0.53	2.29
Personal Income ($80,000-$89,999)	1.05	0.40	2.73	1.06	0.41	2.75
Personal Income ($90,000-$99,999)	1.00	0.37	2.71	0.98	0.36	2.65
Personal Income ($100,000-$149,999)	0.99	0.47	2.09	0.99	0.47	2.10
Personal Income (More than $150,000)	2.53	1.07	6.01	2.46	1.03	5.84
Educational Level (High school graduate)	1.00	0.29	3.42	1.01	0.29	3.44
Educational Level (Some college credit, no degree)	0.87	0.26	2.89	0.88	0.26	2.92
Educational Level (Trade/Technical/Vocational education)	1.37	0.36	5.15	1.37	0.36	5.14
Educational Level (Associate degree)	0.82	0.24	2.83	0.82	0.24	2.82
Educational Level (Bachelor's degree)	0.40	0.12	1.33	0.40	0.12	1.34
Educational Level (Master's degree)	0.39	0.11	1.34	0.39	0.11	1.35
Educational Level (professional degree)	0.41	0.07	2.29	0.42	0.08	2.31
Educational Level (Doctorate degree)	0.52	0.12	2.20	0.53	0.12	2.24
Ethnicity (White)	0.89	0.44	1.83	0.90	0.44	1.85
Ethnicity (Hispanic, Latinx, Spanish)	0.62	0.29	1.31	0.62	0.30	1.31
Ethnicity (Black, African American)	0.51	0.22	1.18	0.51	0.22	1.19
Ethnicity (Asian)	0.31	0.11	0.84	0.31	0.11	0.83
Ethnicity (Native)	1.24	0.54	2.89	1.27	0.54	2.97
Sex (male=0, female=1)	0.77	0.56	1.07			
Woman				0.78	0.56	1.07
Non-binary/Gender fluid + Gender=other				0.19	0.02	2.07
Sample (Sample 1=1)	1.44	0.94	2.20	1.45	0.95	2.22
**Goodness of Fit**						
Deviance/df	0.936			0.934		
Pearson Chi-Square/df	0.986			0.983		
Loglikelihood	-1804.942			-1804.685		
AIC	3679.885			3681.370		
**Omnibus tests**						
	322.659			329.998		
Chi-square						
df	34			35		
N	3892			3901		

Note: Participants reporting Native Hawaiian/Pacific Islander, Middle east/North Africa or Other as ethnicity are the reference group for the ethnicity variables. Personal Income < $10.000 is the reference group for the "Personal Income" variables. Educational level < high school diploma is the reference group for the "Educational Level" variables. The models are computed with robust covariance matrix estimation.

Table S42. Logistic regression predicting vaping (not vaping=0, vaping=1) (combined samples).

	SEX			*GENDER*		
	Odds ratio	99.9% CI		Odds ratio	99.9% CI	
Caregiver strain	1.10	0.91	1.32	1.10	0.91	1.32
Work strain	1.27	0.98	1.64	1.26	0.98	1.63
Independence	1.15	0.86	1.52	1.15	0.87	1.53
Risk-taking	1.10	0.86	1.41	1.10	0.86	1.40
Emotional intelligence	1.25	0.97	1.60	1.25	0.97	1.60
Social support	0.93	0.78	1.11	0.93	0.78	1.12
Discrimination	1.44	1.13	1.82	1.44	1.14	1.83
Year of birth	1.00	0.99	1.02	1.00	0.99	1.02
Personal Income ($10,000-$19,999)	1.59	0.70	3.57	1.55	0.70	3.47
Personal Income ($20,000-$29,999)	1.80	0.83	3.90	1.75	0.81	3.76
Personal Income ($30,000-$39,999)	1.70	0.76	3.78	1.65	0.75	3.65
Personal Income ($40,000-$49,999)	1.50	0.63	3.56	1.46	0.62	3.45
Personal Income ($50,000-$59,999)	1.93	0.83	4.49	1.89	0.82	4.36
Personal Income ($60,000-$69,999)	2.00	0.80	4.99	1.94	0.78	4.82
Personal Income ($70,000-$79,999)	1.27	0.44	3.64	1.23	0.43	3.51
Personal Income ($80,000-$89,999)	0.89	0.19	4.08	0.86	0.19	3.94
Personal Income ($90,000-$99,999)	2.26	0.68	7.48	2.17	0.66	7.13
Personal Income ($100,000-$149,999)	1.53	0.55	4.25	1.49	0.54	4.11
Personal Income (More than $150,000)	4.37	1.60	11.96	4.41	1.63	11.95
Educational Level (High school graduate)	0.48	0.12	1.93	0.48	0.12	1.93
Educational Level (Some college credit, no degree)	0.43	0.11	1.65	0.44	0.12	1.67
Educational Level (Trade/Technical/Vocational education)	0.34	0.06	1.84	0.35	0.06	1.86
Educational Level (Associate degree)	0.54	0.14	2.14	0.55	0.14	2.16
Educational Level (Bachelor's degree)	0.31	0.08	1.17	0.32	0.08	1.20
Educational Level (Master's degree)	0.35	0.09	1.40	0.36	0.09	1.43
Educational Level (professional degree)	0.35	0.05	2.53	0.35	0.05	2.53
Educational Level (Doctorate degree)	0.36	0.07	1.93	0.37	0.07	1.97
Ethnicity (White)	1.38	0.65	2.95	1.31	0.61	2.79
Ethnicity (Hispanic, Latinx, Spanish)	1.28	0.55	2.97	1.25	0.53	2.91
Ethnicity (Black, African American)	1.00	0.41	2.45	1.01	0.41	2.46
Ethnicity (Asian)	0.85	0.33	2.20	0.80	0.30	2.10
Ethnicity (Native)	1.55	0.55	4.33	1.58	0.56	4.41
Sex (male=0, female=1)	0.57	0.37	0.88			
Woman				0.56	0.37	0.86
Non-binary/Gender fluid + Gender=other				0.42	0.05	3.62
Sample (Sample 1=1)	0.89	0.51	1.55	0.89	0.52	1.55
**Goodness of Fit**						
Deviance/df	0.618			0.618		
Pearson Chi-Square/df	1.027			1.033		
Loglikelihood	2451.495			-1194.642		
AIC	2452.148			2461.285		
**Omnibus tests**						
	174.129			176.985		
Chi-square						
df	34			35		
N	3891			3900		

Note: Participants reporting Native Hawaiian/Pacific Islander, Middle east/North Africa or Other as ethnicity are the reference group for the ethnicity variables. Personal Income < $10.000 is the reference group for the "Personal Income" variables. Educational level < high school diploma is the reference group for the "Educational Level" variables. The models are computed with robust covariance matrix estimation.

Table S43. Logistic regression predicting binge drinking (less than monthly=0, monthly, weekly, and daily=1) (combined samples).

	SEX			*GENDER*		
	Odds ratio	99.9% CI		Odds ratio	99.9% CI	
Caregiver strain	0.93	0.80	1.09	0.93	0.79	1.09
Work strain	1.15	0.95	1.39	1.15	0.95	1.39
Independence	1.12	0.92	1.36	1.12	0.91	1.36
Risk-taking	1.37	1.15	1.64	1.37	1.15	1.64
Emotional intelligence	1.12	0.93	1.35	1.12	0.93	1.35
Social support	0.94	0.82	1.07	0.93	0.82	1.07
Discrimination	1.16	0.95	1.42	1.16	0.96	1.42
Year of birth	1.02	1.01	1.04	1.02	1.01	1.04
Personal Income ($10,000-$19,999)	1.16	0.67	2.00	1.16	0.67	2.01
Personal Income ($20,000-$29,999)	1.60	0.94	2.70	1.59	0.94	2.68
Personal Income ($30,000-$39,999)	1.29	0.74	2.26	1.29	0.74	2.26
Personal Income ($40,000-$49,999)	1.58	0.89	2.80	1.58	0.89	2.81
Personal Income ($50,000-$59,999)	1.14	0.63	2.09	1.15	0.63	2.11
Personal Income ($60,000-$69,999)	1.54	0.80	2.95	1.53	0.80	2.95
Personal Income ($70,000-$79,999)	1.11	0.54	2.28	1.11	0.54	2.28
Personal Income ($80,000-$89,999)	1.19	0.47	3.02	1.19	0.47	3.03
Personal Income ($90,000-$99,999)	1.91	0.80	4.56	1.91	0.80	4.56
Personal Income ($100,000-$149,999)	1.50	0.74	3.04	1.50	0.74	3.05
Personal Income (More than $150,000)	2.72	1.16	6.35	2.74	1.17	6.41
Educational Level (High school graduate)	0.81	0.22	2.98	0.80	0.22	2.97
Educational Level (Some college credit, no degree)	1.28	0.37	4.49	1.29	0.37	4.53
Educational Level (Trade/Technical/Vocational education)	1.39	0.34	5.62	1.38	0.34	5.59
Educational Level (Associate degree)	1.00	0.27	3.67	1.00	0.27	3.65
Educational Level (Bachelor's degree)	1.04	0.30	3.63	1.05	0.30	3.65
Educational Level (Master's degree)	1.12	0.31	4.03	1.12	0.31	4.05
Educational Level (professional degree)	1.37	0.26	7.05	1.37	0.26	7.04
Educational Level (Doctorate degree)	1.22	0.27	5.46	1.24	0.28	5.52
Ethnicity (White)	1.35	0.72	2.55	1.34	0.71	2.53
Ethnicity (Hispanic, Latinx, Spanish)	1.19	0.63	2.23	1.18	0.63	2.23
Ethnicity (Black, African American)	0.98	0.46	2.06	0.97	0.46	2.05
Ethnicity (Asian)	0.53	0.25	1.17	0.53	0.24	1.15
Ethnicity (Native)	0.62	0.24	1.59	0.64	0.25	1.64
Sex (male=0, female=1)	0.65	0.48	0.89			
Woman				0.67	0.49	0.91
Non-binary/Gender fluid + Gender=other				0.34	0.07	1.74
Sample (Sample 1=1)	1.18	0.80	1.74	1.18	0.80	1.74
**Goodness of Fit**						
Deviance/df	1.005			1.005		
Pearson Chi-Square/df	1.015			1.014		
Loglikelihood	-1936.517			-1936.050		
AIC	3943.033			3944.100		
**Omnibus tests**						
	282.699			283.632		
Chi-square						
df	34			35		
N	3887			3887		

Note: Participants reporting Native Hawaiian/Pacific Islander, Middle east/North Africa or Other as ethnicity are the reference group for the ethnicity variables. Personal Income < $10.000 is the reference group for the "Personal Income" variables. Educational level < high school diploma is the reference group for the "Educational Level" variables. The models are computed with robust covariance matrix estimation.

Table S44. Logistic regression predicting Overweight (BMI<25=0, BMI≥25 =1) (combined samples).

	SEX			*GENDER*		
	Odds ratio	99.9% CI		Odds ratio	99.9% CI	
Caregiver strain	1.21	1.07	1.38	1.22	1.07	1.38
Work strain	1.10	0.95	1.29	1.10	0.94	1.29
Independence	1.01	0.85	1.20	1.01	0.85	1.19
Risk-taking	0.84	0.72	0.97	0.84	0.72	0.97
Emotional intelligence	1.00	0.85	1.18	1.00	0.85	1.18
Social support	1.04	0.93	1.17	1.04	0.93	1.16
Discrimination	1.24	1.03	1.48	1.23	1.03	1.47
Year of birth	0.97	0.97	0.98	0.97	0.97	0.98
Personal Income ($10,000-$19,999)	0.96	0.63	1.46	0.97	0.64	1.47
Personal Income ($20,000-$29,999)	1.01	0.66	1.53	1.02	0.67	1.55
Personal Income ($30,000-$39,999)	0.92	0.59	1.43	0.94	0.61	1.47
Personal Income ($40,000-$49,999)	0.93	0.59	1.48	0.95	0.60	1.50
Personal Income ($50,000-$59,999)	1.30	0.79	2.13	1.32	0.81	2.17
Personal Income ($60,000-$69,999)	1.28	0.72	2.25	1.30	0.74	2.30
Personal Income ($70,000-$79,999)	1.71	0.94	3.12	1.74	0.96	3.18
Personal Income ($80,000-$89,999)	1.08	0.51	2.29	1.10	0.52	2.33
Personal Income ($90,000-$99,999)	1.38	0.62	3.09	1.39	0.63	3.07
Personal Income ($100,000-$149,999)	1.03	0.55	1.90	1.05	0.57	1.94
Personal Income (More than $150,000)	1.09	0.47	2.54	1.08	0.47	2.51
Educational Level (High school graduate)	0.59	0.20	1.72	0.59	0.20	1.72
Educational Level (Some college credit, no degree)	0.68	0.24	1.92	0.68	0.24	1.92
Educational Level (Trade/Technical/Vocational education)	0.53	0.16	1.73	0.53	0.16	1.72
Educational Level (Associate degree)	0.60	0.21	1.77	0.60	0.20	1.75
Educational Level (Bachelor's degree)	0.45	0.16	1.28	0.45	0.16	1.27
Educational Level (Master's degree)	0.52	0.18	1.52	0.51	0.17	1.50
Educational Level (professional degree)	0.33	0.08	1.30	0.33	0.08	1.30
Educational Level (Doctorate degree)	0.39	0.11	1.38	0.39	0.11	1.37
Ethnicity (White)	1.05	0.61	1.81	1.07	0.62	1.85
Ethnicity (Hispanic, Latinx, Spanish)	1.42	0.80	2.50	1.44	0.82	2.53
Ethnicity (Black, African American)	1.38	0.74	2.58	1.39	0.75	2.59
Ethnicity (Asian)	0.50	0.27	0.94	0.51	0.27	0.95
Ethnicity (Native)	1.55	0.69	3.44	1.57	0.70	3.50
Sex (male=0, female=1)	0.67	0.51	0.87			
Woman				0.68	0.52	0.88
Non-binary/Gender fluid + Gender=other				0.76	0.24	2.42
Sample (Sample 1=1)	0.90	0.65	1.24	0.90	0.66	1.25
**Goodness of Fit**						
Deviance/df	1.296			1.296		
Pearson Chi-Square/df	1.010			1.010		
Loglikelihood	-2472.355			-2478.706		
AIC	5014.711			5029.412		
**Omnibus tests**						
	267.790			268.953		
Chi-square						
df	34			35		
N	3851			3860		

Note: Participants reporting Native Hawaiian/Pacific Islander, Middle east/North Africa or Other as ethnicity are the reference group for the ethnicity variables. Personal Income < $10.000 is the reference group for the "Personal Income" variables. Educational level < high school diploma is the reference group for the "Educational Level" variables. The models are computed with robust covariance matrix estimation.

Table S45. Final 25 survey items

1. Please specify your current employment status:

- ☐ Employed Full-time
- ☐ Employed Part-time
- ☐ Homemaker
- ☐ Retired
- ☐ Student
- ☐ Unemployed

2. People see themselves in different ways. There are no right or wrong answers to the following questions. We just want to know what's true for *you*. Choose the answer that best describes you.

i. In general, how prepared are you to take risks?

- Not at all prepared
- Slightly prepared
- Moderately prepared
- Very prepared
- Completely prepared

ii. How prepared are you to take risks….

	Not at all prepared	Slightly prepared	Moderately prepared	Very prepared	Completely prepared
...when making financial decisions?	○	○	○	○	○
...when it comes to recreational activities?	○	○	○	○	○

3. People see themselves in different ways. There are no right or wrong answers to the following questions. We just want to know what's true for *you*. Choose the answer that best describes you.

i. How important is it for you to be independent?

- ◯ Extremely important
- ◯ Very important
- ◯ Moderately important
- ◯ Slightly important
- ◯ Not at all important

iii. How important is it for you to solve your problems on your own?

- ◯ Extremely important
- ◯ Very important
- ◯ Moderately important
- ◯ Slightly important
- ◯ Not at all important

4. People see themselves in different ways. There are no right or wrong answers to the following questions. We just want to know what's true for *you*. Choose the answer that best describes you.

i. How often do friends talk to you about their problems?

- ○ Never
- ○ Once in a while
- ○ Sometimes
- ○ Most of the time
- ○ Always

ii. How often do you talk to your friends about your problems?

- ○ Never
- ○ Once in a while
- ○ Sometimes
- ○ Most of the time
- ○ Always

ii. How easy is it for you to express what you are feeling to others?

- ○ Not at all easy
- ○ Slightly easy
- ○ Moderately easy
- ○ Very easy
- ○ Extremely easy

5. Are you currently responsible for taking care of someone in need? By "taking care of someone in need" we mean providing unpaid assistance and support to someone who has physical or psychological needs, such as a child, elder, partner, or disabled family member.

- ◯ I am currently responsible for taking care of someone in need
- ◯ I have been responsible for taking care of someone in the past
- ◯ I have never been responsible for taking care of someone in need
- ◯ I am currently responsible for taking care of someone in need, and I have been responsible for taking care of someone in the past

[BRANCHING: These next three questions should be displayed **only** to respondents who are currently responsible for taking care of someone in need. Respondents who are branched out should be assigned the value "Never" in the final coding as responses to these questions].

6. In the past year, how often did you feel **emotionally** exhausted because of your caretaking responsibilities?

- ◯ Never
- ◯ Once in a while
- ◯ Sometimes
- ◯ Most of the time
- ◯ Always

7. In the past year, how often did you feel **physically** exhausted because of your caretaking responsibilities?

- ◯ Never
- ◯ Once in a while
- ◯ Sometimes
- ◯ Most of the time
- ◯ Always

8. In the past year, how often have your caretaking responsibilities caused you to worry about the future?

- ○ Never
- ○ Once in a while
- ○ Sometimes
- ○ Most of the time
- ○ Always

9. We are interested in how you spend your time on an average weekday, Monday through Friday. Please provide your best estimate.
On average, how many hours per weekday do you spend on the following. *Total should not exceed **24 hours**.*
Work (paid work, studying, internships, etc.) : _______
Taking care of someone in need (caring for children, elders, partners in need, etc.) : _______

10. We are interested in how you feel about your current job, including your daily work activities as an employee or student. For each of the following questions, select the answer that best describes your work activities. If you have several jobs, please think about the job that you spend most hours doing per week.

[BRANCHING: The next four questions should be displayed **only** to respondents who are currently employed (full-time or part-time) or students. Respondents who are branched out should be assigned the value "Never" in the final coding as responses to these questions].

i. How often does your job require working fast?

- ○ Never
- ○ Once in a while
- ○ Sometimes
- ○ Most of the time
- ○ Always

ii. How often does your job involve repetitive tasks?

- ○ Never
- ○ Once in a while
- ○ Sometimes
- ○ Most of the time
- ○ Always

iii. How often do you feel **emotionally** exhausted from your work activities?

- ○ Never
- ○ Once in a while
- ○ Sometimes
- ○ Most of the time
- ○ Always

iv. How often do you feel **physically** exhausted from your work activities?

- Never
- Once in a while
- Sometimes
- Most of the time
- Always

11. People sometimes look to others for companionship, assistance, or other types of physical or emotional support. The following questions ask you about the support available to you when you need it. Choose the answer that best describes your situation.

i. In the past year, how often did you have someone…

	Never	Once in a while	Sometimes	Most of the time	Always
to show you love and affection?	○	○	○	○	○
to help you with daily chores?	○	○	○	○	○

12. The following questions ask about how often you have felt discriminated against because of your gender. You may not be certain about your answers to these questions, but we would like you to choose the answer that best describes your experience.

i. Because of your gender, how often have you felt discriminated against?

- Never
- Once in a while
- Sometimes
- Most of the time
- Always

ii. Because of your gender how often have you felt discriminated against…

	Never	Once in a while	Sometimes	Most of the time	Always
When getting hired	○	○	○	○	○
When at school	○	○	○	○	○
When receiving medical care	○	○	○	○	○
In public settings	○	○	○	○	○
In your family	○	○	○	○	○

13. What was your birth sex?

○ Male

○ Female

○ Intersex

○ Other: Please specify: ____

○ Prefer not to state

14. What is your gender? Please select all that apply.

☐ Man

☐ Woman

☐ Gender fluid/Non-binary

☐ Other. Please specify: ____

☐ Prefer not to state

15. In what year were you born?

▼ 2000 ... 1900

16. Which categories describe you? (You may choose more than one.)

- ☐ White
- ☐ Hispanic, Latino, or Spanish
- ☐ Black or African American
- ☐ Asian
- ☐ Native American or Alaska Native
- ☐ Middle Eastern or North African
- ☐ Native Hawaiian or Pacific Islander
- ☐ Other

17. What was your income last calendar year?
Please combine all incomes. “Incomes” include wages, salaries, small business earnings, social security, armed forces pay, special cash bonuses and subsistence allowances.

- Less than $10,000
- $10,000 - $19,999
- $20,000 - $29,999
- $30,000 - $39,999
- $40,000 - $49,999
- $50,000 - $59,999
- $60,000 - $69,999
- $70,000 - $79,999
- $80,000 - $89,999
- $90,000 - $99,999
- $100,000 - $149,999
- $150,000 - $199,999
- More than $200,000
- Prefer not to say

18. What is the highest degree or level of school you have completed? If currently enrolled, please report the highest degree received.

- No schooling completed
- Preschool to 8th grade
- Some high school, no diploma
- High school graduate, diploma or equivalent (GED)
- Some college credit, no degree

- Trade/technical/vocational training
- Associate degree
- Bachelor’s degree
- Master’s degree
- Professional degree
- Doctorate degree
